# Multivariate and Cladistic Analyses of Isolated Teeth Reveal Sympatry of Theropod Dinosaurs in the Late Jurassic of Northern Germany

**DOI:** 10.1371/journal.pone.0158334

**Published:** 2016-07-06

**Authors:** Oliver Gerke, Oliver Wings

**Affiliations:** Department of Natural History, Landesmuseum Hannover, Willy-Brandt-Allee 5, Hanover, Germany; Trier University, GERMANY

## Abstract

Remains of theropod dinosaurs are very rare in Northern Germany because the area was repeatedly submerged by a shallow epicontinental sea during the Mesozoic. Here, 80 Late Jurassic theropod teeth are described of which the majority were collected over decades from marine carbonates in nowadays abandoned and backfilled quarries of the 19th century. Eighteen different morphotypes (A—R) could be distinguished and 3D models based on micro-CT scans of the best examples of all morphotypes are included as supplements. The teeth were identified with the assistance of discriminant function analysis and cladistic analysis based on updated datamatrices. The results show that a large variety of theropod groups were present in the Late Jurassic of northern Germany. Identified specimens comprise basal Tyrannosauroidea, as well as Allosauroidea, Megalosauroidea cf. *Marshosaurus*, Megalosauridae cf. *Torvosaurus* and probably Ceratosauria. The formerly reported presence of Dromaeosauridae in the Late Jurassic of northern Germany could not be confirmed. Some teeth of this study resemble specimens described as pertaining to Carcharodontosauria (morphotype A) and Abelisauridae (morphotype K). This interpretation is however, not supported by discriminant function analysis and cladistic analysis. Two smaller morphotypes (N and Q) differ only in some probably size-related characteristics from larger morphotypes (B and C) and could well represent juveniles of adult specimens. The similarity of the northern German theropods with groups from contemporaneous localities suggests faunal exchange via land-connections in the Late Jurassic between Germany, Portugal and North America.

## Introduction

Isolated teeth probably represent the most abundant theropod dinosaur body fossils. Even sedimentary deposits where terrestrial vertebrate fossils are very rare, such as the marine Late Jurassic carbonates in Northern Germany, yield isolated teeth. Unfortunately, theropod teeth are comparatively simple structures, so that a taxonomic assignment is often difficult, especially when more diagnostic material is absent. In such cases, statistical analysis of isolated theropod teeth can often facilitate assignment to a higher taxonomic level [1, 2], sometimes even to genus level.

Statistical identification of isolated theropod teeth has a history of 25 years. Farlow et al. [3] were the first to show the relationships between selected variables of North American theropod teeth, using reduced major axis regression (RMA). Smith et al. [2] used discriminant function analysis (DFA) and principal component analysis (PCA) for identification of isolated theropod teeth for the first time. Their method was followed in general by a series of other publications [4–7]. In 2014, Hendrickx and Mateus [1] presented the most comprehensive description of theropod teeth to date and adapted the methodology of Hwang [8, 9], where cladistic analysis is applied to assign isolated theropod teeth to known genera. Many studies that use discriminant function analysis (DFA) and principal component analysis (PCA) for the identification of isolated teeth focus on specimens from Late Cretaceous localities (e.g. [10–14]); only few concern Late Jurassic teeth [15–17].

Here, 80 theropod teeth (mainly 19th century collections) from the Late Jurassic of northern Germany are examined; most of them are previously undescribed specimens. They are compared with updated published databases using cladistic analysis and discriminant function analysis (DFA).

Among the hitherto described material, the oldest published specimen was portrayed by Graf Georg zu Münster in 1846 [18], who referred a tooth from the Kimmeridgian of Hannover (Lindener Berg) to the fossil fish “*Saurocephalus monasterii”*. Struckmann [19], not Windolf [20], as erroneously cited in Carrano et al. [21], noticed the similarity of this tooth with theropod dinosaur teeth and transferred it to “*Megalosaurus monasterii”*. Von Koenen et al. [22] mentioned (but did not describe) teeth of *Megalosaurus* from the Gigas-Beds (Late Kimmeridgian, see [23]) of Holzen. Smith [24] also listed *Megalosaurus monasterii* as present in the fauna of the Kahlberg near Echte, but gave no further description. Finally, six “velociraptorine dromaeosaurid” teeth (DFMMh/FV 382, 383, 530, 658, 707.1 and 790.5) from the Langenberg Quarry near Goslar (Harz Mountains) were described by van der Lubbe et al. [16].

### Localities and Stratigraphy

Only limited stratigraphic information is available for most specimens because many were collected from historic sites, which have long been abandoned and in part backfilled. One of the first reference of all localitities described below was reported by Roemer in 1836 [25] in his monograph on the fossil content (mainly invertebrates) of the northern German oolithic mountains.

**Fig 1:**
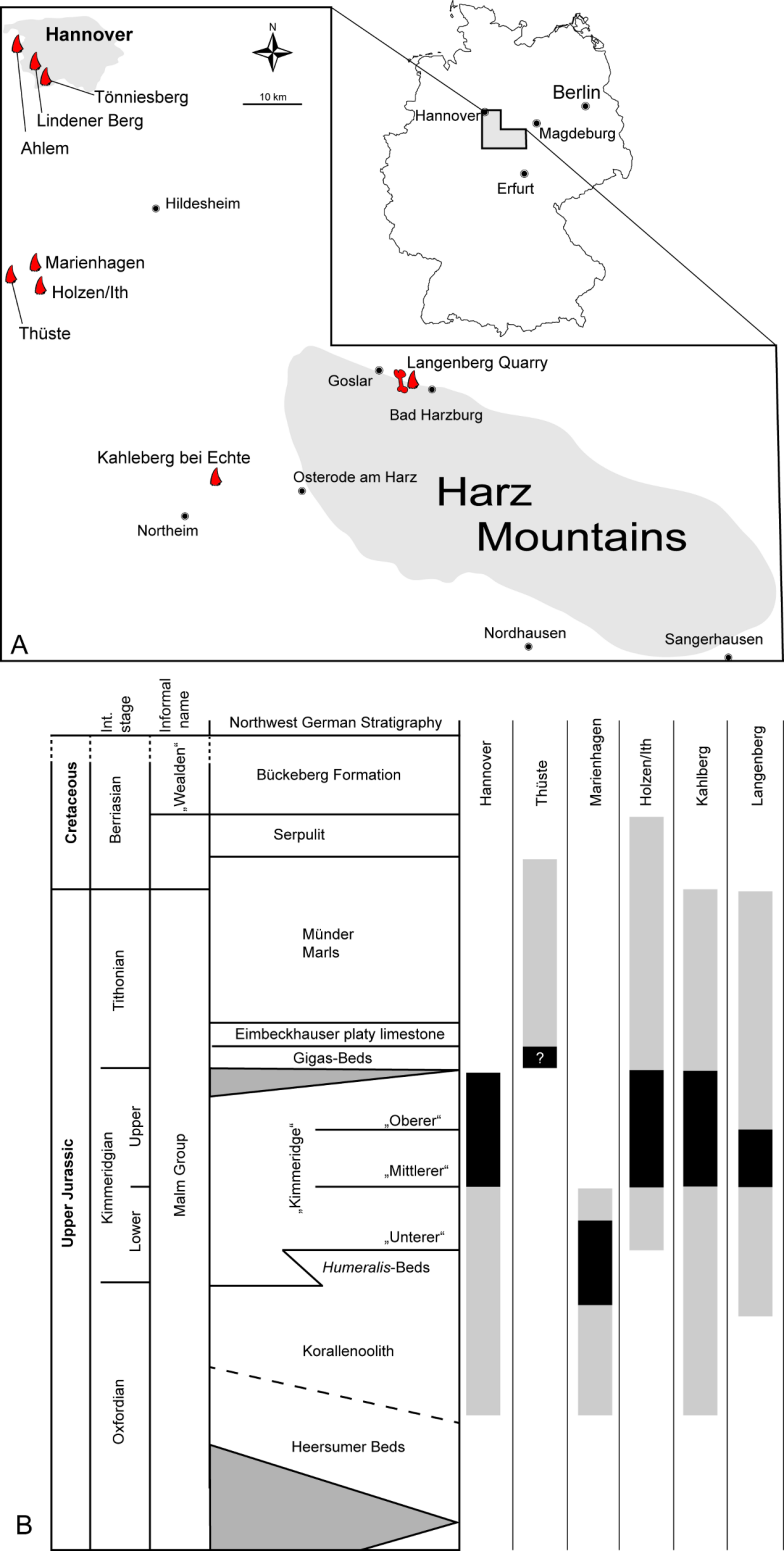
Localities and stratigraphy. **A.** Map showing the localities of the theropod teeth used in this study. **B.** Stratigraphy of the Late Jurassic in northern Germany based on Gramann et al. [29]. Bars are illustrating the stratigraphical range of the strata at the localities. Grey color: complete outcropping strata; black color: plausible stratigraphic placement of the teeth. For details and references see main text.

All sites (Fig 1) are located in the Lower Saxony Basin. During the Late Jurassic, the Lower Saxony Basin and most of northern Germany was covered by a shallow epicontinental sea [26] in which mostly marine carbonates were deposited [27]. The basin was surrounded by several large paleo-islands [26], source of the clastic components in the sediments and habitat of all terrestrial vertebrates discovered in the carbonates. Sea level fluctuations are evident from the sediments that might have facilitated faunal interchanges, perhaps strongly affecting island faunas [28]. The exact stratigraphic positions of most theropod teeth (except for the Langenberg material) are not known, but following the collection labels the majority derives from the German “Mittlerer Kimmeridge”, which is the lower part of the Late Kimmeridgian [23, 28].

### Localities in Hannover: Lindener Berg, Tönniesberg, and Ahlem

The three historic localities in Hannover (Ahlem, Lindener Berg and Tönniesberg) yielded 52 of the 80 teeth (S1 Appendix). These localities represent former quarries abandoned and backfilled between the middle of the 19th [30] and beginning of the 20th century. Today, all localities are built over, forming parts of the city of Hannover, with the exception of the quarry at the Mönckeberg in Ahlem, which has been rebuild into the Willy-Spahn-Park [31]. Many sites exposed a rather large stratigraphic section, starting with the Oxfordian “Korallenoolith” at Lindener Berg and Tönniesberg [32]. The quarrying activities focused on white fossil-rich (mainly invertebrates [19]) limestone beds in the “Mittlerer Kimmeridge”, e.g. then exposed where today the stadium Linden is located [32]. These sediments are marly and/or oolithic marine carbonates [30, 33, 34], with occasional fragmentary vertebrate remains. Besides the theropod teeth, three specimens of a sphenodontian reptile and fragmentary pterosaur remains, the vertebrate fossils comprise mainly marine groups like fishes, turtles and crocodilians [19].

### Thüste

One theropod tooth (NLMH101379) was collected in Thüste and its stratigraphic level was labeled as “Gigas-Beds”. The limestones (used as building stone) of the still active quarry are now assigned to the “Münder Mergel”- Formation (Tithonian) [35, 36]. The sediments are dominated by thick-banked oolithic limestones rich in fragments of the tubeworm *Serpula* sp. They are overlain by various thin-bedded marly layers with occasional stromatolite bioherms [35]. Beside *Serpula* sp., the ooliths are generally free of invertebrates [35, 36]. Vertebrate remains (fishes, turtles, and crocodilians) occur rarely and are very fragmentary (OG pers. obs.). No exact data were recorded for NLMH101379 and it remains unclear if the tooth was indeed collected in this quarry, as the occurrence of other vertebrate remains in these beds makes it plausible. Unfortunately, Dubbers reported in 1888 [37] a quarry located in the Marienhagener Forrest near Thüste with exposed Gigas-Beds, but provided no further description about its exact locality, lithology and fossil content. The Thüste and Marienhagen localities are only separated by a distance of five kilometers, which further complicates the stratigraphic position of NLMH101379.

### Marienhagen

The age of the marine carbonates from the Marienhagen locality spans from Oxfordian (Korallenoolith) to Early Kimmeridgian [38, 39]. Two specimens (GZG.V.010.381 and RPM.NKP.14356) were collected at this locality. GZG.V.010.381 was labeled as Oxfordian and RPM.NKP.14356 as Kimmeridgian. Von Koenen et al. [39] reported the presence of *Megalosaurus* in Oxfordian sediments of the now abandoned quarry southwest of Marienhagen, but provided no description of the material. The exposed sediments of the active quarry comprise in the lower section thick-banked, partly oolithic and dolomitic limestones that grade into marly layers alternating with thin-bedded partly fossil-rich limestone layers at top of the sequence [38]. The fossils of these thin limestone layers are dominated by invertebrates [38]. Vertebrate remains (fish and crocodile teeth, unidentifiable bone fragments; OG pers.obs.) occur rarely, are very fragmentary and often rounded, suggesting a depositional environment with high water energy.

### Holzen

Late Jurassic and Early Cretaceous sediments can be found in the area around the asphalt-rich limestones near Holzen/Ith, which were quarried (now abandoned) for more than 120 years [40]. The Kimmeridgian sequence is dominated by massive limestone beds up to several meters thick alternating with thin-bedded mudstones and marl layers [40].

Five theropod teeth (GZG.V.010.333, GZG.V.010.389, GZG.V.010.329, GZG.V.010.332 and GZG.V.010.331) from Holzen are labeled “Oxfordium?” and one (MB.R.2800) as “Purbeck/Serpulit”. Von Koenen et al. [22] mentioned teeth of *Megalosaurus* and that they derive from the Gigas-Beds. The exposed Gigas-Beds in the Holzen area are now interpreted as Late Kimmeridgian age [23].

#### Kahlberg near Echte

Several quarries were active in the 19^th^ century at the “Kahleberg” (nowadays: Kahlberg) [24, 41]. The often dolomitic and oolithic limestones in the area were rich in invertebrate fossils such as bivalves and gastropods, but also contained vertebrate fossils. Invertebrate fossils indicate a Late Jurassic age, ranging from Oxfordian to Tithonian [41]. Beside early reports of undetermined bone fragments, possibly of turtles [41], remains of crocodyliforms, fishes, and *Megalosaurus monasterii* were found [24]. The remains of *Megalosaurus monasterii* derive from the marly, oolithic *Lepidotus*-Beds, which were dated with invertebrates as Late Kimmeridgian [24]. Following the collection label (“*Megalosaurus monasterii*, Late Kimmeridgian”), these remains probably represent morphotype K (GZG.V.010.334) of this study.

### Langenberg Quarry

The Langenberg Quarry is a classic and well-studied outcrop exposing excellent sections of shallow marine strata [42–44]. It is situated near the town of Goslar, Lower Saxony, northern Germany. These beds comprise impure carbonates that grade into marls and are tilted to a near vertical, slightly overturned position. The sediments in the quarry are well dated by biostratigraphy (ostracode zones and rare ammonites) and range from Late Oxfordian to Late Kimmeridgian in age [42–44]. After the stratigraphic subdivision of Fischer (42], most terrestrial vertebrate remains (including the sauropod dinosaur *Europasaurus* and several theropod teeth described herein) were found in bed 83, not in bed 93 as erroneously stated in recent publications [45–47]. This bed, a light grey-greenish marly limestone, has been assigned to the “Mittleres Kimmeridge”, a northwest-German equivalent to the lower part of the Late Kimmeridgian of the international chronostratigraphic time scale [23, 28].

The Langenberg Quarry is the only locality where exquisitely three-dimensionally preserved material of the dwarfed sauropod dinosaur *Europasaurus holgeri* has been found [45–47], which represents by far the most abundant material of terrestrial vertebrates in Late Jurassic strata of Northern Germany. Beds 83 and 73 also have produced exceptional material of non-dinosaurian vertebrates [48]. This includes the three-dimensionally preserved articulated skeleton of a dsungaripterid pterosaur [49], teeth and skeletons of the small non-marine atoposaurid crocodilian *Theriosuchus* [50, 51] and the associated partial skeleton of a paramacellodid lizard [52]. Diverse turtle material (including several skulls) comprises taxa including cf. *Thalassemys* and *Plesiochelys* [53]. In addition to common reptilian teeth (OW, pers. obs.), microvertebrate remains from the Langenberg Quarry include a diverse fish fauna represented mainly by isolated teeth of marine chondrichthyans and osteichthyans [54–56], and the first Jurassic mammal teeth from Germany [57, 58].

Throughout the sequence, changes in water depth and brackish influences are apparent due to sediment composition and invertebrate faunal content, but there is no evidence of subaerial exposure [43, 44]. Despite the large number of bones and teeth known from *Europasaurus*, the general distribution of vertebrate remains in bed 83 is scarce. Skeletal remains were accumulated in certain areas, probably lenses or channels. The bone-bearing sections of bed 83 are usually 30–50 cm thick and also contain a large number of well-rounded micritic intraclasts in all bone-rich areas.

### Institutional Abbreviations

**DFMMh/FV:** Dinosaurier-Freilichtmuseum Münchehagen/Verein zur Förderung der Niedersächsischen Paläontologie (e.V.), Germany; **NLMH**: Niedersächsisches Landesmuseum Hannover, Germany; **GZG**: Geowissenschaftliches Zentrum der Universität Göttingen, Museum, Germany; **MB**: Museum für Naturkunde, Berlin, Germany; **GSUB**: Geowissenschaftliche Sammlung der Universität Bremen, Germany; **RPM**: Roemer- Pelizaeus Museum Hildesheim, Germany; **ML**: Museu da Lourinhã, Lourinhã, Portugal

### Morphometric Abbreviations

**AL**: apical length (in mm); **CA:** crown angle, using the formula of the law of cosine [2]; **CAA:** crown apical angle; **CBL**: crown base length (in mm); **CBR**: crown base ratio (CBW/CBL); **CBW**: crown base width (in mm); **CDA:** crown distal angle; **CH**: crown height (in mm); **CHR**: crown height ratio (CH/CBL); **DA**: distal apical-crown serration count per 5 mm; **DC**: distal mid-crown serration count per 5 mm; **DB**: distal base-crown serration count per 5 mm; **MA:** mesial apical-crown serration count per 5 mm; **MC:** mesial mid-crown serration count per 5 mm; **MB:** mesial base-crown serration count per 5 mm; **DSDI**: denticle size difference index, MC divided by DC

## Materials and Methods

The material comprises 80 theropod teeth from the Late Jurassic of Lower Saxony/Northern Germany. All specimens were collected during the 19^th^ century, with the exception of the material from the Langenberg locality (S1 Appendix). Permissions for fieldwork at the Langenberg/Goslar locality were granted by quarry owner Fabian von Pupka. For the historic sites no data were recorded if the teeth were found on public or private land. Lower Saxony then had no law to protect paleontological sites and their fossils. All specimens described in this study (S1 Appendix) are permanently housed in the publicly accessible repositories of the Geoscience Centre University of Göttingen (GZG.V.010.318–323, 325–335, 344, 345, 369–374, 377–381, 389, 392, 399), Geoscience Collection University Bremen (GSUBV4022, 4023), Museum of Natural History Berlin (MB.R.2800), Lower Saxony State Museum Hannover (NLMH16416a, 16480, 101375a, 101375b, 101376a –e, 101377, 101378a –c, 101379, 101380a, 101380b, 101380e, 105652–105654, 106235a -c), Roemer—Pelizaeus Museum Hildesheim (RPM14356–14359) and the Dinosaurpark Münchehagen/Association for Support of the Lower Saxonian Paleontology e.V. (DFMMh/FV 530, 382, 383, 658, 707.1, 790.5.,1202–1206, CL360L and CL360S).

All specimens probably represent shed teeth, except for GZG.V.010.335, which preserves an almost complete root.

Morphometric measurements (S1 Appendix) were taken with standard calipers to the nearest 1/10^th^ mm. Visual examination was carried out with a Zeiss Stemi SV 11 binocular and photos taken using a Canon EOS 60D with Sigma 50 mm macro lens.

To compile the 3D –data the selected teeth (S11–S28 Appendices) were scanned with the Micro-CT v|tome|x s 240 scanner (GE Sensing and Inspection Technologies Phoenix|x-ray, software: datos|x–reconstruction 1.5.0.22 64 bit) at the Steinmann-Institute of the University Bonn/Germany, using the “resolution 2” function to artificially double the resolution, and, if needed, the“ring artifact reduction”, a beam hardening correction between 6–7 and automatic geometry calibration. The scans were automatically segmented with the software VGStudio Max 2.0.1 64 bit von Volume Graphics GmbH -VGL version 4.0.0 (32958) with manual object calibration and super precise surface extraction. Extraction and point reduction were both set to precise.

**Fig 2:**
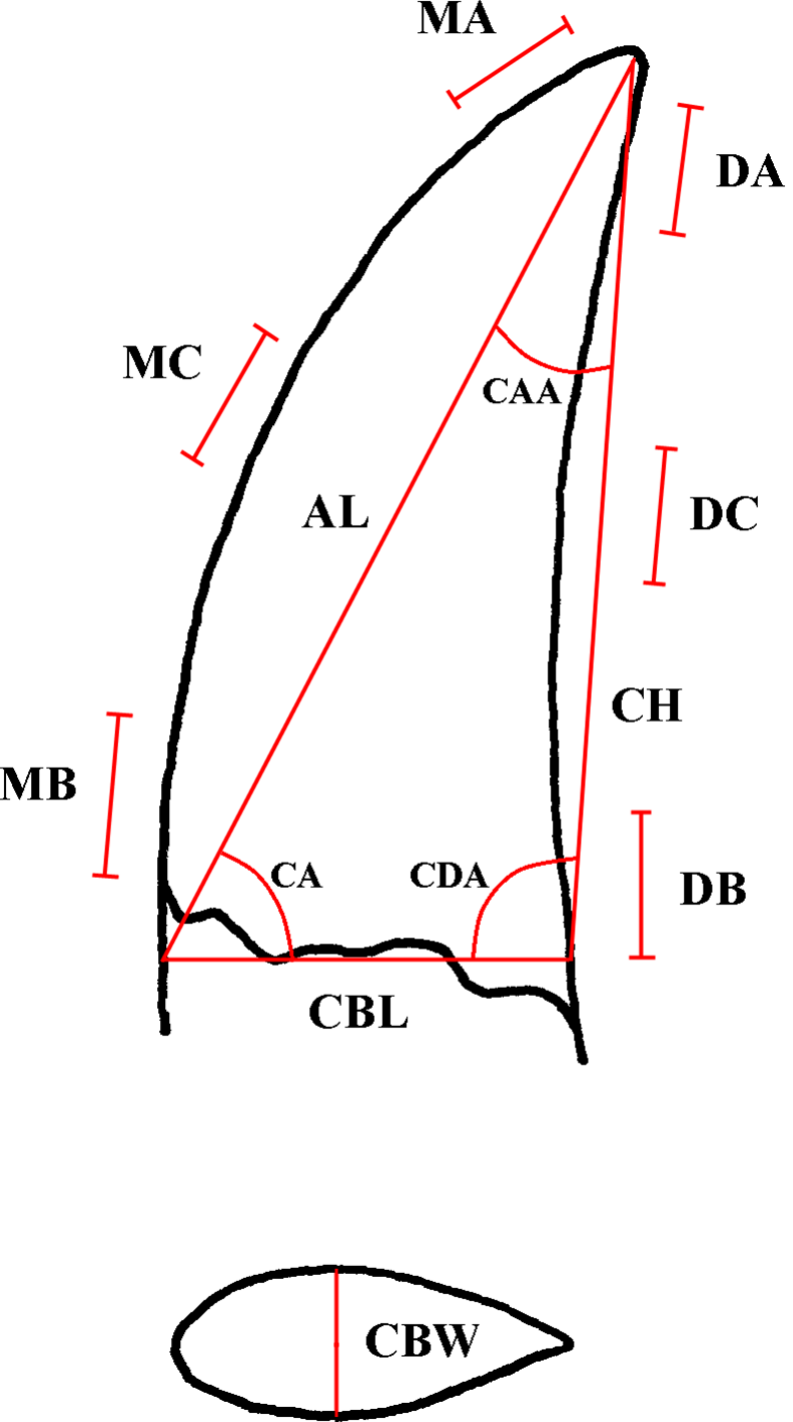
Variables for the DFA. Variables used for the discriminant function analysis (DFA) (after [2, 16]).

Morphometric abbreviation terms (Fig 2) follow Smith et al. [2]; directional nomenclature Smith and Dodson [59] and the descriptive terminology Hendricks and Mateus [1]. Different descriptive terms are in use for identical structures of theropod teeth, e.g., the term “transversal undulation” [1], which is synonymous with “enamel wrinkles” [60]. Hendrickx and Mateus [1] used “marginal undulations” for short wrinkles adjacent to the mesial and/or distal carinae that are often referred as “transversal undulations” [60]. Hendricks and Mateus [1] provide a valuable overview, including figures, of descriptive terms on theropod teeth.

### Methodology of cladistic analysis with comments on Hendrickx and Mateus [1]

For the cladistic analysis, a modified version of the supermatrix (S2 Appendix) of Hendricks and Mateus [1, 61] is used that comprises 60 taxa with 1972 characters, 141 of these are tooth-based. Applicable for the isolated teeth presented in this study are 108 characters. The analysis was executed with TNT 1.1 [62] using the “New Technology Search” algorithm with selected “Sect. Search”, Ratchet, Drift and “Tree fusing” with default parameters. Under the “Driven Search” option, consensus trees were stabilized twice with a factor of 75. The consistency and retention indices (S4 Appendix) were obtained with the “stats.run” script provided by Goloboff et al. [62]. All 80 examined teeth in this study were *a priori* assigned to different morphotypes based on their characters and combination of features e.g. the presence of flutes in morphotype M; the strongly recurved and flattened tooth of morphotype N; high DSDI (>1.2), recurvature of the crown and outline of the basal cross-section in morphotype E, F and G; relatively straight distal margin, strongly twisted mesial carinae that terminates at the cervix in morphotype K. The morphotypes were coded (S1 Appendix) as lateral, except for morphotype L, M and eventually H which probably represent mesialmost teeth (premaxillary and most mesial dentary teeth [1]). Mesialmost teeth could be distinguished from lateral teeth in often possessing a subcircular to elliptical, J-shaped, U-shaped and D-shaped basal cross-section [1]. In certain taxa they additionally show ornamentations such as flutes (e.g. *Ceratosaurus* [1]), basal striations (*Proceratosaurus* [1]) or the carinae are devoid of denticles (e.g. *Compsognathus* [1]).

**Fig 3:**
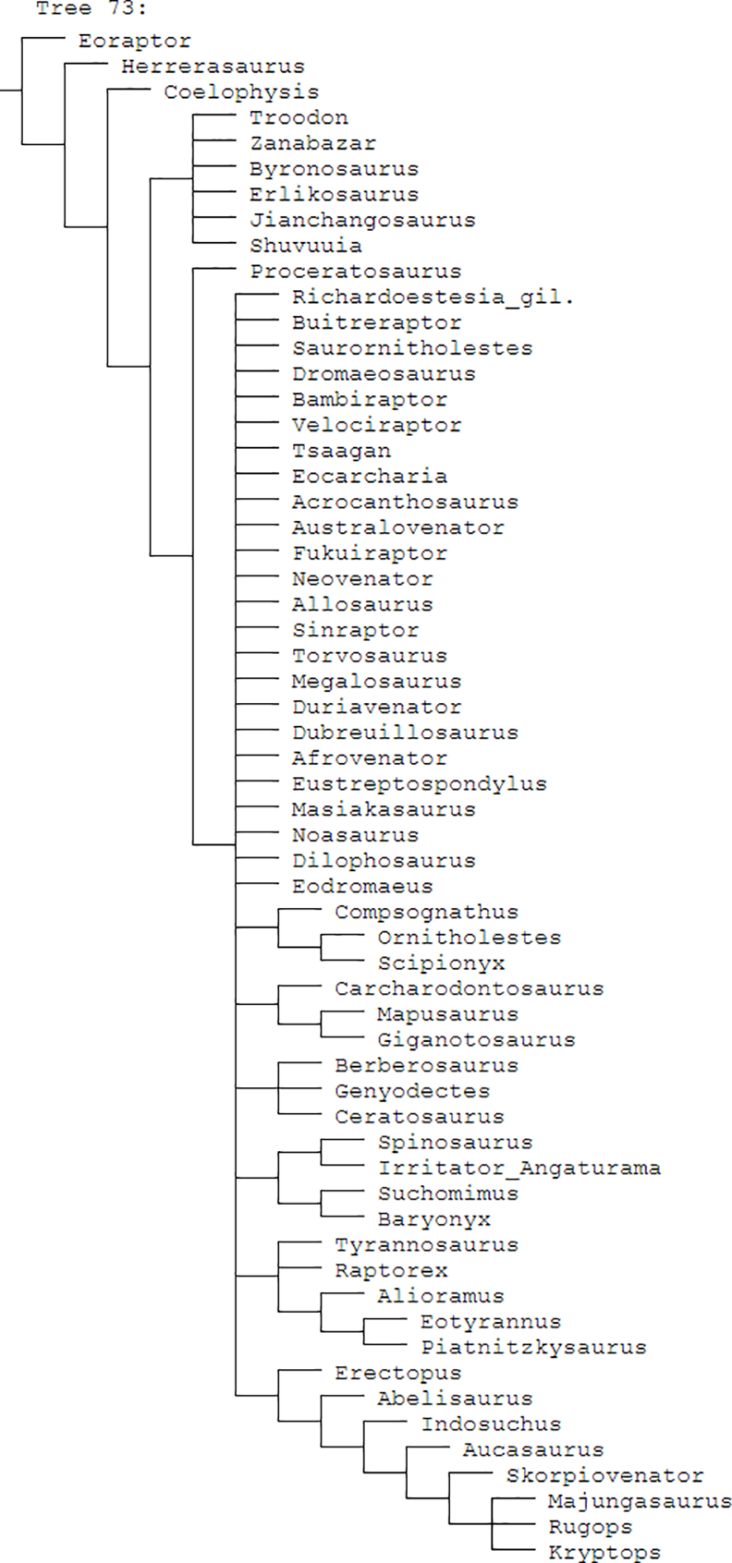
Cladogram of the updated datamatrix with dentition-based characters only. Strict consensus cladogram of 73 most parsimonious trees recovered by TNT for the modified (S5 Appendix) dentition-based datamatrix (141 characters) of Hendrickx and Mateus [61]. Tree length = 665, CI = 0.261 and RI = 0.354.

**Fig 4:**
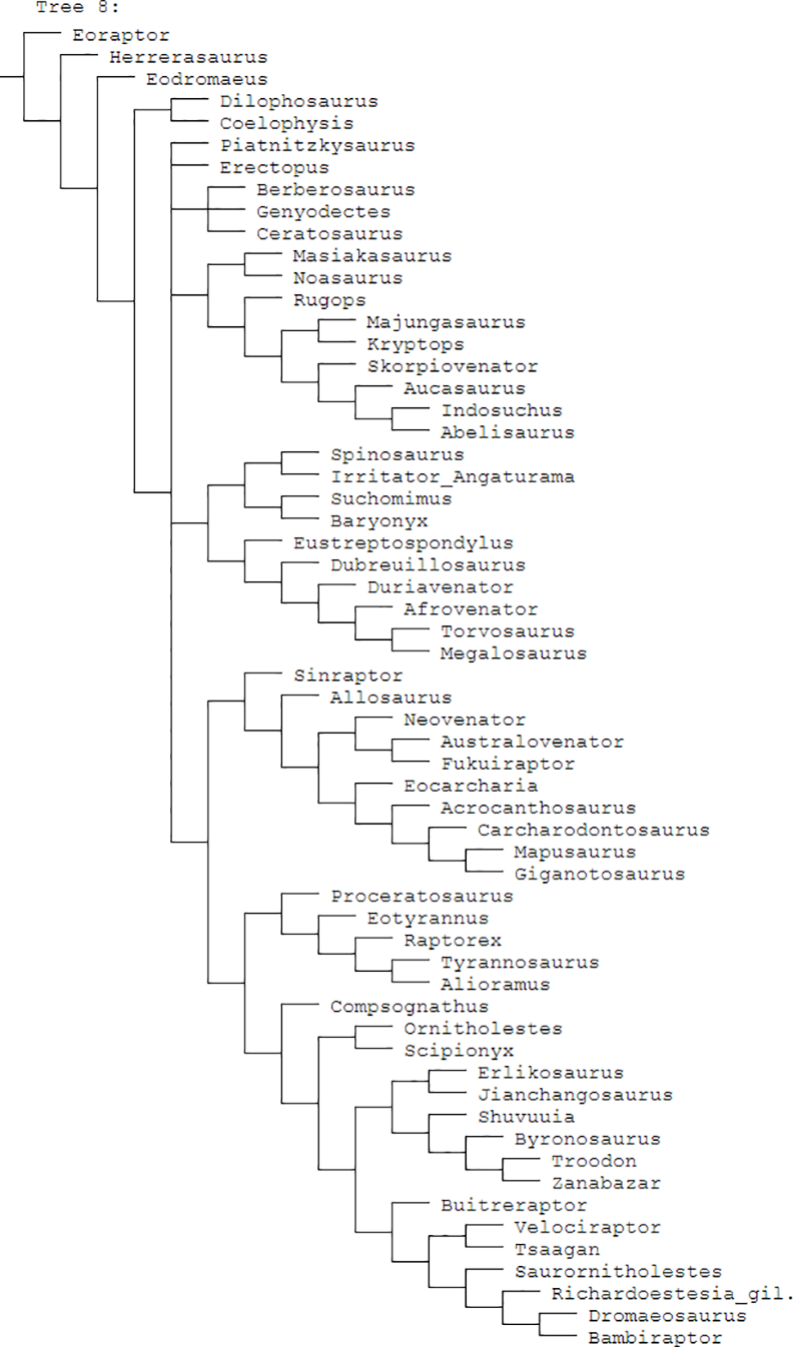
Cladogram updated supermatrix. Strict consensus cladogram of eight most parsimonious trees recovered by TNT for the datamatrix (1972 characters) used in this study (S2 Appendix) that is based on the supermatrix of Hendrickx and Mateus [61]. Tree length = 3571, CI = 0.547 and RI = 0.604.

The datamatrix and supermatrix presented by Hendrickx and Mateus [1, 61] were updated as several errors were discovered during this study. For example, the codings of *Richardoestesia* differ considerably between their supermatrix and datamatrix. See supplement (S3 Appendix) for a list of changed codings. References for these updates are mainly based on the datasets and descriptions provided by Hendrickx and Mateus [1, 61, 63] and Hendrickx et al. [64]. Rerunning their cladistic analysis (without the morphotypes described below) using the updated datamatrix (S5 Appendix) including the changed codings (S3 Appendix), comprising only the 141 dentition-based characters, results in an unresolved strict consensus of 73 most parsimonious trees (CI = 0.261, RI = 0.354) (Fig 3). This polytomy is difficult to interpret, so only the use of the updated supermatrix provided here ([61]; S2 Appendix, Fig 4) could be recommended.

### Methodology of discriminant function analysis with comments on Hendrickx et al. [64]

The discriminant function analysis (DFA) was performed with the free statistics software R [65] using the R GUI Deducer [66], based on the JGR console [67] including the package “DeducerPlugInScaling” [68]. It uses linear discriminant analysis (LDA) for more than two groups of the “MASS” package [69] for the DFA and the predict function for classifying the new cases. This technique is analogous to canonical variate analysis (CVA) [70]. Linear discriminant analysis (LDA) is a statistical technique for finding the linear combination of features that best separate observations into different classes [71]. For morphotypes with rather unsteady results, a higher taxonomic level DFA was conducted. Principal component analysis (PCA) was carried out additionally to check if separation of the morphotypes is justified. PCA is a statistical procedure reducing a multivariate dataset down to usually two dimensions, which preserve as much variance as possible [70].

The graphics (S6 Appendix) were created with the R package “BiplotGUI” [72] and “directlabels” [73].

The dataset (S6 Appendix) for the DFA comprises tooth measurements previously published by Smith et al. [2] and Smith and Lamanna [74] for the taxa *Liliensternus*, *Ceratosaurus*, *Masiakasaurus*, *Majungasaurus*, *Duriavenator*, *Baryonyx*, *Suchomimus*, *Allosaurus*, *Acrocanthosaurus*, *Carcharodontosaurus*, *Daspletosaurus*, *Tyrannosaurus*, *Deinonychus*, *Dromaeosaurus*, *Velociraptor*, *Troodon*, *Zanabazar*, and *Marshosaurus*, with recalculated ratio variables CBR, CHR and CA, as during an early stage of this study some incongruences in the dataset were observed. Measurements provided by Rauhut et al. [75] for *Proceratosaurus* were also included, with missing data (CBW and AL) measured on photographs that were scaled for CBL and CH. The dataset of this study was completed with taxa Hendrickx et al. [64] published for *Coelophysis*, *Berberosaurus*, *Genyodectes*, *Erectopus*, *Piatnitzkysaurus*, *Afrovenator*, *Dubreuillosaurus*, *Torvosaurus*, an “unpublished megaraptoran” (juvenile *Megaraptor*, [76]), *Neovenator*, *Giganotosaurus*, *Raptorex*, and *Alioramus*. Mesialmost teeth (premaxillary and anterior dentary teeth [1]) of *Ceratosaurus*, *Dubreillosaurus*, *Proceratosaurus* and *Torvosaurus* are treated as “separate taxa” in this study to account for differences compared to lateral teeth. This was done only for taxa with similar stratigraphical range as the morpthotypes described here. The separation in mesialmost and lateral teeth results in a better resolution (reclassification rate) in these taxa (S6 Appendix). As cutoff for assigning dentary teeth of a taxon to mesialmost the corresponding number of the premaxillary teeth was chosen, e.g. *Proceratosaurus* has four premaxillary teeth [1, 61], so dentary one to four were regarded as mesialmost. Most theropod taxa included in the updated datamatrix of Hendrickx and Mateus have four premaxillary teeth [1, 61], with the exception of, e.g. Spinosauridae (six to seven [1, 61]), *Ceratosaurus* (three [1, 61]) and *Allosaurus* (five [1, 61]). *Allosaurus* was excluded from this procedure as the discrimination between the included lateral and mesialmost teeth is difficult when only morphometric data (e.g. CBR and CHR) are taken into account (see S6 Appendix). Applying this method *on Allosaurus* teeth results (S6 Appendix) in a poor resolution of its lateral teeth (reclassification rate 28.57%). The resolution becomes better (mesialmost 82.35% and lateral 80%) when mx1 and d6 of UMNHVP9218 ([2, 74] S6 Appendix) were transferred to mesialmost. These two teeth are very similar to the mesialmost regarding morphometric data, e.g. in possessing a more subcircular to eliptical basal cross-section (CBR of 0.75 and 0.70).

For the DFA the variables were log transformed to obtain a better normal distribution [11] using the formula log(x+1) to account for values of 0, e.g. to include MC for the taxon *Zanabazar*. The variables used for the LDA comprise log(x+1) transformed CBW, CH, AL, CHR, MC and DC. The LDA was executed with prior probability set that the groups were treated as equal under the “options” menu; the other settings were retained with default options. To obtain the classification, posterior probability and statistics the corresponding boxes were ticked under the “Export” and “Show” menu. The values returned by the LDA indicate the probability (in percent) that a tooth is correctly assigned to a certain group. In teeth where the posterior probabilities are rather weak, the second rank classification is also indicated. For tooth crowns where MC is not preserved, log(x+1) transformed CBW, CH, AL, CHR, CBR and DC were used. The reclassification rates are 86.87% for the dataset with MC and DC and 82.09% when only DC was taken into account. The variable selection of the two LDA represents the best possible solution for the used dataset regarding reclassification rate. The differences in the reclassification rates indicate that variable MC is crucial in discrimination between theropod teeth based on morphometric data.

Hendrickx et al. [64] used a different method to measure the variables CBL and Al (see their Fig 1C) than Smith et al. [2]. We compared the measurements of CBL and Al provided by Hendrickx et al. [64] with photos that were scaled after CH. Any observed differences were corrected to reach compatibility between the datasets of Smith et al. [2] and Hendrickx et al. [64]. The differences in the measurement method of Hendrickx et al. [64] results in shortening of variable AL and lengthening of CBL; consequently it has also influence on CA, CBR and CHR. Hendrickx et al. [64] specifies, e.g. for the sixth left dentary tooth of *Dubreillosaurus* (MNHN 1998–13) a CA of 70.48°. Measuring CA of this tooth (Fig 2 of [64]) directly on a photograph (using, e.g. MeazureTM 2.0, [77]) or calculating it with the corrected measurements following the protocol of Smith et al. [2] results in a CA of 59.11°. We further tested this with a LDA on a reduced dataset (without the morphotypes described in our study, S7 Appendix) of Smith and Lamanna [74]. The overlapping taxa of Hendrickx et al. [64] were extracted and classified with the reduced dataset of Smith and Lamanna [74]. The variables comprise log(x+1) transformed CBL, CBW, CH, AL, CA, DC, CBR, CHR. Only 53.78% of the taxa extracted from Hendrickx et al. [64] were correct classified to their original group membership. The jackknifed (leave-one-out cross-validation) reclassification rate of the reduced dataset of Smith and Lamanna is 83.9%.

When expanding an existing database, all measurements should be obtained with the same method. Using the mesial extent of the enamel as the basis of a measurement is problematic as it varies within certain taxa and seems to have no taxonomic value [78], with the possible exception of megalosaurid teeth [64]. The mesial sides of isolated teeth are more affected by abrasion and erosion during transportation and often not well preserved (OG pers. obs.). However, we acknowledge the difficulties in measuring variable AL, but this problem could in part be avoided by remembering the measuring point of AL at the base of the crown (any surface structures, cracks etc.).

Hendrickx et al. [64] stated that ratio variables should be avoided in DFA because they weight the dependent variables. This is true, but only when the ratio variable is included together with the dependent variables. The usefulness of ratio variables is dependent on the composition of the groups included in the dataset and variables that are used in the study. Sometimes, ratio variables give, like in our sample, better results than their dependent variables. For example, a replacement of CHR with CBL causes a small drop (86.27%) of the reclassification rate (86.87%) in our used dataset. The vast decrease of the reclassification rate Hendrickx et al. [64] encountered when comparing their “large ziphodont teeth” with or without ratio variables could not be retraced when rerunning their analysis. We assume that their study is highly obscured by missing data. Hendrickx et al. [64] used the software Past [79]. Past accepts missing data, but only with column average substitution [80] of the variables. Analyses with large amounts of missing data could not always be avoided in paleontological data, but they are difficult to interpret and such results should be treated with caution. Occasional occurrences of missing values can be supplemented with the group-means of the variable. However, imputing large amounts of missing data may result in underestimating the variance [81].

With a reclassification rate of 86.87% for the DFA, our values are slightly below the recommended minimum hit ratio of 90% [70]. DFA on theropod teeth with higher hit ratios were often implemented with SPSS [82], like in the studies of Smith et al. [2] and Serrano-Martínez et al. [7]. They apparently used the option “Separate-groups” in “Use Covariance Matrix”in the classification menu to obtain the high reclassification rate (> 90%) of their datasets. This option gives sometimes results similar to quadratic discriminant analysis (QDA), depending on the number of groups and variables [83]. Quadratic discriminant analysis (QDA) has advantages of an increased flexibility compared to LDA, however, it has disadvantages in potential overfitting the data and classifying new observations [84]. Other statistical software, such as R GUI Deducer [66], Past [79] and JMP (LDA) [85], use the “pooled within-group covariance matrix” for classifying. Changing in SPSS, under the classification menu, the option “Use Covariance Matrix”to “Within-groups” results in a reclassification rate comparable to that obtained with R GUI Deducer, Past or JMP (LDA).

## Description, Results and Discussion of the Morphotypes

### Morphotype A

#### Material

**Fig 5:**
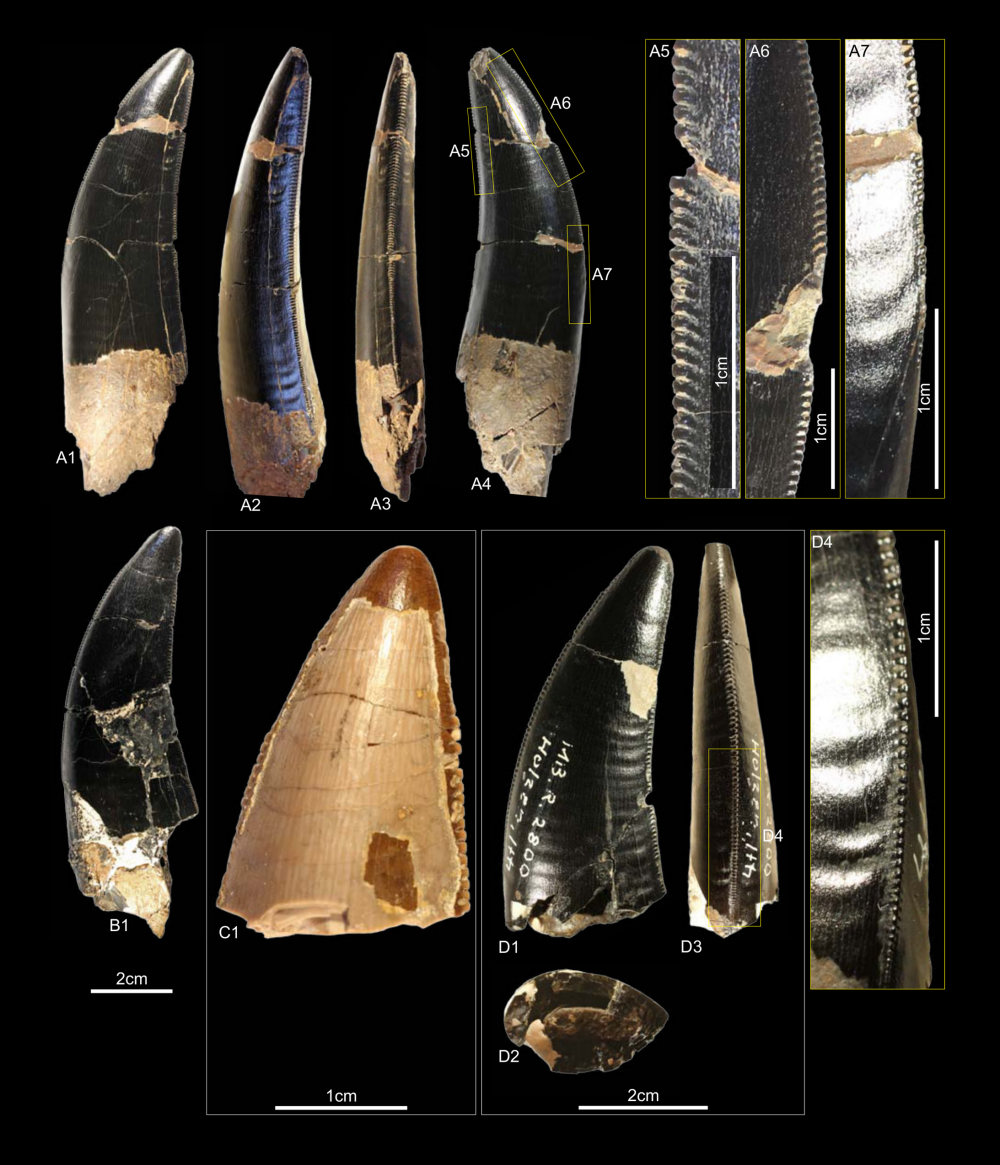
**Morphotype A:** A, **GZG.V.010.333**, A1 in labial, A2 oblique distal, A3 distal and A4 lingual view, A5 details distal and A6–A7 mesial carinae; B1, **GZG.V.010.389** in labial view; C1, **NLMH101379** in lateral view; D, **MB.R.2800**, D1 in labial, D2 basal and D3 mesial view, D4 marginal undulations mesial carina in mesial view.

GZG.V.010.333, GZG.V.010.389, MB.R.2800, NLMH101379 (Fig 5)

Morphotype A comprises three teeth from Holzen (MB.R.2800, labeled as “Serpulit/Purbeck”) and one from the Gigas-Beds of Thüste (NLMH101379).

#### Description

The specimens of morphotype A are nearly complete, except for NLMH101379, where only the tip is preserved. With a CH of 56.5–77mm, they represent the largest teeth in our study. The crowns are moderately recurved with a CA around 63–64°. The basal cross section is lanceolate with a CBR of 0.5–0.59. The distal carinae run along the midline in distal view, only in GZG.V.010.389 it is displaced labially, and terminates well below the cervix. The non-twisted mesial carinae are centrally positioned on the crowns in mesial view and extend also down to the cervix, except for GZG.V.010.333 where it terminates slightly above. The enamel surface is well preserved and has a braided texture. The denticles are very coarse, DC and MC varies from 6–7 denticles per 5 mm with a DSDI of 0.85 to 1.17. The apical mesial denticles are quadrangular in lateral view and slightly inclined apically. Distal denticles are rather horizontal rectangular, and also slightly inclined apically at midcrown. In GZG.V.010.333 and GZG.V.010.389, this feature varies as some denticles are more perpendicular to the mesial margin. Well-developed interdenticular sulci run diagonally towards the base at midcrown of the distal carinae and become shorter at the basalmost denticles. Except for GZG.V.010.333, the basal denticles are devoid of this feature. The interdenticular sulci along the mesial carinae are less pronounced than that of the distal. They are not preserved due to wear in GZG.V.010.389 and absent at the apex in MB.R.2800. Large, numerous, transversal undulations run mesio-distally on the labial and lingual surface of the crown (to a lesser extent in GZG.V.010.389). The marginal undulations are short, shallow, and mesio-distally oriented. They are present on the mesial side of the teeth, in GZG.V.010.333 also on the distal.

#### Results and Discussion

Following the collection labels of the specimens, the stratigraphic range of morphotype A spans from the? Oxfordian to the lower Berriasian. No exact localities and stratigraphic positions are recorded for the material. GZG.V.010.333, GZG.V.010.389 and MB.R.2800 were found at Holzen and could represent the *Megalosaurus* teeth mentioned by von Koenen et al [22]. These beds are now regarded as Late Kimmeridgian age [23]. The cladistic analysis assigns (S4 Appendix) morphotype A to *Torvosaurus*, agreeing in part with DFA results (S1 Appendix; GZG.V.010.333, *Torvosaurus* mesialmost, 68.8%; GZG.V.010.389, *Torvosaurus*, 84.9%; MB.R.2800, *Afrovenator*, 33.29%). Morphotype A bears very coarse denticles on the mesial and distal carinae (MC and DC, 6–7). A similar denticle count is seen only in larger teeth of *Tyrannosaurus*, *Torvosaurus* and *Carcharodontosaurus* [1, 2].

Another distinctive feature of morphotype A is that the mesial carinae reach the cervix. This distinguishes morphotype A from *Torvosaurus* teeth [63] where the mesial carinae terminate well above the cervix. In an isolated *Torvosaurus* tooth from Portugal (ML857; [64]) the mesial denticles terminate in the basal one fifth. If there is further variation of this character could not be verified based on the rather limited material known from this taxon [63, 86–88]. Janensch [89, 90] described several teeth as *Megalosaurus*(?) *ingens* from the Late Jurassic (late Tithonian) of Tanzania with similar denticle count and serrated mesial carinae that reach the cervix. Rauhut [91] used this combination of features to assign these teeth tentatively to Carcharodontosauridae and considered it possible that they represent the teeth of *Veterupristisaurus milneri*, the oldest member of this clade (Kimmeridgian to earliest Tithonian). By comparison, the earliest European occurrences of Carcharodontosauria (sensu Benson et al. [92]) are *Neovenator* from the Barremian of England [60] and Concavenator of Spain [93]. Morphotype A also shares with the Tendaguru material the quadrangular-shaped mesial denticles, well-developed interdenticular sulci at the distal carinae and well-developed marginal undulations [91]. Possible additional congruence must await further description of the Tendaguru material (O.W.M. Rauhut in prep.). Following the results of the statistical analysis morphotype A is regarded here as a member of the clade Megalosauridae, but this assignment is only provisionally given the similarities to the Tendaguru material.

### Morphotype B

#### Material

**Fig 6:**
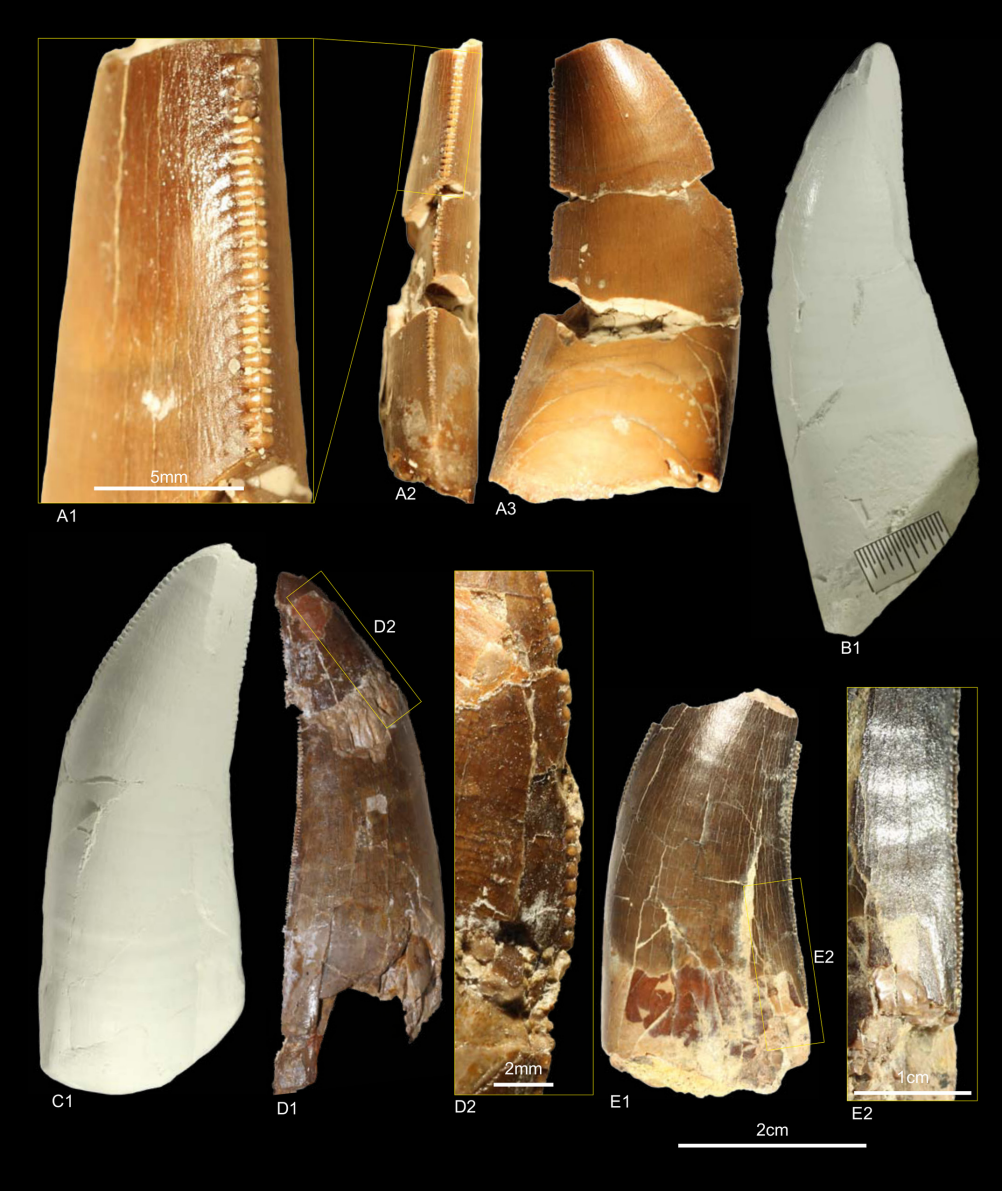
**Morphotype B:** A, **GZG.V.010.381**, A1 details distal carina, A2 distal and A3 lingual view; B1, **DFMMh/FV1205** in labial view; C1, **DFMMh/FV1206** in lateral view; D, **RPM.NKP14356**, D1 lateral view and D2 details mesial carina; E, **NLMH101375b** E1 in lateral view and E2 details distal carina.

DFMMh/FV1205, DFMMh/FV1206, GZG.V.010.381, NLMH101375b, cf. RPM.NKP.14356 (Fig 6)

Morphotype B comprises two teeth from the Langenberg Quarry near Goslar (DFMMh/FV1205, DFMMh/FV1206; Kimmeridgian); two teeth from Marienhagen (GZG.V.010.381, Oxfordian; RPM.NKP.14356, Kimmeridgian) and one tooth (NLMH101375b) from Ahlem in Hannover (Kimmeridgian).

#### Description

Morphotype B comprises rather poorly preserved teeth: two casts (DFMMh/FV1205, DFMMh/FV1206); one poorly preserved tooth with a missing tip (GZG.V.010.381); a lower two thirds of a tooth with partial root (NLMH101375b); and a complete tooth (RPM.NKP.14356) where only the labial side is visible as the lingual side is still enclosed in matrix. Morphotype B shows some similarities with morphotype A, such as the distal carinae that terminate well below the cervix; comparatively coarse denticulation; lanceolate outline of basal cross-section; existence of marginal undulations; and well developed interdenticular sulci. However, some differences exist that allow us to separate both morphotypes: the mesial carinae terminate well above the cervix; apical mesial denticles are rather vertical rectangular and perpendicular-oriented to the mesial margin. The mesial denticles are also more chisel-shaped, with narrower interdenticular space and a MC of 7 to 9 denticles. The midcrown denticles (DC 8 to 10) of the distal carinae are perpendicular to distal margin. CH ranges from 41.3–57 and CBR from 0.48–0.53.

#### Results and Discussion

The cladistic analysis recovers (S4 Appendix) morphotype B close to *Torvosaurus* and *Megalosaurus*. The DFA assigns (S1 Appendix) four teeth (DFMMh/FV1205, DFMMh/FV1206, GZG.V.010.381 and RPM.NKP.14356) to the megalosaurids *Afrovenator* and *Duriavenator*. NLMH101375b is classified as *Majungasaurus* (39.91%), or possibly *Duriavenator (26*.*16%)*, but as this tooth is very incomplete, the results should be viewed with caution. Comparison of morphotype B with the megalosaurid teeth described by Hendrickx et al. [64] and Benson [94] shows the similarities, e.g. the rather narrow interdenticular space; the distal carinae terminate well below the cervix and the mesial carinae never reach the cervix. The only difference between morphotype B and teeth described as *Torvosaurus* [1, 61, 63] are the apical mesial denticles, which seem to be vertical rectangular in morphotype B. The tooth crowns of morphotype B are regarded here as Megalosauridae cf. *Torvosaurus* sp. This interpretation appears probable given the presence of tooth and bone material described as *Torvosaurus* from the Late Jurassic of Portugal [63, 64], demonstrating the similarities with the German theropod fauna. However, this assignment is only tentatively because of the rather incomplete specimens.

### Morphotype C

#### Material

**Fig 7:**
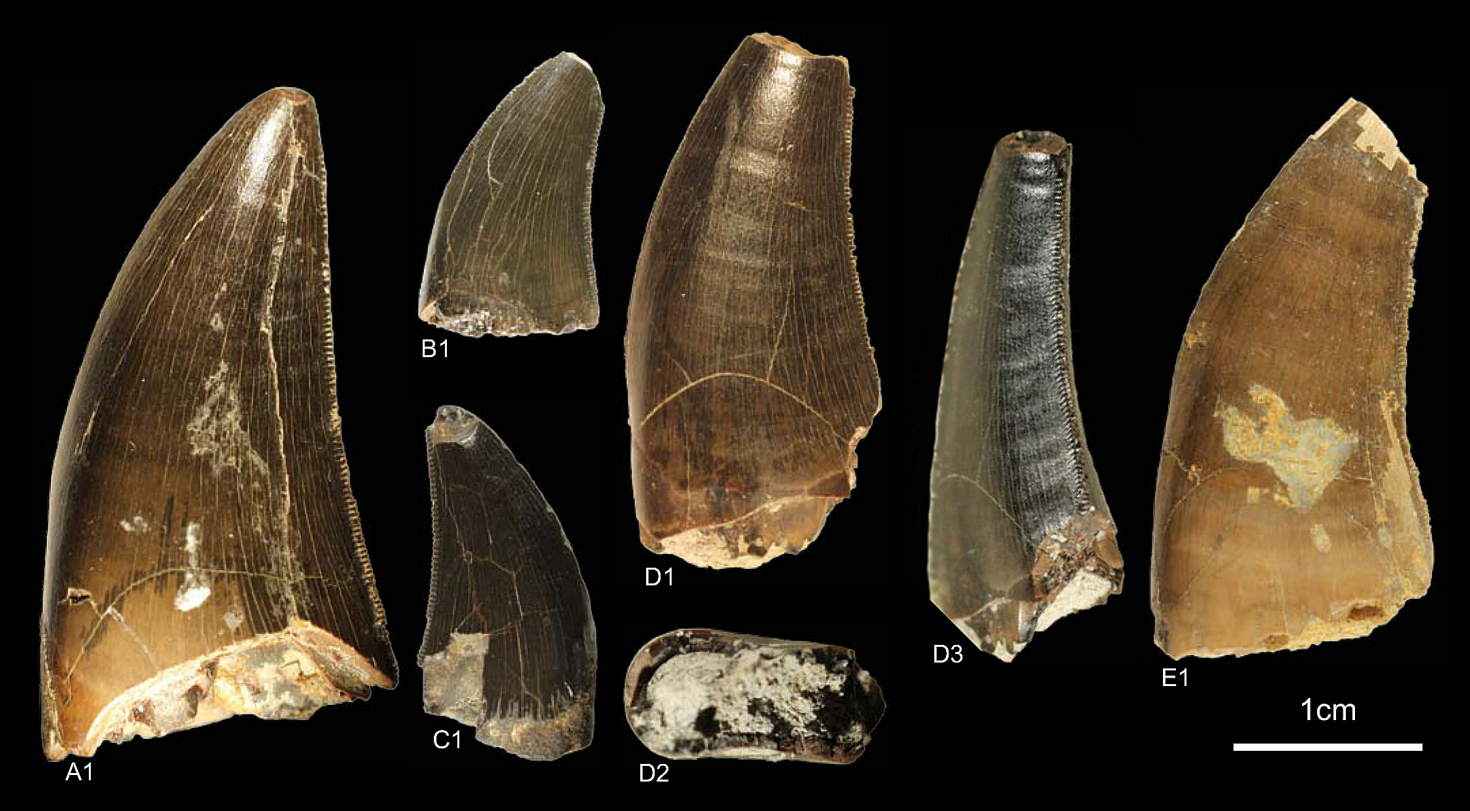
**Morphotype C:** A1, **GZG.V.010.329** in labial view; B1, **GZG.V.010.320** in labial view; C1, **GZG.V.010.344** in labial view; D, **NLMH101376a**, D1 in labial, D2 basal and D3 oblique distal view; E, **NLMH101377** in labial view.

GZG.V.010.320, GZG.V.010.329, GZG.V.010.344, NLMH101376a, NLMH101377 (Fig 7)

Morphotype C comprises four teeth from Ahlem, Lindener Berg and Tönniesberg in Hannover (Kimmeridgian). GZG.V.010.329 was collected in Holzen (Late Kimmeridgian).

#### Description

The crowns of morphotype C are almost complete, except for NLMH101376a, NLMH101377, and GZG.V.010.320 were the tips are missing. Mesial denticles are only preserved in GZG.V.010.320 and are quadrangular in lateral view. The distal denticles are horizontal rectangular; their orientation varies from perpendicular to slightly inclined apically. The denticulation is comparatively fine, e.g. in GZG.V.010.329 (CH 30) MC counts 16.25 and DC 16.5. The centered, non-twisted mesial carinae extend down to the cervix. The distal carinae are labially displaced, in GZG.V.010.320 also sigmoid-shaped in distal view and terminate at the cervix. With a CHR of 1.54–1.71 the crowns are rather stout. The basal cross-section is eight-shaped, in GZG.V.010.320 bean-shaped and there is always a shallow depression present on the labial side of the crown. The CBR of 0.41–0.47 denotes morphotype C as labio-lingually compressed. More or less well developed interdenticular sulci are present at the denticles of the distal carinae, as well as pronounced transversal undulations running mesio-distally across the crown.

#### Results and Discussion

Morphotype C is classified (S4 Appendix) with a strict consensus of two most parsimonious trees as close to Ceratosauria by the cladistic analysis. The results of the DFA (S1 Appendix) are ambiguous; the teeth were assigned to *Liliensternus*, *Berberosaurus*, *Raptorex* and *Neovenator*. This differs considerable from the results of the cladistic analysis and shows the size dependent classification of the DFA. Only NLMH101377 is classified (63.12%) as *Berberosaurus*. However, when the second rank is taken into account, GZG.V.010.329 (22.74%) and NLMH101376a (23.9%) are also regarded as *Berberosaurus*. Morphotype C shows also some similarities with tooth crowns described for the Chinese Late Jurassic theropod *Sinraptor*, e.g. the eight or bean-shaped basal cross-section, distal carinae that terminate at the cervix. *Sinraptor* belongs to the family Metriacanthosauridae [21], which is represented by *Metriacanthosaurus* from the Oxfordian of England, however, as no teeth were reported [95, 96] for this taxon, a comparison is not possible. Crowns of Ceratosauria possess a rather flat surface on the labial side of the teeth [1, 97] and concave surfaces along the mesial and/or distal carinae [1]. None of the previously mentioned features is present in morphotype C; only GZG.V.010.329 possesses a labial side where the mesio-distal curvature is not so pronounced and comparatively flat. It is the combination of other characters that groups morphotype C clearly close to Ceratosauria. The presence of Ceratosauria in Germany can be expected, as skeletal remains of *Ceratosaurus* are reported from the Late Jurassic of Europe [98]. We regard morphotype C as Ceratosauria *incerta sedis*.

### Morphotype D

#### Material

**Fig 8:**
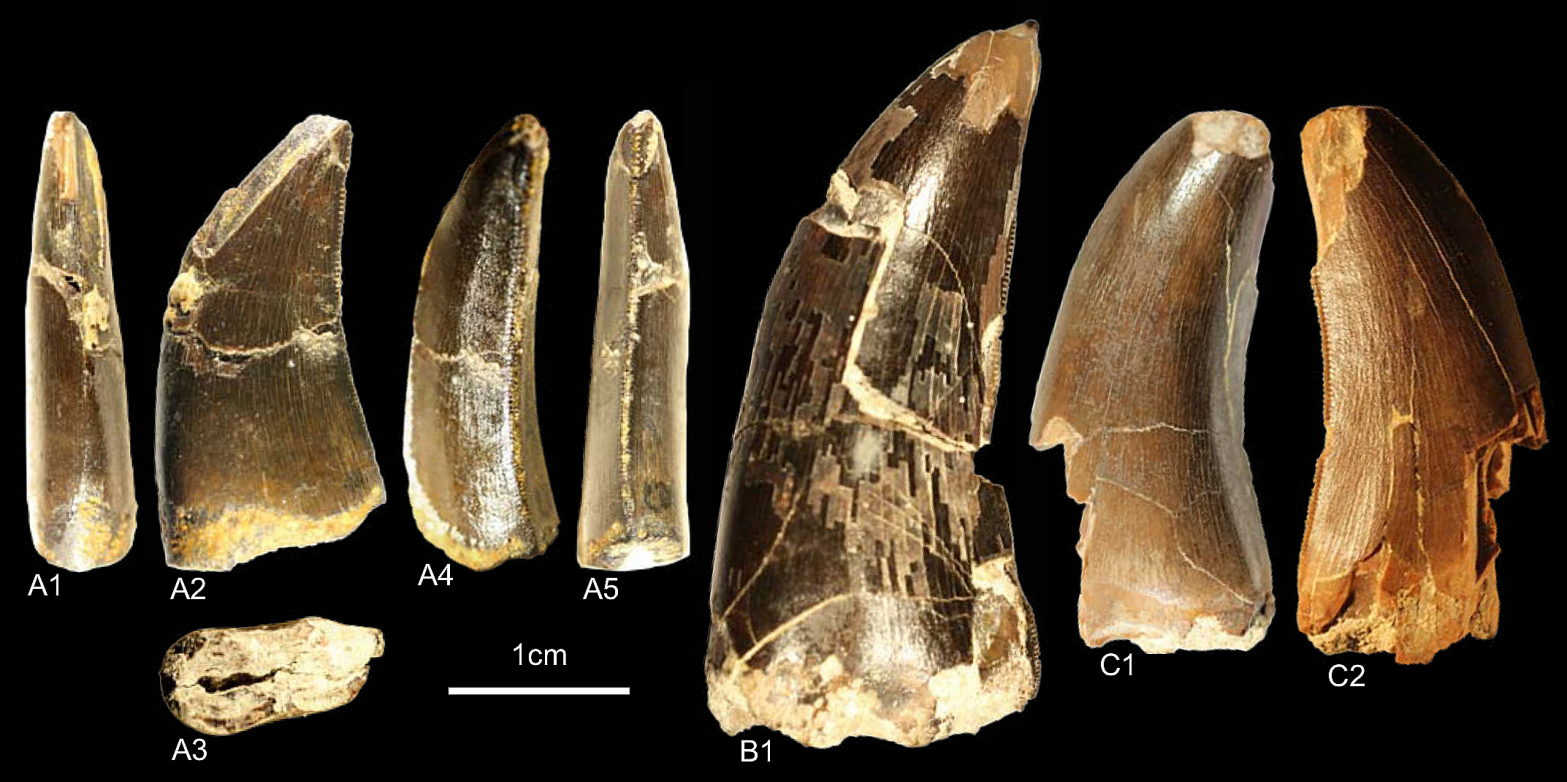
**Morphotype D:** A, **GZG.V.010.325**, A1 in mesial, A2 labial, A3 basal, A4 oblique distal and A5 distal view; B1, **GZG.V.010.332**, in lingual view; C, **NLMH16480**, C1 in lingual and C2 labial view.

GZG.V.010.325, GZG.V.010.332, NLMH16480 (Fig 8)

Morphotype D consists of three teeth; two from Ahlem and Lindener Berg in Hannover (Kimmeridgian) and one from the “?Oxfordian” (probably Kimmeridgian [23]) of Holzen.

#### Description

The teeth of morphotype D are similar to morphotype C (S4 Appendix, S10 Appendix). The CH ranges from 20.4–35.2 and CHR from 1.63–2.07. Morphotype D has less pronounced interdenticular sulci and transversal undulations than morphotype C. The lingual surfaces adjacent to the distal carinae are flat. In GZG.V.010.325 there is also a concave surface present at labial side of the distal carina. Contrary to morphotype C, morphotype D has centrally positioned mesial carinae terminating at midcrown. The mesial denticles (MC 16.25–20) of morphotype D are worn and the horizontal rectangular distals (DC 15–16.25) are slightly inclined apically. The surface texture is faint braided-oriented.

#### Results and Discussion

Given the similarities to morphotype C (S4 Appendix, S10 Appendix) it is not unexpected that the cladistic analysis recovers these teeth also close to Ceratosauria. Like in morphotype C, this placement is owed to the combination of characteristics. The only noticeable differences between morphotype D and the teeth of the contemporaneous *Ceratosaurus* are that in morphotype D the mesial carinae terminate well above the cervix [1] and the absence of a flat surface on the labial sides of the crowns. The DFA results (S1 Appendix) coincide partially: GZG.V.010.332 and NLMH16480 are classified as *Berberosaurus*, but with a poor probability (48.22% and 36.69%). The smallest crown is, however, assigned to Alioramus (42.81%). Similar to morphotype C, morphotype D is considered as Ceratosauria *incerta sedis*.

### Morphotype E

#### Material

**Fig 9:**
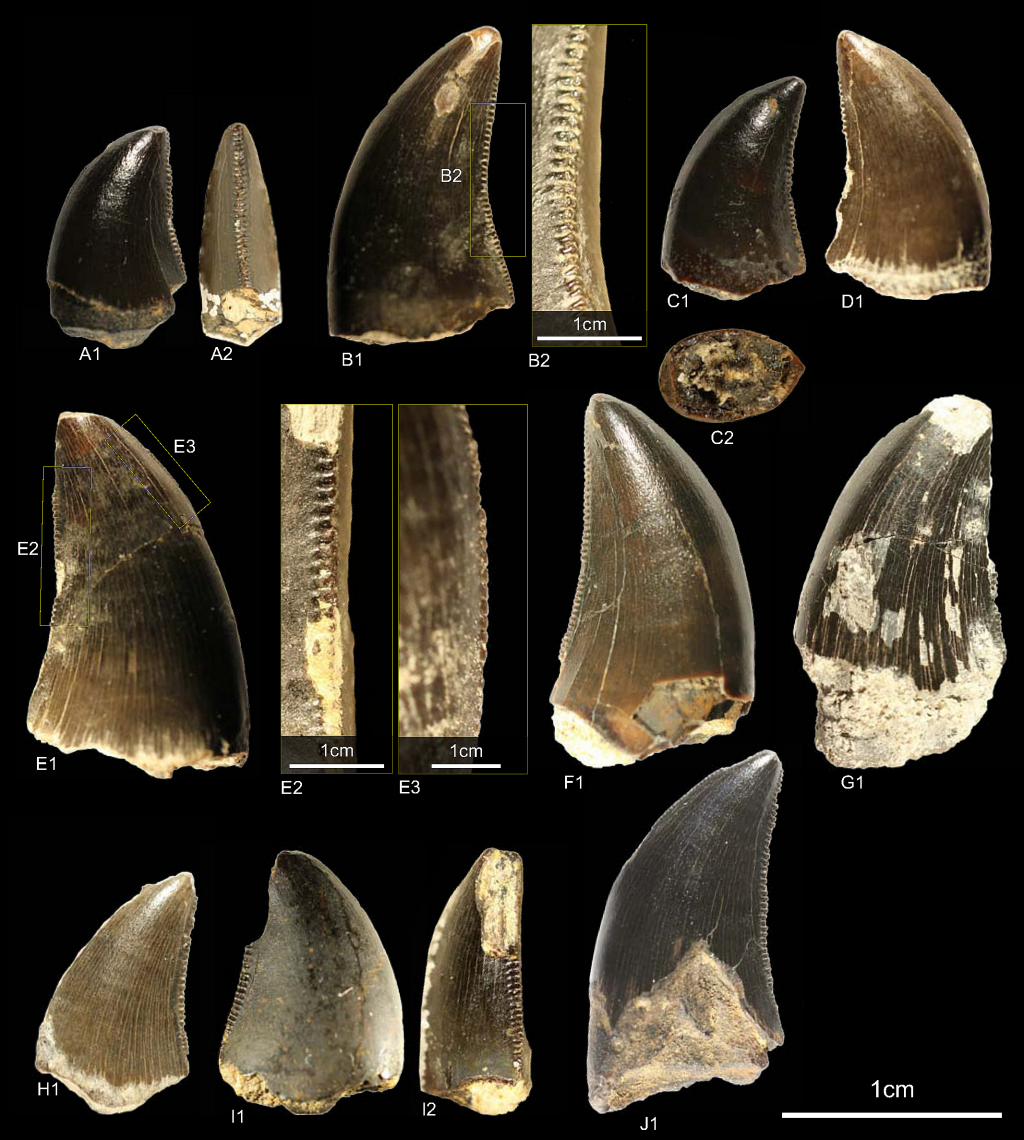
**Morphotype E:** A, **GZG.V.010.377**, A1 in lingual and A2 distal view; B, **GZG.V.010.379**, B1 in lingual and B2 details of the distal carina; C, **GZG.V.010.374**, C1 in lingual and C2 basal view; D1, **GZG.V.010.378** in lingual view; E, **GZG.V.010.380**, E1 in lingual view, E2 details of the distal and E3 mesial carina; F1, NLMH101378c in labial view; G, **NLMH101380e**, G1 in lingual view; H1, **NLMH106235c** in labial view; I, **RPM.NKP14359**, I1 in labial and I2 olique distal view; J1, **GSUBV4023** in labial view.

GSUBV4023, GZG.V.010.374, GZG.V.010.377, GZG.V.010.378, GZG.V.010.379, GZG.V.010.380, NLMH101378c, NLMH101380e, NLMH106235c, RPM.NKP.14359 (Fig 9)

All ten teeth of morphohotype E were collected at the Lindener Berg and Tönniesberg in Hannover (Kimmeridgian).

#### Description

The crowns of morphotype E are with a CH of 8.2–15 rather small and stout (CHR 1.26–1.50). Most obvious character is that the mesial denticles are smaller than the distals; the DSDI ranges from 1.25–2. The distal margin of the teeth varies from relatively straight, so that the apex does not extend beyond the crown base, to recurved (CA 49.36°–62°) in lateral view. The basal cross-section is lanceolate to slightly elliptical (CBR 0.53–0.7); only in GZG.V.010.379 there is lingually a shallow basal depression present. The mesial carinae terminate around midcrown and in NLMH101378c and GZG.V.010.380 they are twisted lingually. Profile and position of the distal carina varies; in some crowns it is offset from the midline and slightly sigmoid-shaped in distal view. Where preserved, the mesial denticles (22.5–35) are vertical rectangular and the apicals are perpendicular-oriented to the mesial margin. The distal denticles (DC 15–20) are horizontal rectangular, mainly perpendicular-oriented and possess an asymmetrically convex distal margin in lateral view. Few weak transversal undulations are present, as well as interdenticular sulci, but only at midcrown of the distal carinae. The enamel surface has a faint braided-oriented texture [1].

#### Results and Discussion

The cladistic analysis classifies (S4 Appendix) morphotype E close to *Piatnitzkysaurus* and Tyrannosauroidea. When the very similar morphotypes F—G are included, or *Piatnitzkysaurus* was excluded from the analysis, morphotype E is recovered as Tyrannosauroidea. The DFA assigns (S1 Appendix) most teeth of morphotype E to *Liliensternus* (48.83–94.68%); only GZG.V.010.378 and NLMH101380e are classified as *Deinonychus* (56.86–74.63%) and NLMH106235c as *Dubreuillosaurus* (32.12%). This is not surprising as *Liliensternus* and *Deinonychus* have comparable crown sizes and serration densities, whereas the teeth of basal Tyrannosauroidea (*Alioramus*, *Raptorex*, and *Proceratosaurus)* have, by similar crown sizes, either finer or coarser serrations. The results of the cladistic analysis coincide with rare skeletal material described in the fossil record of Europe. This material comprises the basal Tyrannosauroidea *Avityrannis* [99] from the Late Jurassic (Kimmeridgian) of Portugal and *Juratyrant* [100, 101] from the Late Jurassic (lower Tithonian) of England. However, as no teeth are reported for these taxa, this assignment could not be verified. Morphotype E is regarded as cf. Tyrannosauroidea.

### Morphotype F

#### Material

**Fig 10:**
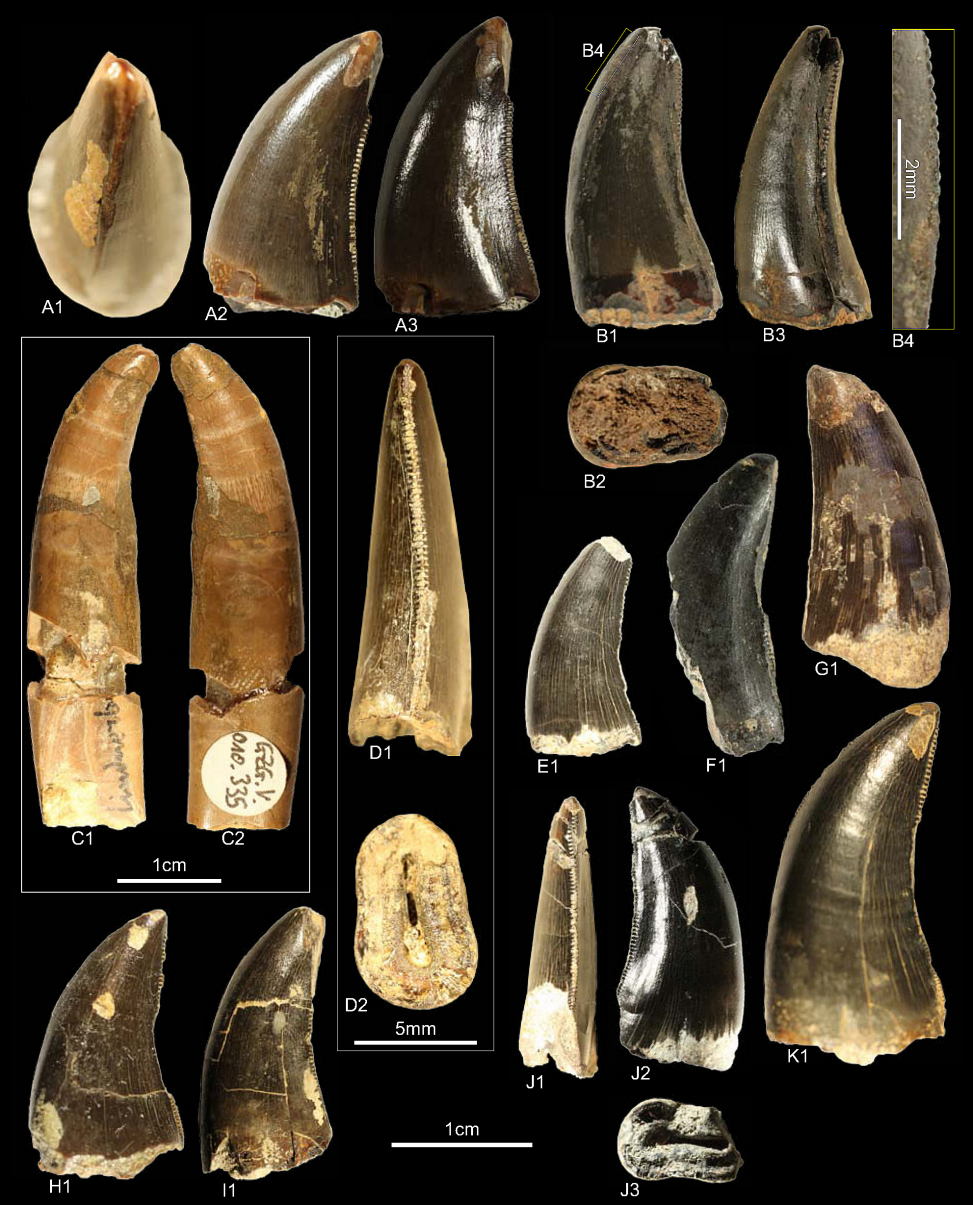
**Morphotype F:** A, **NLMH101378b**, A1 in apical and A2, A3 labial view; B, **GZG.V.010.345**, B1 in labial, B2 basal, B3 oblique distal view and B4 details of the mesial carina; C, **GZG.V.010.335**, C1 in lingual and C2 labial view; D, **GZG.V.010.370**, D1 in distal and D2 basal view; E1, **NLMH101380b** in labial view; F1, **NLMH106235d** in labial view; G, **NLMH16416a** in lingual view; H1, **GZG.V.010.326** in labial view; I1, **GZG.V.010.328** in labial view; J, **NLMH101380a**, J1 in distal, J2 labial and J3 basal view; K1, **GZG.V.010.371** in labial view.

GZG.V.010.326, GZG.V.010.328, GZG.V.010.335, GZG.V.010.345, GZG.V.010.370, GZG.V.010.371, NLMH101378b, NLMH101380a, NLMH101380b, NLMH106235d, NLMH16416a (Fig 10)

All eleven teeth of morphotype F were collected at the Lindener Berg and Tönniesberg in Hannover (Kimmeridgian).

#### Description

Similar to morphotype E, morphotype F has mesial denticles larger than the distals (DSDI 1.21–2) and show comparable CBR (0.52–0.70). Where preserved, both morphotypes have similar denticle morphology as well as MC and DC (Morphotype F, MC 18.75–35, DC 15–17.5) range. The surface structure is also braided-oriented, with interdenticular sulci present only at midcrown of the distal carinae. The transversal undulations are sparsely distributed. Most obvious differences are that morphotype F is larger (CH 14.7–21.8) and more elongate (CHR 1.54–2.04). There is always a shallow depression present on the labial side and in some crowns also lingually, giving the basal cross-section a bean or eight shape. Some crowns possess a shallow depression that runs along the labial side of the distal carinae. GZG.V.010.335 is the only one tooth of this study where the root is preserved. In lateral view, the root-margins are sub-parallel and not thicker than the base of the crown. There are numerous transversal undulations visible whereas in the tooth crown only few are present. The cross-section at midroot is eight-shaped and the lingual fossa for the erupting tooth is deeper than the labial depression.

#### Results and Discussion

The cladistic analysis places (S4 Appendix) morphotype F in 22 of 30 cases within Tyrannosauroidea, and the remaining eight within Dromaeosauridae. This results in a strict consensus tree that classifies morphotype F as Coelurosauria. The outcome presented by the DFA (S1 Appendix) is also indecisively. The teeth were assigned to *Deinonychus*, *Piatnitzkysaurus*, *Raptorex* and *Proceratosaurus* with an as well variable classification probability (40.99–75.76%). As these results are rather indecisively, a separate DFA and principal component analysis (PCA) for morphotypes E—G (S8 Appendix) was run and they were tested against taxa with a DSDI of >1.2 [1, 61]: *Piatnitzkysaurus*, *Masiakasaurus*, *Liliensternus*; the higher level clades basal Tyrannosauroidea (*Proceratosaurus*, *Alioramus*, *Raptorex*) and Dromaeosauridae (*Deinonychus*, *Dromaeosaurus*, *Velociraptor*). The variables used comprise the same as for the main analysis and the DFA was executed with default options (priors =“observed”). This analysis classifies morphotype F as “Tyrannosauroidea” and the classification probabilities are relatively robust (71.88–99.01%). Dromaeosaurid teeth are very similar to crowns of basal Tyrannosauroidea [1, 75]. However, the oldest unquestionable dromaeosaurids are reported from the Early Cretaceous [102, 103], whereas the lineage of Tyrannosauroidea reaches back to the Middle Jurassic [75, 104]. Older records of Dromaeosauridae are only represented by isolated teeth [75, 103]. The main argument for this assignment is that the distal denticles are larger than the mesial ones [75]. However, this is not a unique feature of the Dromaeosauridae; it is also reported for Tyrannosauroidea and several other taxa [1]. Like in morphotype E, the assignment of morphotype F to basal Tyrannosauroidea becomes clearer when the similar morphotypes E and G were integrated in the cladistic analysis. Putative tyrannosauroid teeth from the Kimmeridgian of Portugal [105] show similarities with teeth of morphotypes F and G e.g., the rather rounded cross-section in some crowns, the morphology of the mesial denticles, and a concave surface adjacent to the distal carinae. Based on these comparisons and the results of the statistical analysis, morphotype F is regarded as basal Tyrannosauroidea.

### Morphotype G

#### Material

**Fig 11:**
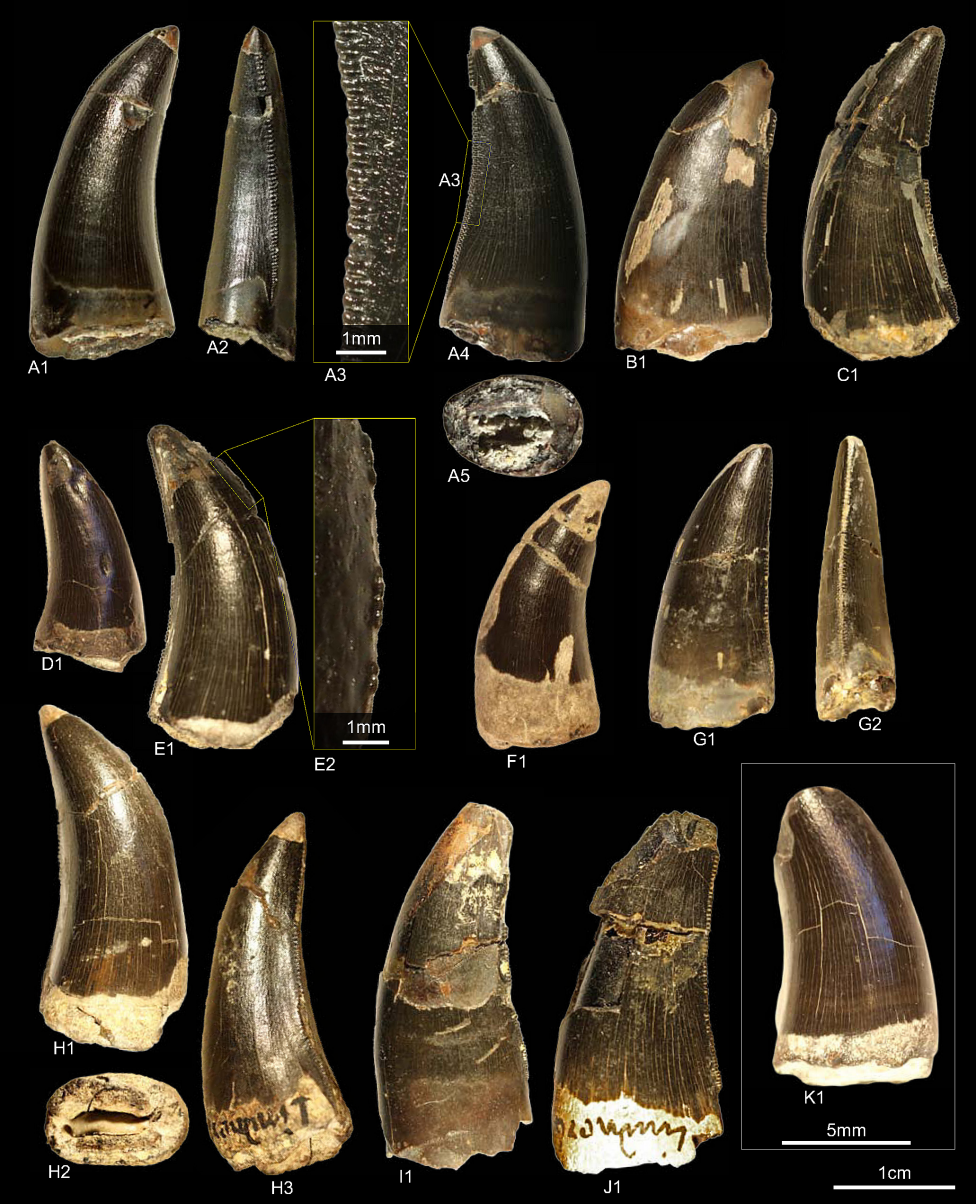
**Mophotype G:** A, **NLMH101378a**, A1 in lingual and A2 distal view, A3 details of the distal carniae, A4 in labial and A5 basal view; B1, **RPM.NKP14358** in labial view; C1, **GZG.V.010.331** in labial view; D1, **NLMH101376b** in labial view; E, **GZG.V.010.331**, E1 lingual view, E2 details of the mesial carina; F1, **NLMH101376d** in labial view; G1, **GZG.V.010.372**, G1 in labial and G2 distal view; H, **GZG.V.010.318**, H1 in lingual, H2 basal and H3 labial view; I1, **GZG.V.010.322** in labial view; J1, **GZG.V.010.323** in labial view; K1, **NLMH101376e** in lingual view.

GZG.V.010.318, GZG.V.010.321, GZG.V.010.322, GZG.V.010.323 GZG.V.010.331, GZG.V.010.372, NLMH101376, NLMH101376d, NLMH101376e, NLMH101378a, RPM.NKP.14358 (Fig 11)

Ten teeth were collected at the Lindener Berg and Tönniesberg in Hannover (Kimmeridgian); GZG.V.010.331 was found in Holzen (Late Kimmeridgian).

#### Description

The teeth of morphotype G are very similar to morphotype F so that the transition is sometimes indistinct. They are of similar size (CH 10.5–26) and possess a comparable elongation of the crowns (CHR 1.62–1.96), as well as distal denticles that are larger than the mesials (DSDI 1.33–1.57). They share a similar CBR (0.54–0.75) but differ from morphotype F in the absence of a labial depression resulting in a lanceolate to ovoid basal cross-section. In some crowns, however, there is also a very shallow lingual depression present so that the basal cross-section is rather bean-shaped. Contrary to morphotype F, the distal carinae are always sigmoid-shaped in distal view and in some crowns they bear interdenticular sulci not only at midcrown but also near the cervix. The distal denticles are quadrangular to slightly horizontal rectangular-shaped and the external margin is asymmetrically convex in lateral view.

#### Results and Discussion

Like morphotype E, the cladistic analysis classifies (S4 Appendix) these teeth also close to *Piatnitzkysaurus* and Tyrannosauroidea. When *Piatnitzkysaurus* is removed or morphotypes E and F are included then morphotype G is classified as Tyrannosauroidea. The DFA assigns (S1 Appendix) morphotype G to *Piatnitzkysaurus* and *Deinonychus* and show that there is a size component in this classification, however with a variable (24.92–90.22%) classification probability. Given the similarities of morphotype G to F it is possible that these teeth belong to one taxon. As in morphotype F, some crowns of morphotype G resemble teeth described from the Kimmeridgian of Portugal [105]. For details see discussion on morphotype F. Morphotype G is considered in this study as a basal Tyrannosauroidea.

### Morphotype H

#### Material

**Fig 12:**
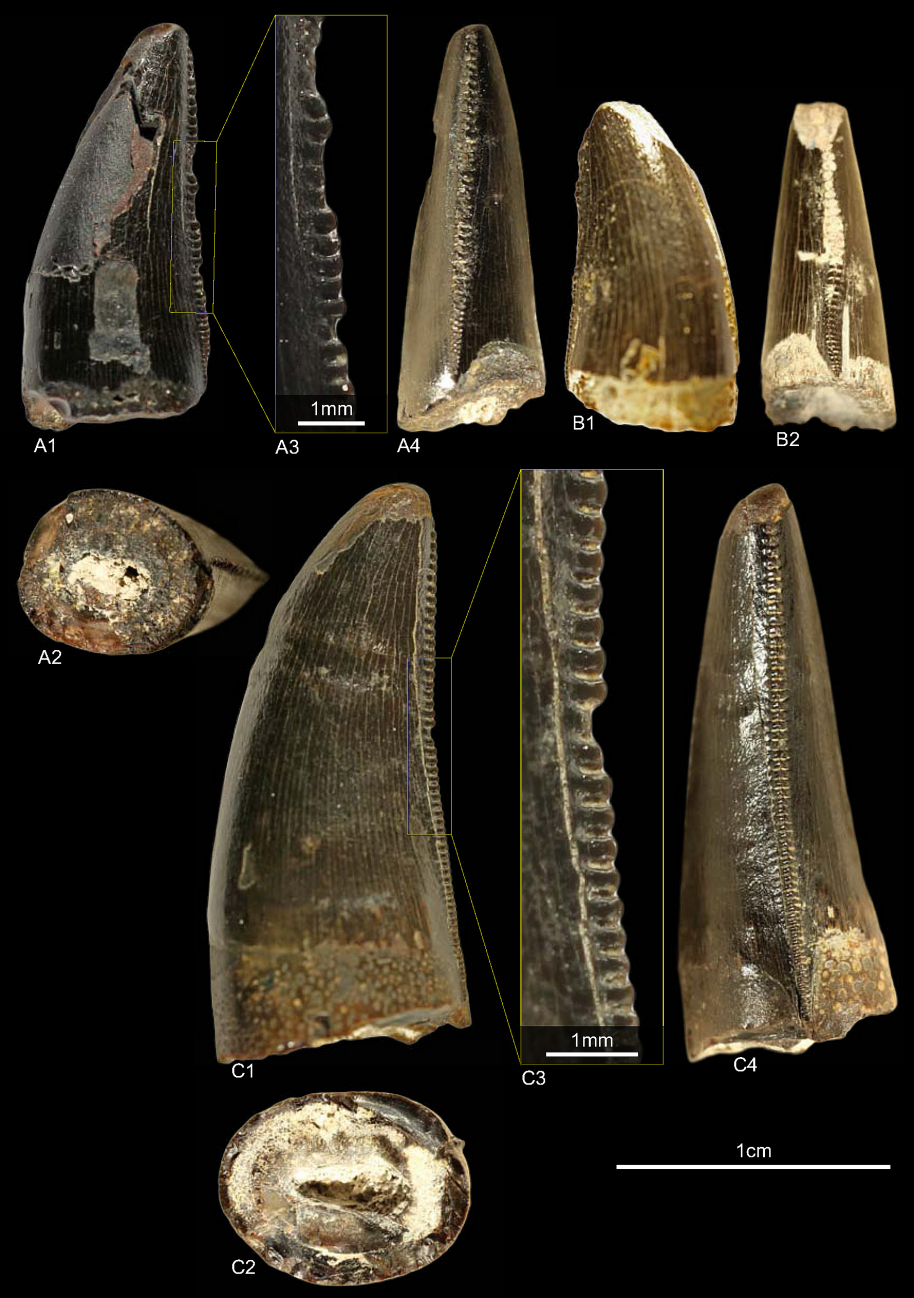
**Morphotype H:** A, **GSUBV4022**, A1 in lateral, A2 basal and A4 distal view; A3 details of the distal carina, B, **GZG.V.010.369**, B1 in labial and B2 in distal view; C, **GZG.V.010.373**, C1 in labial and C2 basal view, C3 details of the distal carina, C4 distal view.

GSUBV4022, GZG.V.010.369, GZG.V.010.373 (Fig 12)

All three teeth were collected at the Lindener Berg and Tönniesberg in Hannover (Late Kimmeridgian).

#### Description

The most obvious character of morphotype H is a near circular basal cross-section (CBR 0.79–0.82). In lateral view the distal margin is rather straight so that the apex does not extend beyond the base. Morphotype H comprises rather small teeth (CH 10–17.2) and the CHR ranges from 1.59–2.06. Mesial denticles are not preserved but apparently present as in GZG.V.010.373 faint imprints could be seen where the enamel is broken off. If this interpretation is correct, GZG.V.010.373 has a high DSDI of 1.66. The denticulated distal carinae terminate at the cervix, except for GZG.V.010.373 where it terminates slightly below. The distal denticles resemble those of morphotype E—G and have also a similar serration density (DC 15).

#### Results and Discussion

Morphotype H was initially coded as lateral teeth, as it shares many similarities with morphotype G (especially GZG.V.010.372). The only differences are that GZG.V.010.373 has a more circular cross-section (CBR 0.81); GZG.V.010.372 is more elliptical to lanceolate (CBR 0.7) and the distal carina terminates slightly below the cervix. Both crowns are not well-preserved, with, e.g. the mesial denticles missing. The results (S4 Appendix) of the cladistic analysis are equivocal and treat morphotype H as Theropoda. Only when analyzed together with the previous morphotype, they were also classified as Tyrannosauroidea. With the subcircular to elliptical basal cross-section it could be possible that morphotype H represents mesialmost teeth [1]. Rerunning the cladistic analysis with morphotype H coded as mesialmost it is also classified as Theropoda. Two DFA were conducted because there are no mesial denticles preserved and the denticle count for GZG.V.010.373 is rather uncertain. In the first, the uncertain MC of GZG.V.010.373 was included and in the second variable MC was replaced with CBR. In the first DFA, with included MC, GZG.V.010.373 is assigned (S1 Appendix) to *Deinonychus* (53.73%) and on the second rank to *Proceratosaurus* (28.71%). The second DFA, with CBR instead of MC, recovers (S1 Appendix) all teeth of morphotype H as *Dubreuillosaurus* mesialmost (48–91.08%). Morphotype H resembles the premaxillary teeth of *Dubreillosaurus* in some details, however, differences exists as well, e.g. the presence of interdenticular sulci and asymmetrically convex distal margin of the distal denticles in lateral view. Morphotype H is regarded here as probable mesialmost tooth and could not be classified beyond Theropoda indet.

### Morphotype I

#### Material

**Fig 13:**
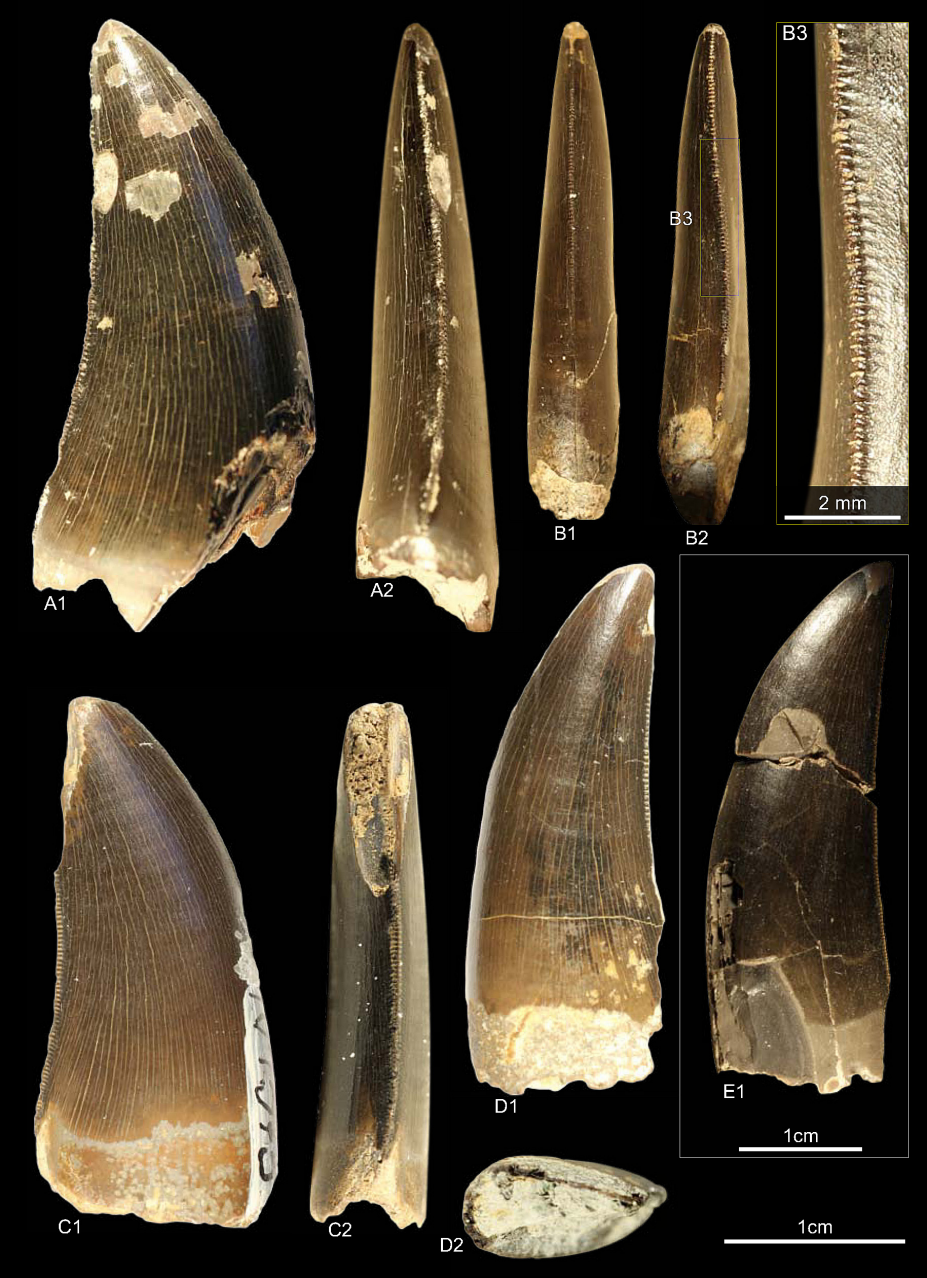
**Morphotype I:** A, **GZG.V.010.319**, A1 in labial and A2 distal view; B, **GZG.V.010.327**, B1 in distal and B2 mesial view, B3 details of the distal carina; C, **NLMH101375a**, C1 in labial and C2 distal view; D, **NLMH101376c**, D1 in labial and D2 basal view; E **RPM.NKP14357**, E1 in labial view.

GZG.V.010.319, GZG.V.010.327, NLMH101375a, NLMH101376c, NLMH16416c, cf. RPM.NKP.14357 (Fig 13)

All six teeth were found in Ahlem, Lindener Berg and Tönniesberg (Hannover, Kimmeridgian).

#### Description

The tooth crowns (CH 25.5–39) of morphotype I are of a moderate to elongated appearance (CHR 1.97–2.47). They are slightly recurved (CA 55.04°–65.7°) and moderate labio-lingually compressed (CBR of 0.49–0.55) with a lanceolate basal cross-section. The lingual surface is relatively flat, the enamel texture finely braided and well-developed interdenticular sulci are present even at the mesial carinae. The distal carinae extend to the cervix. The profiles of the distal carinae are sigmoid-shaped, in NLMH101376c more straight, and labially displaced in distal view. The non-twisted mesial carinae terminate at midcrown. The midcrown distal denticles are horizontal rectangular-shaped and perpendicular oriented to the distal margin in lateral view, whereas the mesials are rather quadrangular. The interdenticular space of the midcrown distal denticles is broad. With a DSDI of 1.07–1.15 the distals are slightly larger than the mesial denticles. GZG.V.010.327 represents the most complete crown of morphotype I, whereas in RPM.NKP.14357 the lingual side is still enclosed in matrix. In NLMH101376c there is a part of the lingual base of the crown and the mesial carina missing.

#### Results and Discussion

The cladistic analysis places (S4 Appendix) morphotype I within Allosauroidea close to teeth of *Sinraptor*, a theropod of the family Metriacanthosauridae [19] from the Late Jurassic of China. This family is represented in Europe with *Metriacanthosaurus* [95, 96] from the Oxford Clay of England. As there is no tooth material known from this taxon, a comparison is not possible. Despite their placement close to *Sinraptor*, several differences exist, such as the outline of the basal cross-section and the rather straight and non-twisted distal carinae in distal view. The Late Jurassic taxon *Ceratosaurus* from North America, also reported from Europe [98], possesses teeth that also have a rather elongated appearance and similar shape of the denticles [1, 105], although elongate crowns in some teeth are a common feature in theropods [1, 2]. However, there are also some differences like, e.g. mesial carinae that terminate at the cervix and the presence of concave surfaces along the carinae in *Ceratosaurus* crowns [1]. The DFA results classify (S1 Appendix) GZG.V.010.319 (41.08%) and NLMH101375a (45.34%) as *Neovenator* and GZG.V.010.327 (49.49%), NLMH101376c (with CBR instead of MC 17.12%) and RPM.NKP.14357 (39.77%) as *Erectopus*. The DFA results support therefore the assignment of the cladistic analysis of morphotype I to Allosauroidea, if *Erectopus* remains within the Allosauroidea [106]. Following the collection label and figure presented by Graf Georg zu Münster [18], one tooth of morphotype I probably represents the specimen, possibly NLMH101375a, originally described as the fossil fish “*Saurocephalus Monasterii*” [18], which was later transferred to *Megalosaurus* [19].

### Morphotype J

#### Material

**Fig 14:**
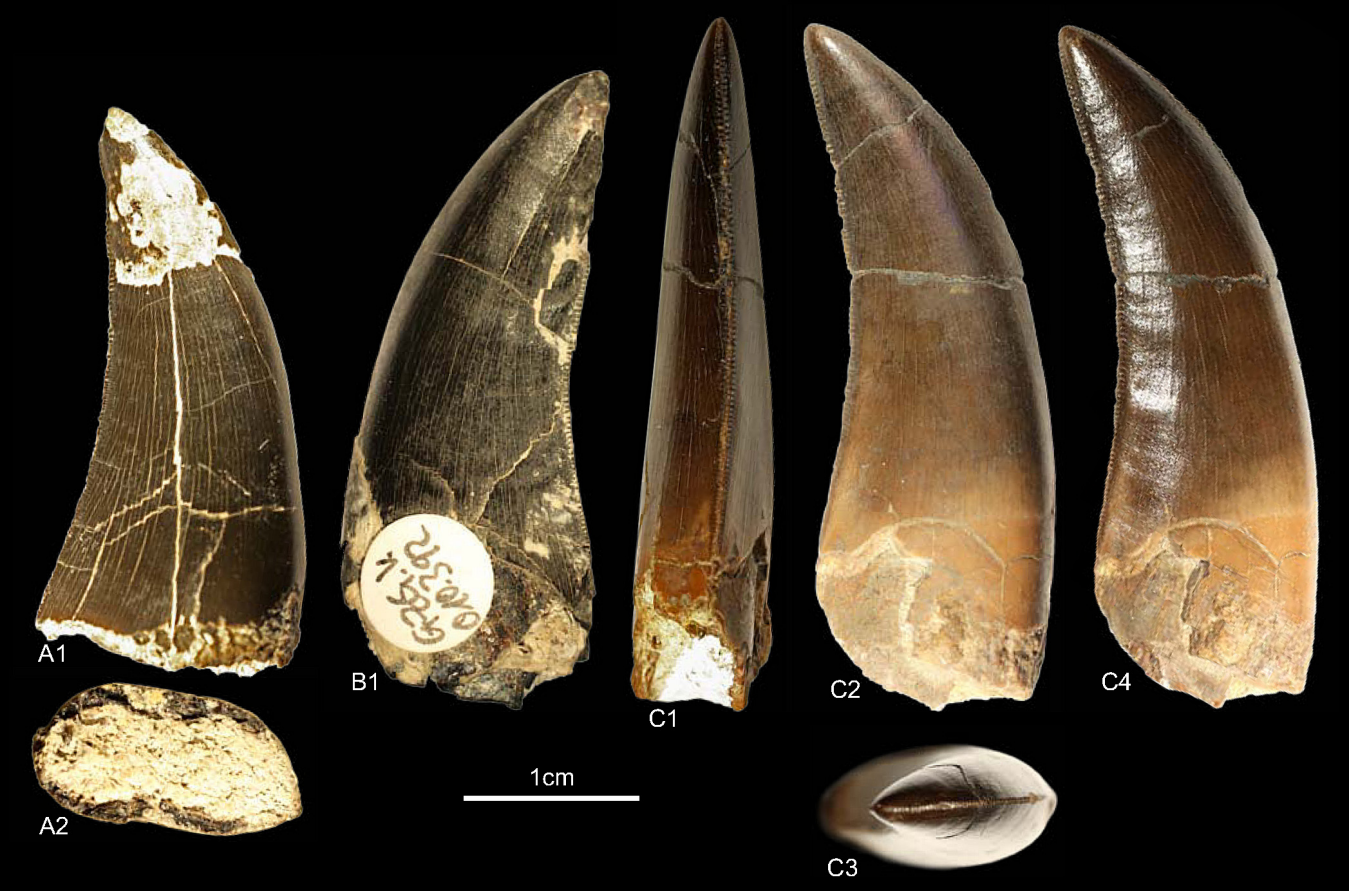
**Morphotype J:** A, **GZG.V.010.330**, A1 in lingual and A2 basal view; B1, **GZG.V.010.392** in labial view; C, **NLMH106235b**, C1 in distal, C2 labial, C3 apical and C4 oblique labial view.

GZG.V.010.330, GZG.V.010.392, NLMH106235b (Fig 14)

Morphotype J comprises two teeth from the Lindener Berg and Tönniesberg of Hannover (Kimmeridgian) and one tooth from Holzen, labeled as “?Oxfordian” (Late Kimmeridgian, [23]).

#### Description

The specimens representing morphotype J are similar to the smaller teeth of morphotype I regarding the morphometric measurements CH (29.8–30.5), CBR (0.54–0.58), CHR (1.85–2.14) and DSDI (1–1.2). They differ from morphotype I in possessing a slightly narrower interdenticular space, absence of interdenticular sulci at the lower part of the distal carinae which terminate well below the cervix, and slightly larger mesial denticles at midcrown that become coarser towards the apex.

#### Results and Discussion

The DFA classifies (S1 Appendix) two teeth of morphotype J as *Neovenator* (41.27 and 46.97%) and NLMH106235b to *Piatnitzkysaurus* (50%), however, with a rather poor posterior probability. The cladistic analysis places (S4 Appendix) morphotype J with a strict consensus of eight most parsimonious trees within Megalosauridae. NLMH106235b, the most complete crown of morphotype J, shares several similarities with teeth described for *Marshosaurus* [2, 107], a member of the clade Piatnitzkysauridae [108]: A slender, elongated crown in lateral view; mesial denticles that are smaller than the distals; mesial carinae terminating at midcrown; the extent of the distal carinae and a comparable serration density at the distal carinae. However, the lateral teeth of *Marshosaurus* are smaller and seem to be more laterally compressed, except for RD4 of UMNH VP6368 [2]. When the second rank of the DFA with CBR instead of MC is taken into account, NLMH106235b is classified as *Marshosaurus* (22.72%). Madsen [107] published no measurements and the teeth of *Marshosaurus* are rather incomplete, hampering further comparisons. Morphotype J is treated here as pertaining to a member of the clade Megalosauroidea. The referral to *Marshosaurus* is only tentatively and requires further description of its dentition.

### Morphotype K

#### Material

**Fig 15:**
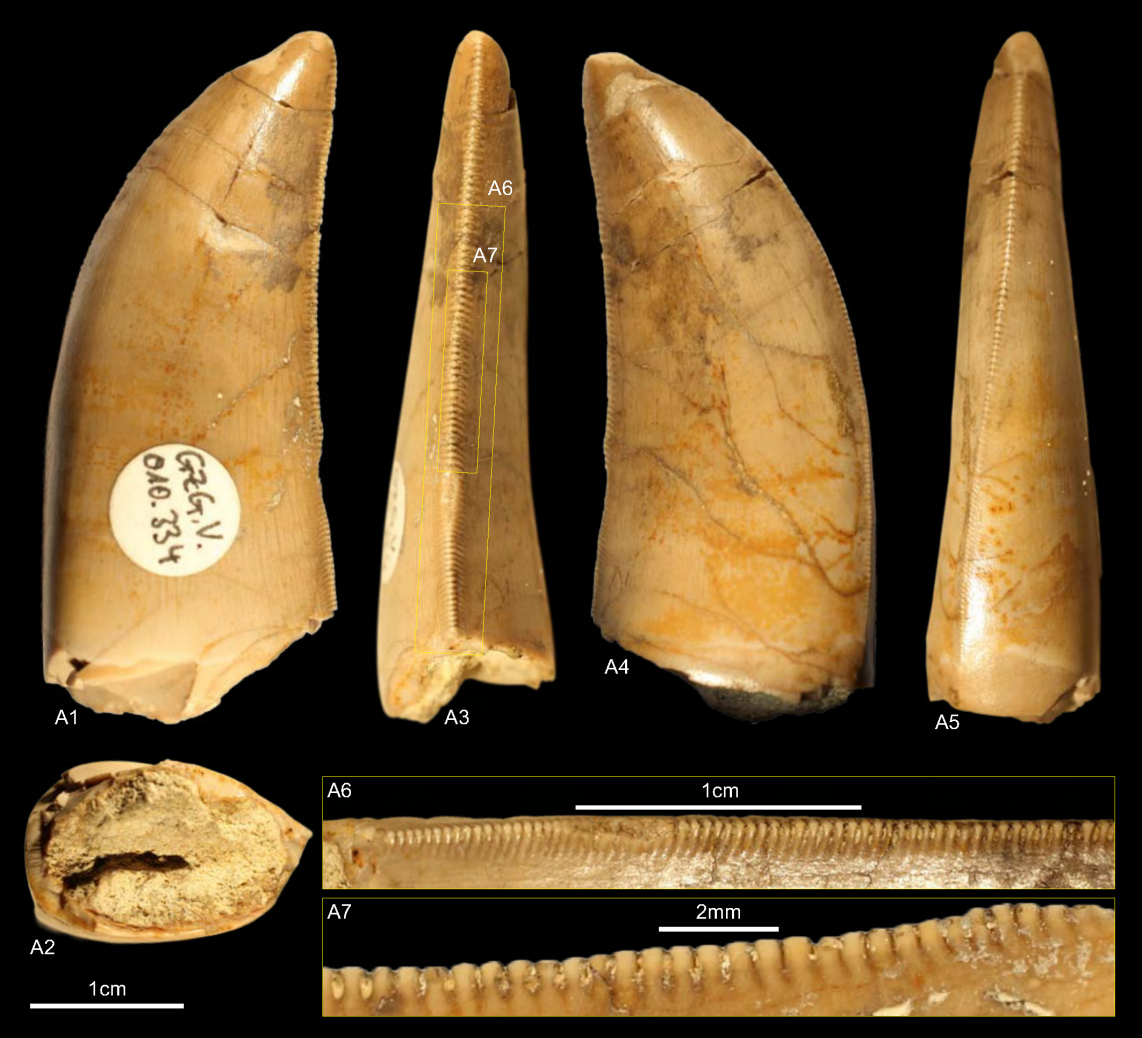
**Morphotype K:** A, **GZG.V.010.334**, A1 in labial, A2 basal, A3 distal, A4 lingual and A5 mesial view, A6 and A7 details of the distal carina.

GZG.V.010.334 (Fig 15)

One complete tooth collected at the Kahlberg near Echte (Late Kimmeridgian)

#### Description

The crown high measures 37.3. With a CHR of 1.96 and a CBR of 0.6 it is of a stout appearance. The denticle counts are 11 for DC and 12 for MC (DSDI 1.09). The basal cross-section is lanceolate and the distal margin only weakly recurved in lateral view. The mesial denticles are worn. The denticulated mesial carina runs at the first third of the apex straight and twists then strongly lingually near the base in mesial view. The distal carina is labially displaced and slightly sigmoid-shaped in distal view. The somewhat worn distal denticles are inclined apically and horizontal rectangular in lateral view and become progressively larger from the base to the apex. The interdenticular space of the distal denticles is rather wide and bears well-developed, basally curving interdenticular sulci at the entire carina. Contrary to most other morphotypes, the enamel surface is smooth to fine irregularly textured.

#### Results and Discussion

The cladistic analysis classifies (S4 Appendix) morphotype K as Allosauroidea. The outcome of the DFA is very indistinct (S1 Appendix). It assigns morphotype K to *Megalosaurus* (26.98%) and on the second rank to *Genyodectes* (21.36%). GZG.V.010.334 shows many similarities with teeth (ML327 and ML966) described by Hendrickx and Mateus [1] from the Late Jurassic (Late Kimmeridgian—Early Tithonian) of Portugal as pertaining to the Abelisauridae. With the lack of a longitudinal ridge, comparable recurvature and not apically hooked denticles, morphotype K resembles especially ML966. When including the Portuguese teeth in the cladistic analysis, GZG.V.010.334 is recovered in the strict consensus as Neotheropoda and TNT classifies it as either Abelisauridae or Allosauridae. When the analysis was run with morphotype K and ML966 only, then TNT places both within Allosauroidea. To further test this issue a second DFA (see S9 Appendix) including ML327 and ML966 was executed, with the result that morphotype K is now assigned to ML327, however with 33.89% the posterior probability is not very strong. When the prior probability of the DFA is set under the options menu to “observed” then GZG.V.010.334 is classified as *Allosaurus* (43.43%). Hendrickx and Mateus [1] supported the referral of ML327 and ML966 to Abelisauridae in the Late Jurassic of Portugal with bone material, among others, from the Middle Jurassic of Europe [109] and Late Jurassic of North America [110]. However, the Laurasian material has probably no abelisaurid affinities [111]. Morphotype K differs only in one character (98) from the teeth that Hendrickx and Mateus described as *Allosaurus* [1, 61], namely that in GZG.V.010.334 the mesial denticles become progressively coarser towards the apex, whereas in *Allosaurus* they are smaller apically than at midcrown. Despite of the noticeable similarity of morphotype K with abelisaurid teeth, we consider it plausible to regard GZG.V.010.334 as *Allosaurus* sp. until more complete material is found.

### Morphotype L

#### Material

**Fig 16:**
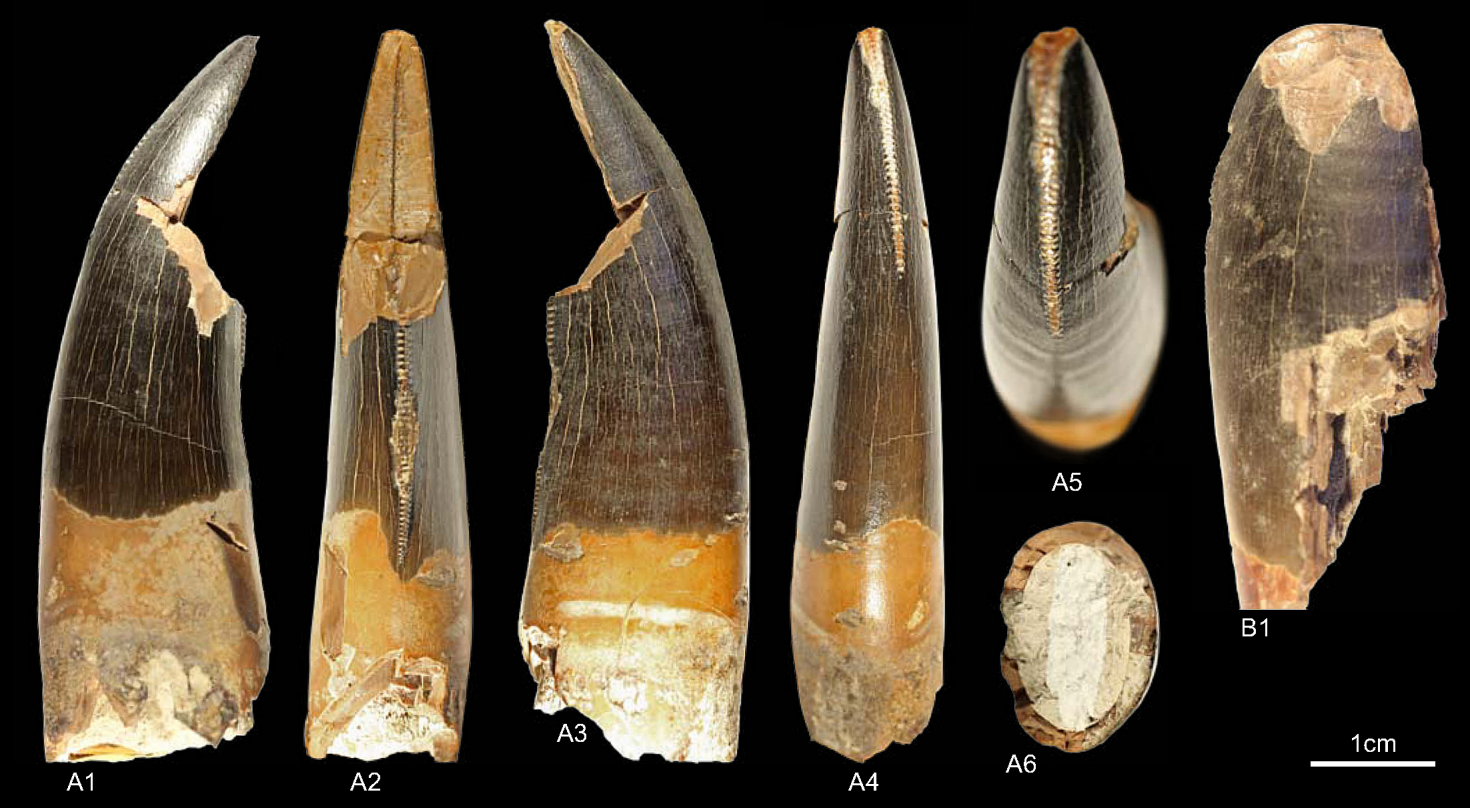
**Morphotype L:** A, **NLMH106235a**, A1 in lingual, A2 distal, A3 labial, A4 mesial, A5 in apical and A6 basal view; B, **NLMH16416b** in lateral view.

NLMH16416b, NLMH106235a (Fig 16)

Both teeth were found at the Tönniesberg (Late Kimmeridgian) in Hannover.

#### Description

Morphotype L comprises a tooth fragment (NLMH16416b) and a tooth crown (NLMH106235a) with the apical half of its distal part missing. Because NLMH16416b is very fragmentary, only NLMH106235a is described. With a CH of 40.7 it is one of the larger tooth of this study. NLMH106235a is elongated (CHR 2.39) with a subcircular to elliptical basal cross-section (CBR 0.72). The non-twisted mesial carina terminates around midcrown. The relatively coarse mesial denticles (MC 8) are worn. The preserved part of the distal carina is centrally positioned in distal view and terminates at the cervix. With a DC of 9 the distal denticles are smaller (DSDI 0.88) than the mesials and of quadrangular to somewhat subrectangular shape in lateral view. Interdenticular sulci are present and the interdenticular space is rather wide.

#### Results and Discussion

With a subcircular to elliptical basal cross-section NLMH106235a resembles in most details crowns described as megalosaurid mesialmost teeth [1, 59]. The cladistic analysis places (S4 Appendix) morphotype L (coded as mesialmost) with a strict consensus of 20 most parsimonious trees well within the Megalosauridae. The DFA classifies (S1 Appendix) NLMH106235a as *Allosaurus*. This is not a surprising result as *Allosaurus* teeth are comparable regarding morphometric measurements, but they differ from morphotype L in possessing often twisted and displaced mesial and distal carinae, distal carinae terminating well below the cervix and a more irregular surface texture [1]. The result of the DFA shows again that there is a size component in the classification. The mesialmost teeth of the megalosaurids *Torvosaurus* and *Dubreillosaurus* are either larger or smaller than NLMH106235a. Confirmed by the results of the cladistic analysis morphotype L is treated as mesialmost tooth of a megalosaurid theropod that shows great overlap in characteristics with a tooth (ML962) described from Late Jurassic of Portugal [1] and classified as mesialmost of *Torvosaurus*. It is possible that the teeth of morphotype L were the mesialmost of morphotype B and therefore pertain to one taxon.

### Morphotype M

#### Material

**Fig 17:**
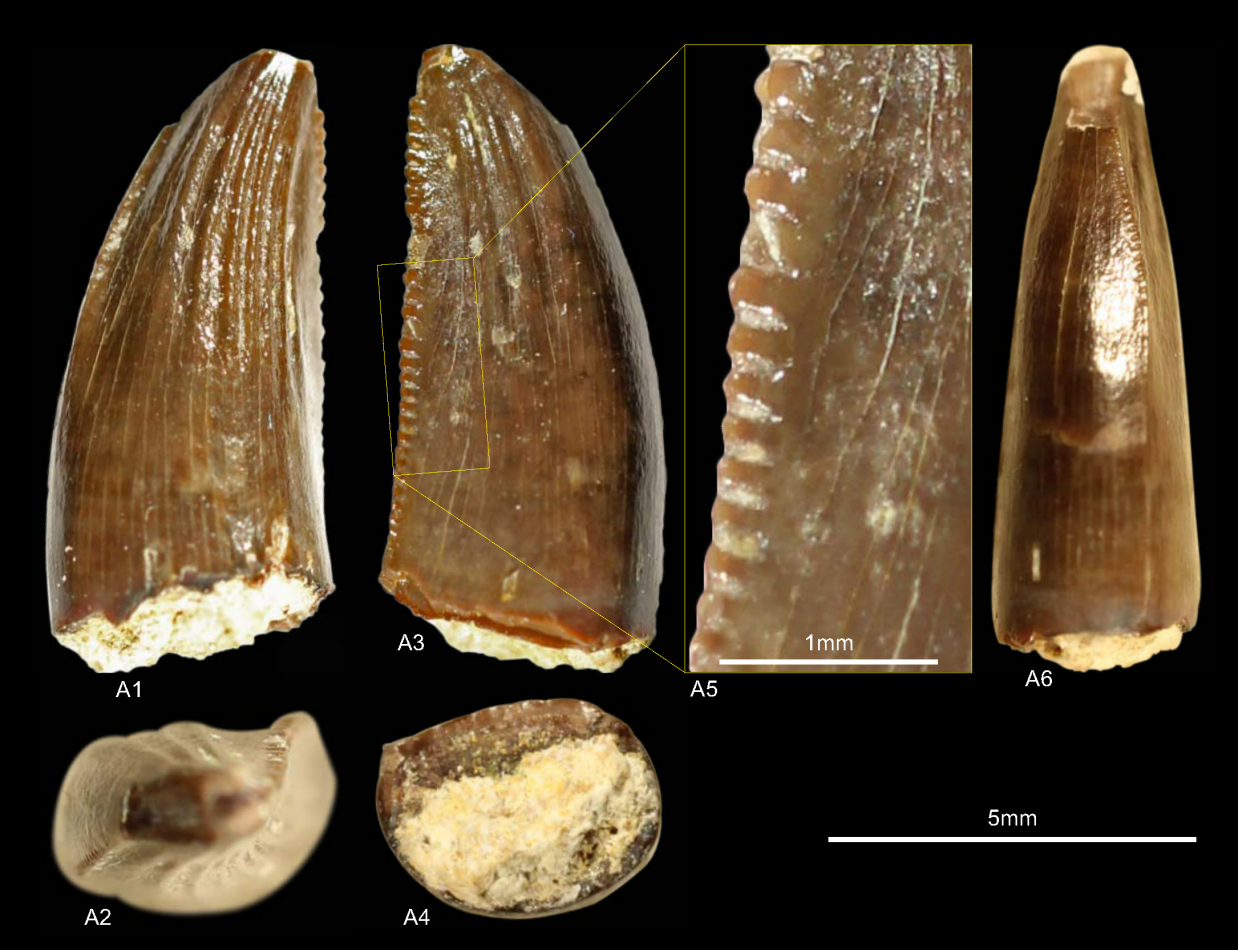
**Morphotype M:** A, **GZG.V.010.399**, A1 in lingual and A2 apical view; A3 in labial and A4 basal view, A5 details of the distal carina, A6 mesial.

GZG.V.010.399 (Fig 17)

GZG.V.010.399 was collected at the Lindener Berg in Hannover (Kimmeridgian).

#### Description

With a CH of 7 mm morphotype M is a relatively small tooth. Most obvious character of GZG.V.010.399 is a series of longitudinal grooves and ridges (flutes [1, 61]) at the upper two thirds of both sides of the crown. The lingual side possesses seven flutes and the labial three. The apex is missing and there is also a wear facet present on the upper mesial margin that obscures the starting point of the mesial carina. The finely serrated (MC 37.5) mesial carina twists lingually and terminates at midcrown. The worn vertical rectangular mesial denticles seem to be perpendicular oriented to the mesial margin and possess a symmetrical, parabolic external margin. The distal carina starts straight at the apex and twists then strongly labially and curves slightly back at the base, resulting in a well-developed sigmoid shape in distal view. The subquadrangular distal denticles (DC 22.5) are inclined apically and have an asymmetrical, parabolic-shaped external margin in lateral view. The basal cross-section is rather indifferent more subcircular; at midcrown where the twisted mesial carina terminates, it has a somewhat J-shaped condition [1]. The enamel surface is smooth and there are no interdenticular sulci visible along the mesial and distal carinae.

#### Results and Discussion

GZG.V.010.399 possesses a unique combination of features: fine lingual and coarser fluted labial side, restricted to the upper two thirds of the crown; mesial denticles considerable smaller than the distals and a smooth surface structure. For the cladistic analysis morphotype M was coded as mesialmost. When the basal cross-section is coded as subcircular this results (S4 Appendix) in a strict consensus tree that places GZG.V.010.399 within Coelurosauria, when this character is coded with? then TNT recovers it as close to *Proceratosaurus* teeth. This result is supported by the DFA (S1 Appendix) which assigns morphotype M unequivocally (98.46%) to *Proceratosaurus* mesialmost teeth. However, the most obvious difference between GZG.V.010.399 and *Proceratosaurus* mesialmost teeth is that in *Proceratosaurus* the flutes (basal striation) are restricted to the crown base [1, 75]. Fluted tooth crowns are reported for other taxa: *Paronychodon* (Late Cretaceous, North America) possesses teeth that are often devoid of denticles [112] and when present they are restricted to the distal carinae [113]; *Paronychodon*-like teeth from the Lower Cretaceous of Spain [101, 114] and the Late Jurassic of Guimarota in Portugal [101, 115, 116], these teeth have fewer coarser flutes and comparatively large distal denticles; teeth described as cf. *Paronychodon* from the Late Cretaceous of Spain [13] and Portugal (originally described as *Euronychodon* [117], transfered by Rauhut to *Paronychodon* [114]) that are devoid of denticles; in the dromaeosaurid *Velociraptor* these structures are rather rounded ridges [1, 61]; in *Ceratosaurus* premaxillary teeth they are only present on one side [118]; morphotype M resembles *Ostafrikasaurus* [119] in that the flutes are more numerous on the lingual side and seem to be also restricted to the upper two thirds of the crown. However, morphotype M differs from *Ostafrikasaurus* in possessing considerably finer denticles; distinct size difference between the mesial and distal denticles (DSDI 1.67); smooth surface structure and strongly curved, displaced carinae. The assignment of GZG.V.010.399 by the DFA and cladistic analysis to basal Tyrannosauroidea would be supported by skeletal material of the contemporary taxa *Aviatyrannis* [99] and *Juratyrant* [100]. The absence of preserved teeth in these taxa prevents a further comparison. However, Zinke [116] described teeth from the Late Jurassic of Guimarota/Portugal that well could represent the premaxillary teeth of *Aviatyrannis* [99]. Morphotype M is regarded here as Coelurosauria indet. until more complete material is available.

### Morphotype N

#### Material

**Fig 18:**
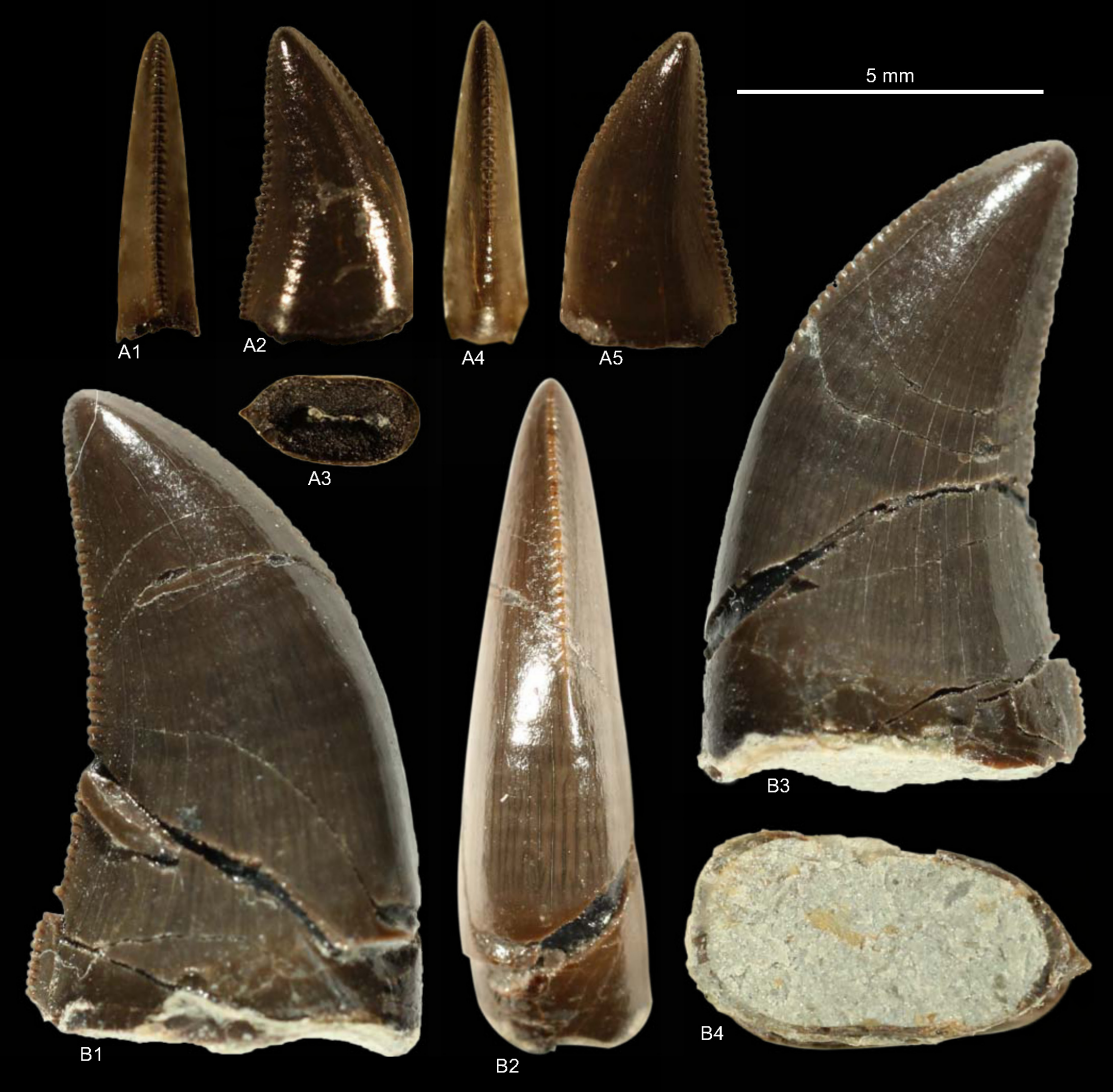
**Morphotype N:** A, **NLMH105652**, A1 in distal, A2 in lingual, A3 in basal, A4 in mesial and A5 in labial view; **B**, **DFMMh/FV382**, B1 in labial, B2 mesial, B3 lingual and B4 basal view.

DFMMh/FV382, NLMH105652 (Fig 18)

Morphotype N comprises two teeth from the Langenberg quarry near Goslar (Bed 83, Kimmeridgian). DFMMh/FV382 was like morphotype O—R included in the study of van der Lubbe et al. [16].

#### Description

Morphotype N comprises two small teeth (CH 4.9 and 10.2) that are rather stout (CHR 1.63 and 1.62) in lateral view. They have a lanceolate basal cross-section (CBR 0.50 and 0.52). The enamel surface texture of DFMMh/FV382 is obscured by hardener but seems to be braided-oriented near the apex. Morphotype N has faint, numerous (only few in NLMH105652) transversal undulations, as well as faint interdenticular sulci at the distal and mesial denticles (in DFMMh/FV382 obscured by hardener). The mesial carina terminates at midcrown and twists slightly lingually in mesial view in DFMMh/FV382, whereas in NLMH105652 it is centrally positioned on the crown. The vertical rectangular mesial denticles (MC 27.5 and 22.5) are perpendicular oriented to the mesial margin with a parabolic to flattened external margin. The distal carinae are slightly sigmoid-shaped and located at the midline in distal view. The rounded distal denticles (DC 30 and 25) are more quadrangular-shaped and perpendicular oriented to the distal margin.

#### Results and Discussion

Van der Lubbe et al. [16] regard DFMMh/FV382 as a “velociraptorine dromaeosaurid” based on, e.g. the DSDI (0.9) and the strong lateral compression (0.52). However these values are widespread in theropod teeth [61], though individual variation exists in dromaeosaurids [74]. Dromaeosaurid theropods possess an often pronounced labial depression at the cervix, an eight-shaped basal cross-section and mesial denticles that are smaller than the distals (except for *Dromaeosaurus*) [1, 61]. None of these features are present in morphotype N. Our DFA classifies (S1 Appendix) DFMMh/FV382 as *Liliensternus* (56.46%) and NLMH105652 as *Velociraptor* (82.19%). The cladistic analysis recovers (S4 Appendix) morphotype N in a strict consensus of 14 most parsimonious trees as Neotheropoda and *Piatnitzkysaurus* when coded as juvenile. When *Piatnitzkysaurus* is removed, morphotype N is classified as megalosaurid. The characteristics of morphotype N show also great agreement with morphotype B, e.g. distal carinae terminating well below the cervix, mesial carinae terminating at midcrown and mesial denticles more vertical rectangular-shaped. This assignment would be plausible, although only limited data are available of ontogenetic change in juveniles of the clade Megalosauridae. The only juvenile megalosaurid tooth material is ascribed to a hatchling or embryo of *Torvosaurus* sp. [120] where, in contrast to morphotype N, denticles are absent. Morphotype N is regarded as a possible juvenile megalosaurid.

### Morphotype O

#### Material

**Fig 19:**
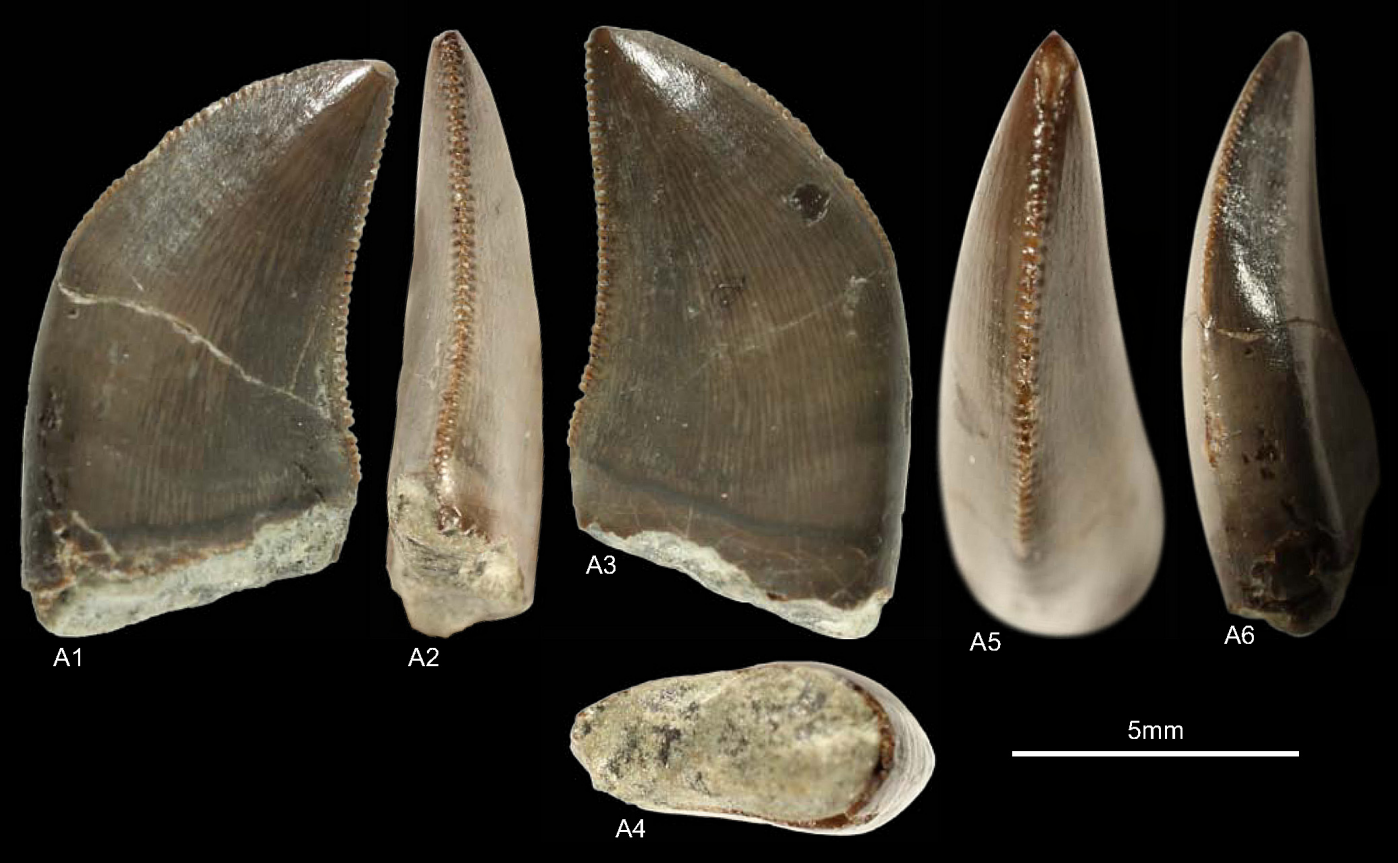
**Morphotype O:** A, **DFMMh/FV530**, A1 in lingual, A2 distal, A3 labial, A4 basal, A5 in apical and A6 oblique mesial view.

DFMMh/FV530 (Fig 19)

Morphotype O was collected at Langenberg/Goslar (Bed 83, Kimmeridgian).

#### Description

With a CH of 7.1 mm, DFMMh/FV530 is not a large tooth. It is very stout (CHR 1.2) and strongly recurved (CA 39.32°). The CBR (0.47) denotes this tooth as somewhat flattened and the basal cross-section is rather indistinctly lanceolate to eight-shaped, but the depressions, if any, on both sides are very inconspicuous. The enamel texture is obscured by hardener and seems to be braided-oriented at the apical part. Transversal undulations are like in DFMMh/FV382 numerous but faint and interdenticular sulci are present at the midcrown distal denticles. The non-twisted mesial carina terminates at midcrown where the crown becomes more recurved. There is a flat surface adjacent to the lingual side of the mesial carina. The apically inclined denticles (MC 30) are quadrangular-shaped with an asymmetrically convex external margin. The distal carina is centrally-positioned and only slightly bowed in distal view. The orientations of the midcrown distal denticles (DC 27.5) are indistinct because of the strong recurvature of the crown. They are horizontal rectangular-shaped and their external margin is slightly obscured by wear, but seems to be more asymmetrically in lateral view.

#### Results and Discussion

DFMMh/FV530 was also included in the study of van der Lubbe et al. [16] and classified as “velociraptorine dromaeosaurid” based on, e.g. the strong recurvature and DSDI in this crown. However the strong recurvature of this morphotype is not a unique feature of dromaeosaurid theropods and also found in several smaller and juvenile theropods like, e.g. *Sciurumimus* [121] and *Proceratosaurus* [75]. Morphotype O also lacks the either apically hooked or vertical rectangular mesial denticles of dromaeosaurid theropods [1, 61]. Our DFA classifies (S1 Appendix) DFMMh/FV530 as *Liliensternus* (84.79%) and on the second rank to *Proceratosaurus* (13.7%). For the cladistic analysis morphotype O was coded with an eight-shaped basal cross-section despite the fact that this character is not obvious in this tooth. TNT recovers (S4 Appendix) DFMMh/FV530 unequivocally basal to ceratosaurids even when coded as juvenile. It requires a change of several unambiguous character states to place this tooth within Dromaeosauridae. The results are insofar interesting, as dromaeosaurid taxa are well represented in the datasets for the DFA and cladistic analysis. However, the assignment of morphotype O to Ceratosauria is provisionally and could only be clarified with more complete material as no juvenile specimens are reported in this clade.

### Morphotype P

#### Material

**Fig 20:**
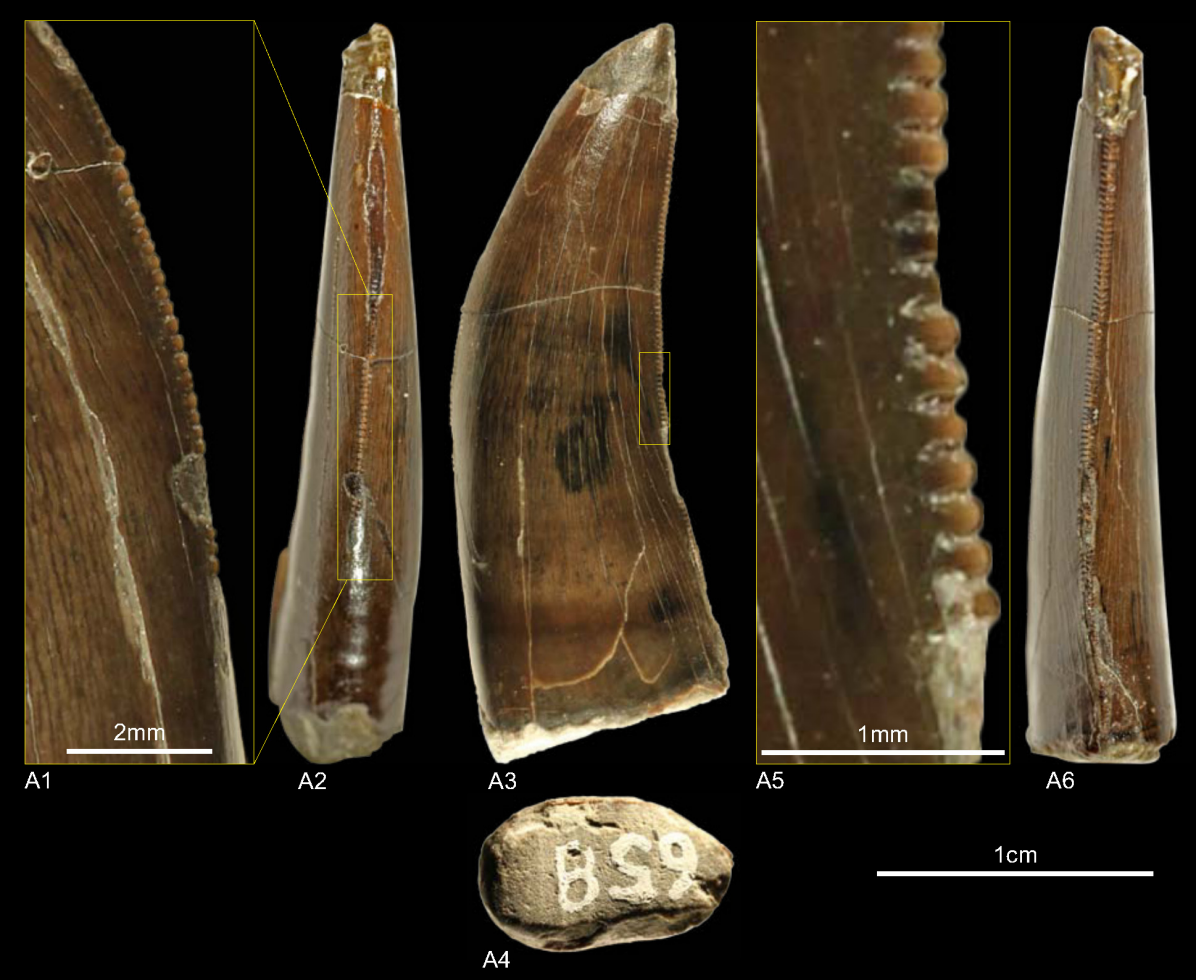
**Morphotype P:** A, **DFMMh/FV658**, A1 details mesial carina, A2 in mesial, A3 in labial and A4 basal view, A5 details of the distal carina, A6 distal view.

DFMMh/FV658 (Fig 20)

DFMMh/FV658 was collected from Bed 83 (Kimmeridgian) of Langenberg/Goslar.

#### Description

DFMMh/FV658 is a relatively complete tooth, with only the enamel at the apex and small pieces of the mesial and distal carina missing. The tooth has an irregular to smooth enamel texture. Faint interdenticular sulci are present at midcrown and basal distal denticles. The transversal undulations are sparsely distributed but well visible in lateral view. With a CH of 21.4 it is the largest crown collected at Bed 83 from the Langenberg/Goslar quarry. The CBR is 0.58 and the CHR (2.43) denotes morphotype P as slender and elongated. The basal cross-section is lanceolate. The mesial carina starts straight at the apex and twists then lingually at midcrown in mesial view. Apical denticles are not preserved on the mesial carina; the midcrowns (MC 25) are quadrangular-shaped and perpendicular-oriented to the mesial margin. The distal carina is centrally positioned and shows a sigmoid-shaped curvature in distal view. The midcrown denticles (DC 25) are more horizontal rectangular than quadrangular-shaped and the orientation is variable with some perpendicular-oriented and others slightly inclined apically.

#### Results and Discussion

The result of the DFA (S1 Appendix) is puzzling, as it classifies this tooth quite unequivocally (97.13%) as *Proceratosaurus*. *Proceratosaurus* teeth [75] are smaller, not so elongated (CHR 1.82–2.05) and possess distal denticles that are considerably larger than the mesials when compared with DFMMh/FV658 (CHR 2.43, DSDI 1). The cladistic analysis is relatively unequivocal in classifying (S4 Appendix) morphotype P close to *Eocarcharia* and therefore within Allosauroidea. This classification would be supported by skeletal material of the contemporaneous european genera *Metriacanthosaurus* [95, 96] from the Oxfordian of England and *Allosaurus* from Portugal [98]. For *Metriacanthosaurus* no teeth were reported [95, 96] and in the portuguese *Allosaurus* material only the posteriormost tooth of the maxilla is preserved, but not described [98], preventing a further comparison. DFMMh/FV658 differs from *Allosaurus* crowns of North America described by Hendrickx and Mateus [1] only in size, not labially displaced distal carina and the narrower interdenticular space of the distal denticles.

Morphotype P was also classified as “velociraptorine dromaeosaurid” by van der Lubbe et al. [16]. The subfamily Velociraptorinae then comprises the taxa *Velociraptor*, *Itemirus*, *Adasaurus*, *Tsaagan* and *Linheraptor* [122]. The teeth of *Velociraptor* and *Tsaagan* were included in the cladistic analysis presented here (S2 Appendix) and the results show (S4 Appendix) that there are no similarities between morphotype P and “velociraptorine dromaeosaurids”. Morphotype P is regarded as Allosauroidea *incerta sedis*.

### Morphotype Q

#### Material

**Fig 21:**
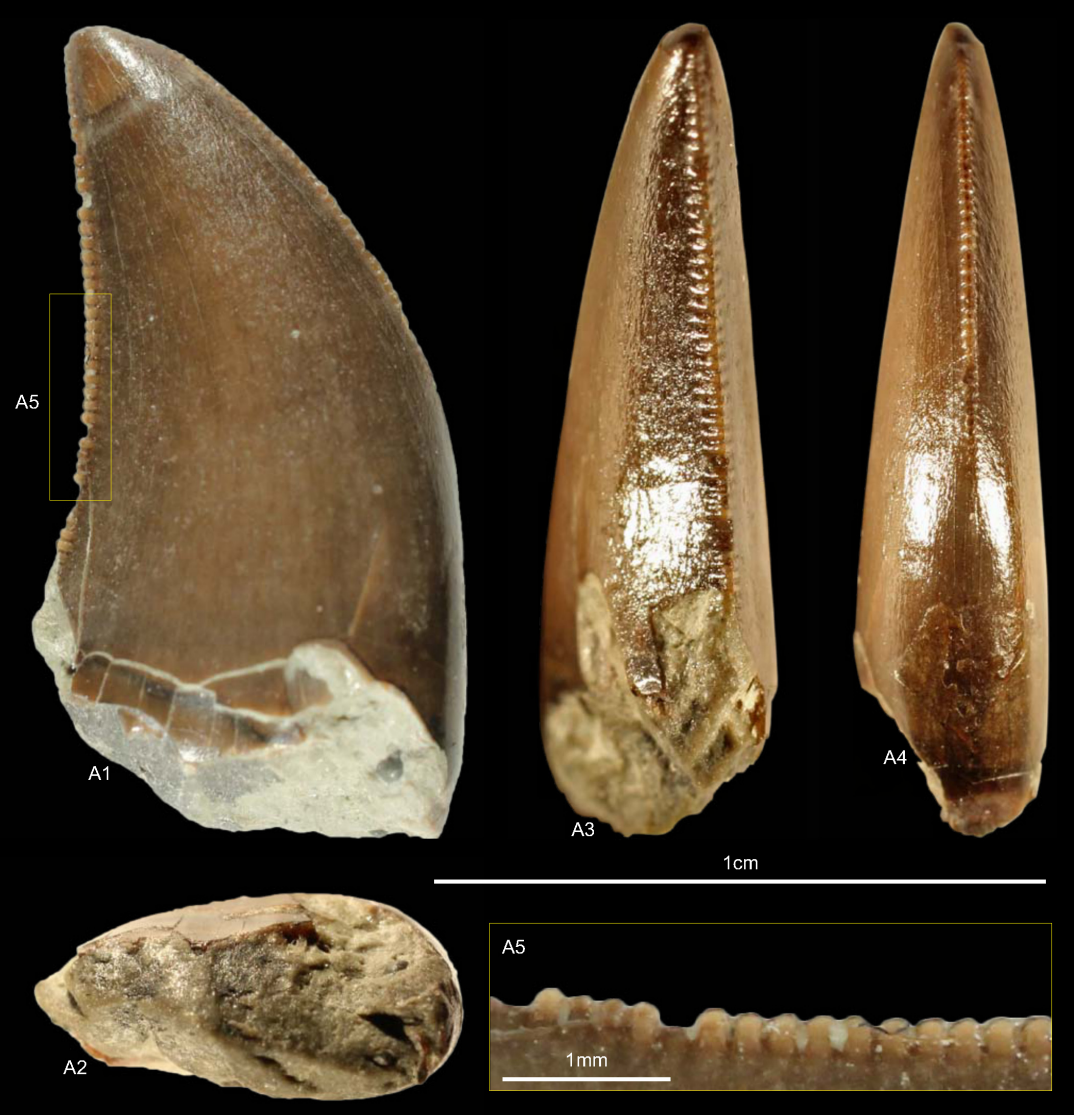
**Morphotype Q:** A, **DFMMh/FV383**, A1 in lingual, A2 basal, A3 distal and A4 mesial view, A5 details of the distal carina.

DFMMh/FV383 (Fig 21)

This specimen was collected at Langenberg/Goslar (Bed 83, Kimmeridgian).

#### Description

Morphotype Q has a CH of 10.8, is more laterally compressed (CBR 0.48) and the basal cross-section is eight-shaped. The surface texture is braided-oriented and the presence of faint interdenticular sulci is slightly obscured by hardener, as well as the rather inconspicuously transversal undulations. The non-twisted mesial carina terminates slightly above the cervix. The mesial denticles (MC 25) are worn and exhibit a rounded cross-section in mesial view. At two thirds of the crown they are rather quadrangular-shaped in lateral view. The distal carina is straight and somewhat displaced in distal view. The denticles (DC 22.5) are horizontal rectangular-shaped and perpendicular-oriented to slightly inclined apically in lateral view.

#### Results and Discussion

The results of the DFA (S1 Appendix) allocate DFMMh/FV383 quite unequivocally to *Liliensternus* (94.73%). The cladistic analysis is more ambiguous and places (S4 Appendix) DFMMh/FV383 with an unresolved strict consensus tree within Theropoda. Morphotype Q was also included in the study of van der Lubbe et al. [16] and classified as “velociraptorine dromaeosaurid”. However, morphotype Q shows many similarities with morphotype C and differs only in CH, denticle count, fewer transversal undulations and rather faint interdenticular sulci. It is therefore plausible that morphotype Q represents a juvenile of morphotype C (Ceratosauria *incerta sedis)*.

### Morphotype R

#### Material

**Fig 22:**
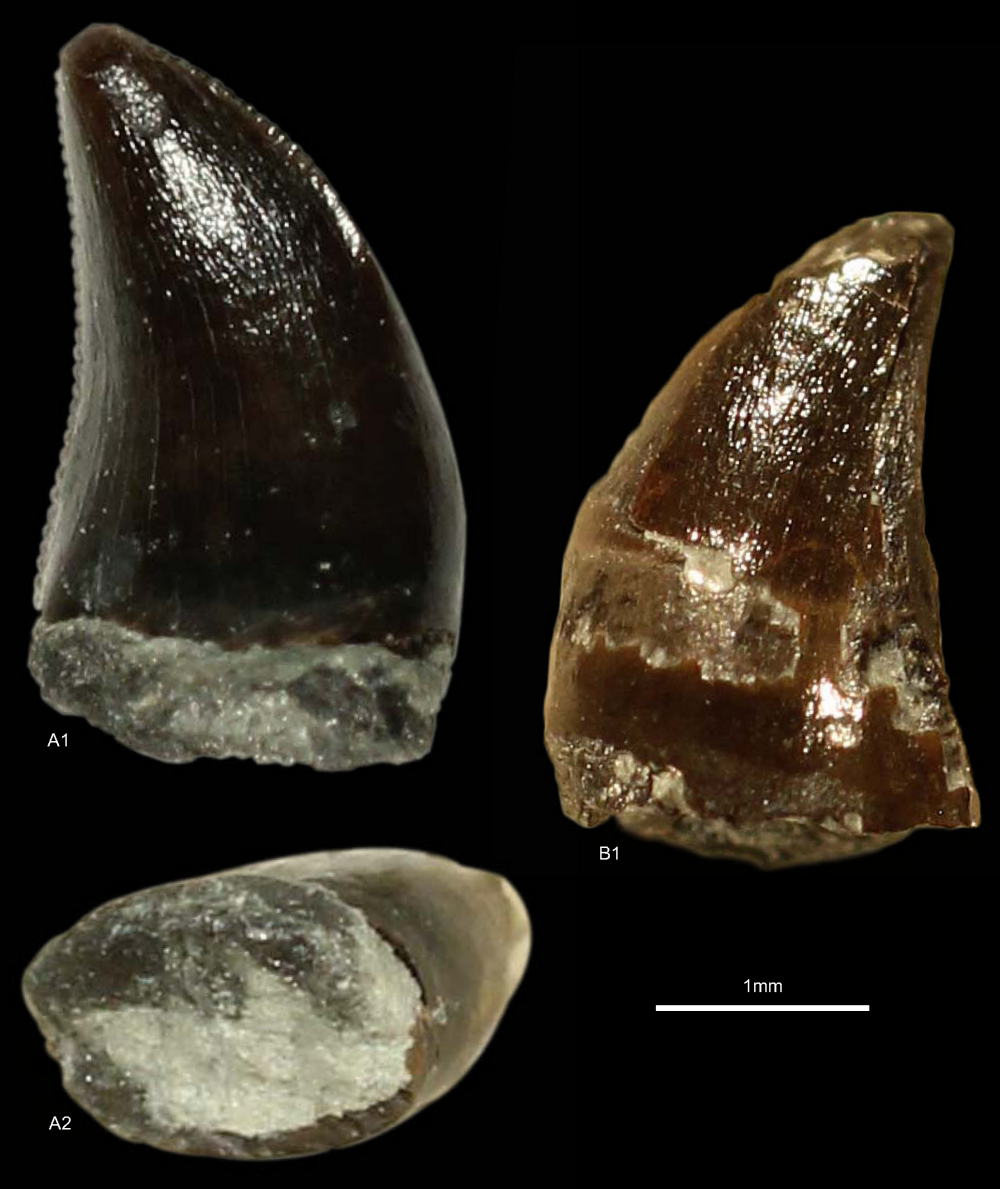
**Morphotype R:** A, **DFMMh/FV790.5,** A1 in lingual and A2 basal view; B **NLMH105654**, B1 in labial view.

DFMMh/FV790.5, NLMH105654 (Fig 22)

Morphotype R comprises two teeth from Bed 83 (Kimmeridgian) of Langenberg/Goslar.

#### Description

Morphotype R represents the smallest (CH 2.8–3) teeth in our study. NLMH105654 was recently collected by screen-washing for mammal teeth [57]. As it is very incomplete with most of the enamel layer and denticles missing only DFMMh/FV790.5 is described here. With a CBR of 0.67 and a CHR of 1.33 it is a stout-looking crown in basal and lateral view. The outer enamel layer has a braided-oriented texture and there are no interdenticular sulci and transversal undulations visible. The mesial denticles are minute (MA 65) and only present at the apical part of the crown. Their non-homogeneous shape is quadrangular to horizontal rectangular in lateral view. The mesial carina twists lingually in mesial view. The distal carina is slightly bowed and displaced in distal view. The denticles (DC 60) are quadrangular-shaped with a symmetrically parabolic external margin.

#### Results and Discussion

The DFA assigns (S1 Appendix) morphotype R to *Proceratosaurus* mesialmost teeth (92.85%). However *Proceratosaurus* mesialmost teeth exhibit basal striations [1, 61, 75], a feature that could not be considered in the DFA and therefore this assignment would be unlikely. The cladistic analysis is more indecisively and classifies (S4 Appendix) morphotype R, coded as juvenile, as Theropoda. The teeth of *Juravenator* [123–125], *Compsognathus* [1, 126] and *Sciurumimus* [121] from the Late Jurassic of southern Germany show a similar CH [61, 126] but differ from DFMMh/FV790.5 in lacking a mesial carina. As descriptions of juveniles are scarce to absent in well-known theropod taxa it is safer to follow the results of the cladistic analysis and regard morphotype R as Theropoda indet.

### Incomplete and smaller teeth not assigned to a morphotype

#### Material

**Fig 23:**
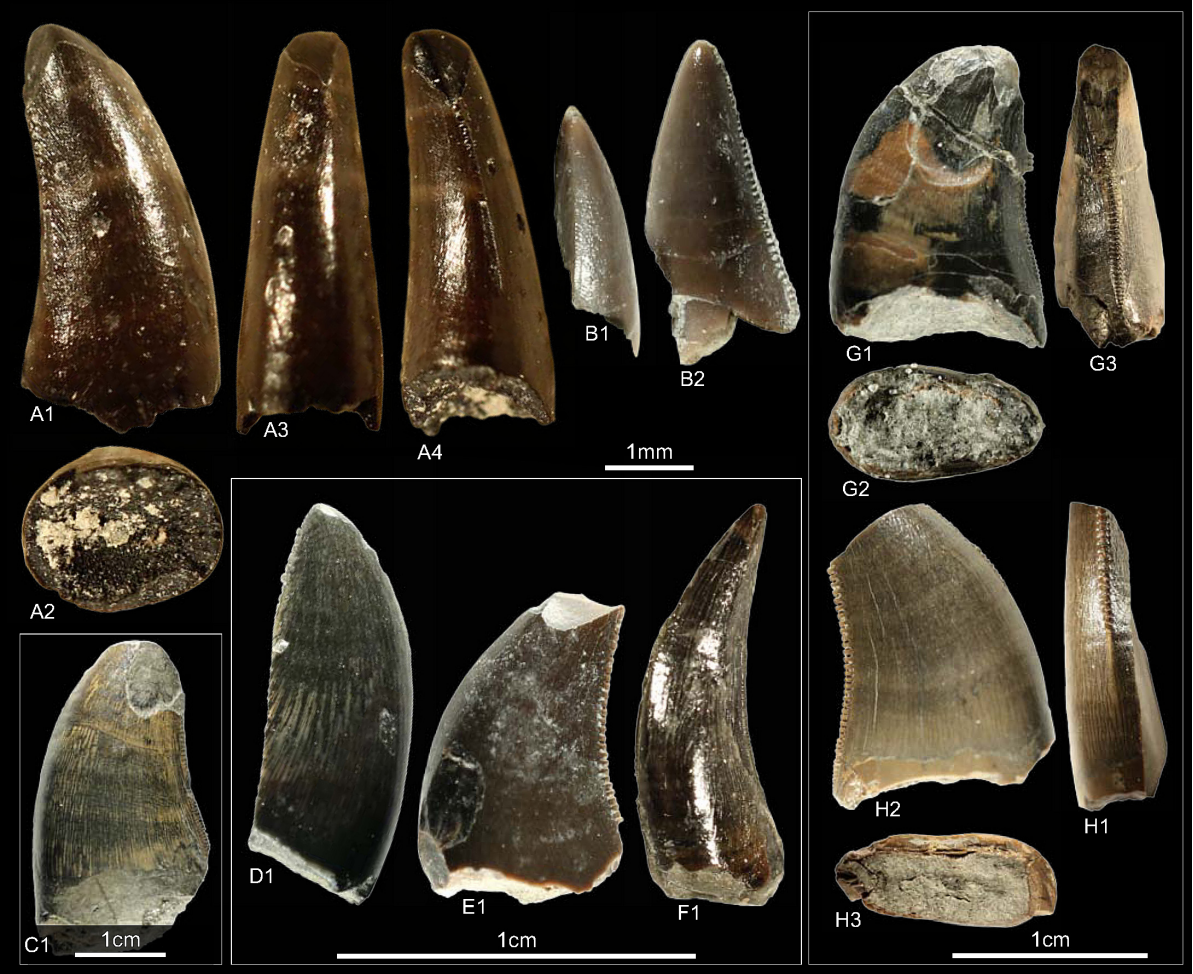
**Incomplete and smaller teeth:** A, NLMH105653, A1 in labial, A2 basal, A3 mesial and A4 distal view; B, **DFMMh/FV1202**, B1 in mesial and B2 lateral view; C, **DFMMh/FV.CL340L** in labial view; D1, **DFMMh/FV1203** in lateral view; E1, **DFMMh/FV1204** in labial view; F1, **DFMMh/FV1194** in lateral view; B, **DFMMh/FV.CL340S** B1 in lingual, B2 basal and B3 distal view; H, **DFMMh/FV707.1**, H1 in mesial, H2 labial and H3 basal view.

DFMMh/FV.CL340L, DFMMh/FV.CL340S, DFMMh/FV707.1, DFMMh/FV1194, DFMMh/FV1202, DFMMh/FV1203, DFMMh/FV1204, NLMH105653 (Fig 23).

#### DFMMh/FV.CL340L

DFMMh/FV.CL340L is a very incomplete tooth crown (CH ~30) from the Kimmeridgian of the Langenberg Quarry. The apical one third of the mesial carina and most of the distal, except for some denticles near the cervix, are worn down. However, the surface texture of both sides is well preserved and shows a braided-oriented structure with well-developed interdenticular sulci and transversal undulations. At the basal mesial carina there are also short marginal undulations present. The few preserved distal denticles are perpendicular oriented to the distal margin and have a rounded to parabolic external margin in lateral view. With a DSDI of 1 the mesial denticles are as large as the distals. The mesial carina is centrally positioned in mesial view and the distal slightly displaced labially in distal view. The basal cross-section is lanceolate with a CBR of 0.55.

The DFA classifies (S1 Appendix) DFMMh/FV.CL340L as *Ceratosaurus* premaxillary (48.44%) and on the second rank to *Duriavenator* (33.54%). The second rank classification coincides with the cladistic analysis that recovers (S4 Appendix) this tooth as megalosaurid. Given the incompleteness of DFMMh/FV.CL340L this assignment is only tentative.

#### DFMMh/FV.CL340S

DFMMh/FV.CL340S was collected in the Langenberg/Goslar Quarry (Kimmeridgian). The tooth is well preserved, except for the worn mesial denticles and apical part of the crown. There is a deep, tongue-shaped depression that terminates 3.5 mm above the cervix resulting probably from tooth contact with the opposite jaw and as the mesial and distal carinae are centrally positioned on their margins it is uncertain on which side it is located. DFMMh/FV.CL340S is relatively small (CH ~15) and has a stout appearance (CHR 1.39) in lateral view. The distal carina terminates well below the cervix. DFMMh/FV.CL340S shows many similarities with DFMMh/FV.CL340L, e.g. nearly identical CBR (0.56); the braided-oriented surface texture; well-developed transversal undulations; position of the mesial carinae on the crown and perpendicular oriented, rounded distal denticles.

The DFA classifies (S1 Appendix) DFMMh/FV.CL340S as *Dubreillosaurus* (78.07%). The cladistic analysis supports this assignment (S4 Appendix) and shows the similarities with tooth crowns of several megalosaurid genera [64].

#### DFMMh/FV707.1

DFMMh/FV707.1 was collected in the Langenberg/Goslar quarry (Kimmeridgian) and also included in the study of van der Lubbe et al. [16]. It lacks the apical third of the crown as well as the enamel and part of the dentine layer of the lingual side. This contradicts with the description of van der Lubbe et al. [16] that referred the breakage to the labial side. The only reliable measureable morphometric data are CBL (11.3), MC (13.75) and DC (12.5). The labial side of DFMMh/FV707.1 has a shallow depression at the crown base and a well preserved braided surface texture. There are also numerous faint transversal undulations present. The distal denticles terminate just below the cervix and are perpendicular oriented to the distal margin. The external margins of the denticles are parabolic to rounded in lateral view.

Based on the limited morphometric data, DFMMh/FV707.1 was only included in the cladistic analysis that recovers (S4 Appendix) it as Neotheropoda. Given the incomplete nature of this specimen it is safer to accept this result.

#### DFMMh/FV1194

This tooth was associated with bone material of the sauropod *Europasaurus* from the Langenberg Quarry (Bed 83, Kimmeridgian) and found during preparation. The lingual side is still enclosed in matrix and the visible part is highly coated with glue and hardener. The mesial and distal denticles are worn, except for some denticles located near the lower third of the distal carina, so only limited data could be recovered from DFMMh/FV1194. It is a small (CH 7.5), elongated (CHR 2.5) and recurved tooth crown. The distal denticles (DC 50) are as large as the mesials (MC 50). The distal carina has a sigmoid shape in distal view and the non-twisted mesial is centrally positioned on the mesial margin. The preserved basal distal denticles are quadrangular-shaped and have a symmetrical, parabolic external margin in lateral view.

A DFA was not conducted for DFMMh/FV1194 because of the missing CBW. The theropods *Juravenator* [123–125], *Compsognathus* [1, 126] and *Sciurumimus* [121] from the Late Jurassic of southern Germany have similar CH but lacks serrated mesial carinae. The cladistic analysis places DFMMh/FV1194 close to *Eodromaeus*. Given the limited data available for this tooth it could not be classified beyond Theropoda indet.

#### DFMMh/FV1202

With a CH of 3.5 mm, DFMMh/FV1202 is the second smallest tooth of this study and was found, like DFMMh/FV1194, during preparation of *Europasaurus* bone material. It is partly enclosed by bone, however given the fragmentary state of this specimen it must remain unclear if this bone fragment is part of the jaw in which the tooth was embedded. Most obvious feature of DFMMh/FV1202 is that the mesial margin bears a non-serrated ridge that twists lingually near the base. However, at midcrown of the carina there are three bumps visible so it remains unclear if this feature is a result of imperfect preservation. The distal carina bears well-developed, quadrangular-shaped denticles, with a symmetrical, parabolic distal margin in lateral view. The cervix is not visible as the base of the tooth is broken and the distal carina still enclosed in matrix. The distal margin of DFMMh/FV1202 is straight in lateral view. The surface texture is braided-oriented and there are no transversal undulations and interdenticular sulci visible.

The rather unserrated state of the mesial carina coincides with teeth described for the southern German theropods Juravenator [123–125], Compsognathus [1, 126] and Sciurumimus [121]. However, these theropods do not possess mesial carinae, neither serrated nor unserrated. Some metriorhynchid crocodyliforms also have a ziphodont dentition [127] and tooth crowns where the carinae are unserrated ridges comparable to DFMMh/FV1202. However, the distal carina of DFMMh/FV1202 differs from metriorhynchids in possessing well-developed denticles with well separated interdenticular space and rather gradual change in denticle size [128]. The DFA classifies (S1 Appendix) DFMMh/FV1202 unequivocally (99.99%) as Velociraptor with CBR instead of MC included. However given the fragmentary state of this crown we follow the results of the cladistic analysis that classifies (S4 Appendix) this crown as Theropoda indet.

#### DFMMh/FV1203

DFMMh/FV1203 was collected from Bed 56 [42] of the Langenberg Quarry. The tooth crown is incomplete; the upper fourth down to nearly half of the base from the distal margin missing. With a CH of 8 mm it is relatively small. The non-twisted mesial carina terminates at midcrown and the denticles are heavily worn, so only the count (MC 35) could be specified. The apical distal denticles are larger (DA 20) and worn like the mesials. The interdenticular space is narrow and bears basally turned interdenticular sulci on one side. The enamel surface is rather irregular, but this could be due to wear.

The DFA classifies (S1 Appendix) DFMMh/FV1203 as *Proceratosaurus* premaxillary (68.43%) and on the second rank as *Proceratosaurus* lateral (17.3%). The cladistic analysis recovers (S4 Appendix) it as Theropoda. This tooth exhibits several similarities with morphotype E, but is referred to morphotype E only tentatively because of its incompleteness.

#### DFMMh/FV1204

This small (CH 7.8) tooth is more complete than the previously described specimens; only the apex and small pieces near the base missing. It was also collected in the Langenberg Quarry. The mesial denticles are heavily worn and the non-twisted mesial carina terminates at midcrown. The mesial denticles (MC 27.5) are slightly smaller than the distals (DC 25). The distal denticles are horizontal rectangular-shaped, with a parabolic to rounded distal margin and perpendicular-oriented in lateral view. The distal carina is rather straight and offset from the midline in distal view. The surface structure is braided-oriented and there are only few transversal undulations visible. The interdenticular space is rather narrow and there are also faint interdenticular sulci present on the lingual side.

The DFA allocates (S1 Appendix) DFMMh/FV1204 close to *Liliensternus* (50.18%) and on the second rank similar to *Proceratosaurus* (24.07%). The cladistic analysis recovers (S4 Appendix) this tooth as similar to teeth of *Dromaeosaurus* and Tyrannosauroidea when coded as juvenile. DFMMh/FV1204 could be a member of the Coelurosauria, but as it is rather incomplete this assignment should be viewed with caution.

#### NLMH105653

NLMH105653 was like NLMH105652 and NLMH105654 recently collected while screen-washing for mammal teeth [57]. NLMH105653 is small (CH 4.5) and with a CHR of 1.96 slightly elongated. The basal cross-section is elliptical to subcircular (CBR 0.78). There are no interdenticular sulci present and the enamel surface is finely braided. The well-developed wear facet at the apex is more pronounced on the labial side. The mesial carina twists lingually and terminates at midcrown. Mesial denticles are not preserved. The distal carina strongly twists labially and terminates well above the cervix. The worn distal denticles are vertical rectangular-shaped and could only be counted at midcrown (DC 55).

The DFA classifies (S1 Appendix) NLMH105653 as *Proceratosaurus* mesialmost (72.79%). With the strongly twisted carina and elliptical to subcircular basal cross-section NLMH105653 was coded as mesialmost [1, 61] for the cladistic analysis, that classifies (S4 Appendix) it as Theropoda. NLMH105653 has a comparable CH like the southern German theropods *Juravenator* [123–125], *Compsognathus* [1, 126] and *Sciurumimus* [121]. With the vertical rectangular distal denticles, NLMH105653 resembles more the crowns of *Compsognathus* [1, 61], however, differing from this taxon in possessing a mesial carina.

## General Discussion

For the DFA we confined the number of variables and from the known ratio variables (e.g. CA, DSDI, CBR) only CHR was included instead its dependent CBL as it results in a higher reclassification rate. Keeping CBL together with CHR does not improve the results in this study. A higher number of included variables do not mean better resolution of the taxa. This is shown when a DFA on our dataset was run with all known variables (S6 Appendix) included: CBL, CBW, CH, AL, MC, DC, CBR, CHR, DSDI, CA, CAA and CDA. Half of these variables are ratio variables and already explained by their dependent variables. The reclassification rate for this DFA is 85.07% and therefore below the results (86.87%) obtained with most of the ratio variables excluded.

The examined teeth of this study were *a priori* assigned to different morphotypes because of their characteristics and this view is supported by the reclassification table of a DFA (S10 Appendix). A PCA (S10 Appendix) of these teeth also confirms this separation: The similar teeth of morphotype I and J are well separated in the morphospace, as well as morphotype C and D, with the exception of GZG.010.325 that fell within the range of morphotype C. Morphotype F and G show substantial overlap; given the similarities of these teeth this is not surprising. The differences between these morphotypes are in characters that could not be considered by morphometric measurements. Morphotype H also recovers within the latter, but as variable MC is rather uncertain this placement should be viewed with caution. A PCA (S6 Appendix) on the morphometric dataset indicates that size is an important factor in this study. This also is shown by the results of the DFA; smaller teeth are always assigned to smaller taxa. The variables CBW, CH and Al show a high positive and DC a high negative loading on the first principal component (PC1) and together PC2 accounts for 87% of the variance. The statistics (S6 Appendix) for the LDA indicate with its structure matrix that MC has a high loading on the first linear discriminant (LD1) and LD2 is composed of CBW, CH, Al and DC. Together LD1 and LD2 explain ~87% of the discrimination between the groups.

The classification power of the cladistic analysis developed by Hendrickx and Mateus [1, 61] as aid in the identification of isolated teeth is diminished by the polytomy of the strict consensus tree recovered with the updated datamatrix (Fig 3) containing only the 141 dentition-based characters. This suggests that there is more variation in the characteristics of the included theropod teeth than previously thought. The updated supermatrix (Fig 4) results in a well-resolved tree and shows the influence of the 1831 non tooth-based characters [1, 56]. However, in summary the cladistic analysis seems to be an additionally useful tool in the identification of isolated theropod teeth as it takes not only morphometric measurements into account, like in DFA, but also many non measureable characteristics.

The results of the DFA coincide in part with the cladistic analysis, especially in the larger teeth of this study (S1 Appendix). In both analyses, most problems occurred in classifying smaller teeth, as they could represent either smaller taxa or juveniles. Ontogeny in theropods and ontogenetic changes in their teeth are rather poorly understood, making the results especially of the DFA less reliable and only limited verifiable. Teeth of juvenile tyrannosaurids seem not to be simply scaled down versions of adult specimens; there is also variance in shape, including more labio-lingually compressed crowns and distal denticles larger than mesials [129, 130]. In hatchlings or embryos of *Torvosaurus* the crowns are devoid of denticles [120], whereas in adults they show comparatively coarse denticulated carinae [1, 63]. If such ontogenetic shape differences are also present in other theropod clades cannot be verified due to the limited amount of comparable specimens. However, the small teeth of morphotype N show close resemblance with the larger of morphotype B, as well as morphotype Q with morphotype C. They differ (S4 Appendix) only in the size-related characteristics CH, denticle count and the sometimes variable features less pronounced transversal undulations and interdenticular sulci. The size range of the studied teeth indicates a large variety of body sizes. The smallest crowns (morphotype R) derive from a theropod with a body length around 0.5 meter and the largest (morphotype A) probably represent individuals of 8–10 meter in length. With the possible exception that morphotype Q was a juvenile of the larger morphotype C and morphotype N a juvenile of morphotype B, it remains unclear how many of the smaller teeth represent juvenile ontogenetic stages. Northern Germany was covered frequently by a shallow epicontinental sea during the Late Jurassic [26, 27] and the vertebrates were restricted to islands so that for smaller taxa dwarfing could not be ruled out [45].

Lallensack et al. [28] recently described footprints, the largest with a mean length of 46.7 centimetres, of a large theropod from the Langenberg/Goslar quarry. They suggest a body length of around 8 meter for the trackmaker. The two large (CH 41.3 and 56) teeth of morphotype B (DFMMh/FV1205 and DFMMh/FV1206) of this study were also collected in this quarry. This theropod was probably within the same size range and could well represent the trackmaker.

The teeth summarized in the three morphotypes E–G are similar in several respects so that the conversion is sometimes obscured. They share, e.g. high DSDI (> 1.2), comparable CBR, interdenticular sulci present only at midcrown of the distal carinae and a similar surface structure. Because of this resemblance it could be assumed that these morphotypes represent the same taxon. The differences in morphology could well represent tooth position and/or ontogenetic change, with the small teeth of morphotype E probably located in more distal position of the maxilla and dentary.

Jurassic and Cretaceous theropod guilds are often dominated by a diversity of large-bodied theropods from several clades [131, 132]. The contemporaneous continental deposits of the Morrison Formation in western North America represents a well-explored large-area habitat of theropods dominated by *Allosaurus* [132], with taxa like, e.g. *Torvosaurus* as apex predator. The similar size range of the northern German theropod fauna suggests that at least during part of the Late Jurassic (Late Oxfordian -Tithonian) large land masses existed that supported a comparable theropod fauna.

The ecological sympatry of the theropod fauna from the Morrison Formation of North America and Portugal is well established [1, 63, 64, 98, 105, 114, 115] with the presence of teeth and bone material of, e.g. the genera *Torvosaurus*, *Ceratosaurus* and *Allosaurus*. This is further supported by the occurrence of basal Tyrannosauroidea remains in the Morrison Formation, Portugal and England [99, 100, 101]. The sea level highstands facilitated the invasion of new theropod taxa [28] and seems to persist long enough for speciation of these genera in Europe.

The similarities of morphotype A with teeth tentatively assigned to carcharodontosaurid remains of the Late Jurassic of Tanzania [91] and morphotype K to abelisaurid teeth from the Late Jurassic of Portugal [1] is more problematic when viewed in paleogeographical context. Carcharodontosauria and Abelisauridae seems to be restricted to the Southern Hemisphere during the Late Jurassic [91]. Hendrickx and Mateus supported the referral of the isolated teeth of Portugal to Abelisauridae with fragmentary bone material from the Late Jurassic of the Northern Hemisphere. As Rauhut [111] has shown this material probably not pertains to this clade leaving the isolated teeth of Portugal as the only assigned remains of this clade in Laurasia during the Late Jurrassic.

The resemblance of morphotype A with teeth referred to the carcharodontosaurid *Veterupristisaurus* from the Late Jurassic of Tendaguru suggests that there could have been also faunal exchange between Northern Germany and the Southern Hemisphere. Further description of the Tendaguru material is required to support this assumption. With a potentially Late Kimmeridgian age it was roughly contemporaneous to *Veterupristisaurus* the oldest member of this clade [91]. However, based on the limited material available, the referral of the teeth originally described as *Megal*osa*urus*(?) *ingens* [89, 90] to *Veterupristisaurus* is only tentatively [91] and could only be cleared by more complete material.

## Conclusions

This study reveals ecologic sympatry of several major clades of Theropoda in the Late Jurassic of Northern Germany. Basal Tyrannosauroidea, Allosauroidea, Megalosauridae and probably Ceratosauria can be established with a high probability. The presence of Dromaeosauridae [16] could not be confirmed by DFA and cladistic analysis, pending more complete material of this clade. The smaller teeth of our study show no similarities with the only slightly younger theropod fauna of Southern Germany [121, 123–126]. Results of recent screen-washing activities at the Langenberg Quarry seem to confirm this interpretation. However, this may be due to sampling bias as the teeth of *Sciurumimus*, *Compsognathus* and *Juravenator* are small and could easily be overlooked when embedded in matrix. The theropod fauna from Northern Germany shares many similarities with specimens described from Portugal (morphotype B, E, F, G, K and N) and the North American Morrison Formation (morphotype J). This could imply that at least some faunal exchange via temporary land connections between these localities existed in the Late Jurassic.

## Supporting Information

S1 Appendix Measurements, character codings of the morphotypes and results DFA theropod teeth Northern Germany. Morphometric measurements, character codings of the morphotypes and results of the DFA and classification for 80 theropod teeth from Northern Germany. Character list adopted of Hendrickx and Mateus [1, 61].
(XLS)

S2 Appendix Datamatrix. Modified datamatrix used for the cladistic analysis that is based on the supermatrix of Hendricks and Mateus [61].
(TNT)

S3 Appendix Compilation of changed character codings. Compilation of changed character codings for the data- and supermatrix of Hendrickx and Mateus [61].
(DOC)

S4 Appendix Results cladistic analysis of the morphotypes. Results cladistic analysis of the morphotypes following protocol of Hendrickx and Mateus [1, 61].
(DOC)

S5 Appendix Updated Datamatrix dentition-based characters. Modified datamatrix of Hendricks and Mateus [61] with dentition-based characters only.
(TNT)

S6 Appendix Main dataset for the DFA. Dataset with morphometric measurements for the DFA. This dataset comprises measurements of theropod teeth provided by Smith and Lamanna [74], Hendrickx et al. [64] and Rauhut et al. [75].
(XLS)

S7 Appendix Reduced dataset DFA comparison measurements Hendrickx and Smith. Reduced morphometric dataset for the comparison of overlapping taxa of Smith and Lamanna [74] and Hendrickx et al. [64].
(XLS)

S8 Appendix DFA and PCA Morphotype E–G. DFA and PCA results for morphotype E–G. Dataset that contains only taxa with a DSDI of >1.2.
(XLS)

S9 Appendix Results DFA Morphotype K. DFA results for morphotype K with isolated teeth (ML927 and ML966) described by Hendrickx and Mateus [1] included.
(XLS)

S10 Appendix DFA and PCA Morphotypes. DFA and PCA results of the morphotypes.
(XLS)

S11 Appendix 3D-View Morphotype A. MB.R.2800 of morphotype A in 3D-View.
(ZIP)

S12 Appendix 3D-View Morphotype B. GZG.V.010.381 of morphotype B in 3D-View.
(ZIP)

S13 Appendix 3D-View Morphotype C. NLMH101376a of morphotype C in 3D-View.
(ZIP)

S14 Appendix 3D-View Morphotype D. GZG.V.010.325 of morphotype D in 3D-View.
(ZIP)

S15 Appendix 3D-View Morphotype E. GZG.V.010.379 of morphotype E in 3D-View.
(ZIP)

S16 Appendix 3D-View Morphotype F. NLMH101380a of morphotype F in 3D-View.
(ZIP)

S17 Appendix 3D-View Morphotype G. NLMH101378a of morphotype G in 3D-View.
(ZIP)

S18 Appendix 3D-View Morphotype H. GZG.V.010.373 of morphotype H in 3D-View.
(ZIP)

S19 Appendix 3D-View Morphotype I. GZG.V.010.327 of morphotype I in 3D-View.
(ZIP)

S20 Appendix 3D-View Morphotype J. NLMH106235b of morphotype J in 3D-View.
(ZIP)

S21 Appendix 3D-View Morphotype K. GZG.V.010.334 in 3D-View.
(ZIP)

S22 Appendix 3D-View Morphotype L. NLMH106235a of morphotype L in 3D-View.
(ZIP)

S23 Appendix 3D-View Morphotype M. GZG.V.010.399 in 3D-View.
(ZIP)

S24 Appendix 3D-View Morphotype N. DMMhFV382 in 3D-View.
(ZIP)

S25 Appendix 3D-View Morphotype O. DMMhFV530 in 3D-View.
(ZIP)

S26 Appendix 3D-View Morphotype P. DMMhFV658 in 3D-View.
(ZIP)

S27 Appendix 3D-View Morphotype Q. DMMhFV383 in 3D-View.
(ZIP)

S28 Appendix 3D-View Morphotype R. DMMhFV709.1 of morphotype R in 3D-View.
(ZIP)
