# Supplementary material for: Multivariate and Cladistic Analyses of Isolated Teeth Reveal Sympatry of Theropod Dinosaurs in the Late Jurassic of Northern Germany
Source: PLoS One. 2016 Jul 6;11(7):e0158334. doi: 10.1371/journal.pone.0158334 (PMC4934775; doi:10.1371/journal.pone.0158334)
Supplement: S3 Appendix — Compilation of changed character codings for the data- and supermatrix of Hendrickx and Mateus [61]. (DOC) [file pone.0158334.s003.doc]

# Compilation of changed character codings in the Supermatrix of Hendrickx and Mateus [1]

The changes are mainly based, as otherwise cited, on the descriptions and data presented by Hendrickx and Mateus [1, 2, 3] and Hendrickx et al. [4]. Numeration of characters as in excel file of Hendrickx and Mateus [1, 2]. Character 34 and 63, codings wrong in most taxa that are not coded with 2. Data adopted from excel file character codings of Hendrickx and Mateus [1, 2].

**ML327**

In supermatrix of Hendrickx and Mateus [1, 2] coded as mesialmost. Data adopted from their datamatrix.

char. 85 4 ==> 3

char. 98 0 ==> 1

char. 117 1 ==> 0

**ML966**

char. 63 1 ==> 0

char. 117 1 ==> 0

***Eoraptor***

char. 66 ? ==> [01]

char. 85 2 ==> [12]

char. 98 - ==> ?

***Ceratosaurus***

char. 72 ? ==> [12]

***Genyodectes***

char. 36 2 ==> [12]

***Berberosaurus***

char. 84 3 ==> [23]

***Noasaurus***

char. 84 1 ==> [01]

char. 99 2 ==> 1

***Masiakasaurus***

char. 38 [01] ==> [12]

char. 67 [12] ==> [012]

char. 84 [12] ==> [012]

***Indosuchus***

char. 65 ? ==> 1

char. 67 ? ==> 1

***Majungasaurus***

char. 37 0 ==> [01]

char. 51 2 ==> [23]

char. 52 2 ==> [23]

***Scorpiovenator***

char. 51 ? ==> 2

***Piatnitzkysaurus***

char. 97 0 ==> - (Reference: Excel-file character codings Hendrickx and Mateus [1])

char.101 0 ==> 2

***Dubreuillosaurus***

char. 67 0 ==> [01]

***Duriavenator***

char. 84 3 ==> [34]

***Megalosaurus***

char. 84 3 ==> [34]

char. 85 3 ==> [34]

***Torvosaurus***

char. 65 2 ==> [12]

char. 66 0 ==> [01]

char. 67 2 ==> [12]

char. 84 4 ==> [34]

char. 85 4 ==> [34]

***Baryonyx***

char. 84 1 ==> [01]

***Sinraptor***

char. 97 0 ==> -

***Allosaurus***

char. 72 2 ==> [012]

char. 68 0 ==> [01]

char. 117 1 ==> 0

***Acrocanthosaurus***

char. 85 3 ==> [23]

***Carcharodontosaurus***

char. 66 0 ==> [01]

***Giganotosaurus***

char. 65 2 ==> [12]

***Proceratosaurus***

char. 65 1 ==> [01]

char. 66 ? ==> [01]

***Raptorex***

char. 67 1 ==> [01]

char. 72 0 ==> 1

***Tyrannosaurus***

char. 36 2 ==> [12]

char. 67 [12] ==> [012]

***Compsognathus***

char. 67 [12] ==> [01]

char. 72 0 ==> 1

char. 86 - ==> 1

***Scipionyx***

char. 78 1 ==> [01]

***Jianchangosaurus***

References: Pu et al. [5] and Hendrickx and Mateus [1, 2].

char. 51 ? ==> 1

char. 55 ? ==> 2

char. 56 ? ==> 0

char. 63 1 ==> 2

***Tsaagaan***

char. 78 1 ==> 0

***Velociraptor***

char. 67 1 ==> [01]

***Dromaeosaurus***

char. 66 1 ==> [01]

char. 85 2 ==> [23]

***Saurornitholestes***

char. 66 0 ==> [01]

char. 67 2 ==> 1

char. 72 0 ==> 3

char. 89 [12] ==> [01]

char. 90 - ==> 0

***Buitreraptor***

char. 67 [01] ==> [012]

***Zanabasar***

char. 99 2 ==> 1

***Troodon***

char. 89 [12] ==> 0

***Richardoestesia***

There are considerable differences in the character codings between the datamatrix and supermatrix of Hendrickx and Mateus [1, 2]. Their supermatrix is the base for the changed codings and is established upon: Bazio 1997 [6]; Currie et al. 1990 [7]; Larson and Currie 2013 [8]; Longrich 2008 [9]; Sankey et al. 2002 [10]. However, this taxon should be recoded with the original material.

char. 30 0 ==> ?

char. 63 0 ==> ?

char. 67 [012] ==> [01]

char. 71 1==> ?

char. 75 0 ==> ?

char. 82 0 ==> ?

char. 86 [12] ==> 1

char. 89 [01] ==> 0

char. 90 [02] ==> 0

char. 92 [012] ==> [01]

char. 97 1 ==> ?

char. 98 0 ==> ?

char. 99 1 ==> 0

char. 115 [02] ==> ?

char. 139 1 ==> ?

**References**

1. Hendrickx C and Mateus O. Data from: Abelisauridae (Dinosauria: Theropoda) from the Late Jurassic of Portugal and dentition-based phylogeny as a contribution for the identification of isolated theropod teeth. Dryad Digital Repository. 2014; Available: <http://dx.doi.org/10.5061/dryad.33tb2>

2. Hendrickx C and Mateus O. Abelisauridae (Dinosauria:Theropoda) from the Late Jurassic of Portugal and dentition-based phylogeny as a contribution for the identification of isolated theropod teeth. Zootaxa. 2014; 3759 (1): 1–174.

3. Hendrickx C and Mateus O.*Torvosaurus gurneyi* n. sp., the largest terrestrial predator from Europe, and a proposed terminology of the maxilla anatomy in nonavian theropods. PLoS ONE. 2014; 9(3): e88905. Available: <http://dx.doi.org/10.1371/journal.pone.0088905>

4. Hendrickx C, Mateus O and Araujo R. The dentition of megalosaurid theropods. Acta Palaeontologica Polonica. 2015; 60(3): 627-642. Available: <http://dx.doi.org/10.4202/app.00056.2013>.

5. Pu H, Kobayashi Y, Lu J, Xu L, Wu Y, et al. An Unusual Basal Therizinosaur Dinosaur with an Ornithischian Dental Arrangement from Northeastern China. PLoS ONE 2013; 8(5): e63423. doi:10.1371/journal.pone.0063423

6. Baszio S. Systematic palaeontology of isolated dinosaur teeth from the latest Cretaceous of south Alberta, Canada. Courier Forschungsinstitut Senckenberg. 1997; 196: 33–77.

7. Currie PJ, Rigby JK Jr. and Sloan RE. Theropod teeth from the Judith River Formation of southern Alberta, Canada. In: Carpenter K. and Currie PJ (Eds.) Dinosaur Systematics: Approaches and Perspectives. Cambridge: Cambridge University Press. 1990: 107–125.

8. Larson DW and Currie PJ. Multivariate analyses of small theropod dinosaur teeth and implications for paleoecological turnover through time. PLOS ONE. 2013; 8: e54329. Available: <http://dx.doi.org/10.1371/journal.pone.0054329>

9. Longrich N. Small theropod teeth from the Lance Formation of Wyoming, USA. In: Sankey, JT and Baszio S. (Eds.), Vertebrate Microfossil Assemblages. Indiana University Press. Bloomington. 2008: 135–158.

10. Sankey JT, Brinkman DB, Guenther M and Currie PJ. Small theropod and bird teeth from the Late Cretaceous (late Campanian) Judith River Group, Alberta. Journal of Paleontology. 2002; 76, 751–763.
