# Supplementary material for: Multivariate and Cladistic Analyses of Isolated Teeth Reveal Sympatry of Theropod Dinosaurs in the Late Jurassic of Northern Germany
Source: PLoS One. 2016 Jul 6;11(7):e0158334. doi: 10.1371/journal.pone.0158334 (PMC4934775; doi:10.1371/journal.pone.0158334)
Supplement: S4 Appendix — Results cladistic analysis of the morphotypes following protocol of Hendrickx and Mateus [1, 61]. (DOC) [file pone.0158334.s004.doc]

# Results from the cladistic analysis and character codings of the morphotypes

## Results from the cladistic analysis of the morphotypes

### Morphotype A


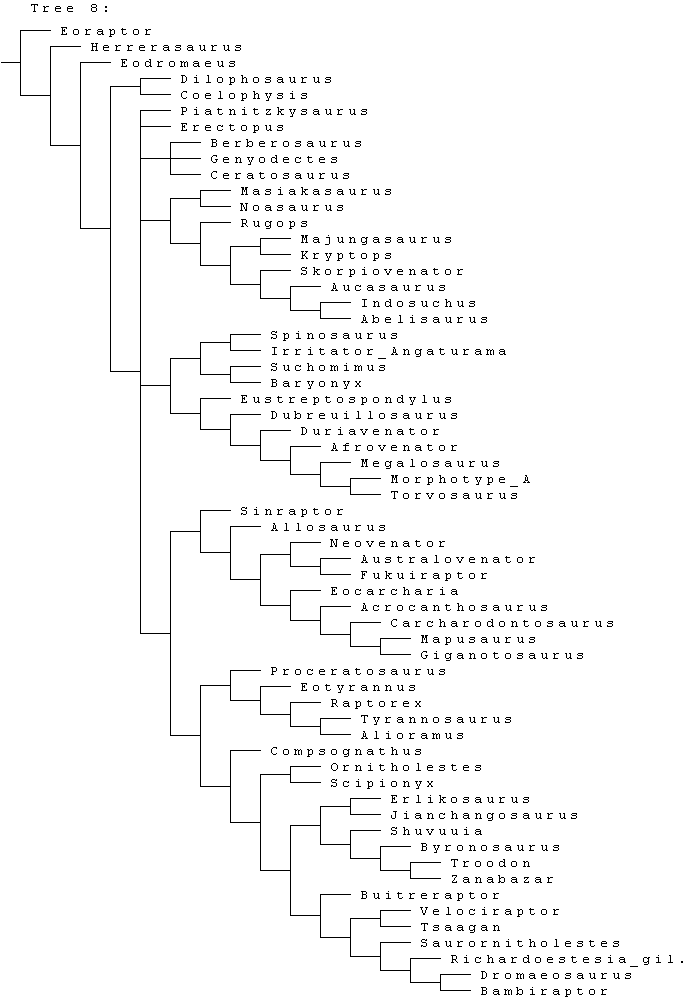


Results morphotype A : Strict consensus cladogram of eight parsimonious trees, tree length=3574, CI=0.547 and RI=0.605.

### Morphotype B

**
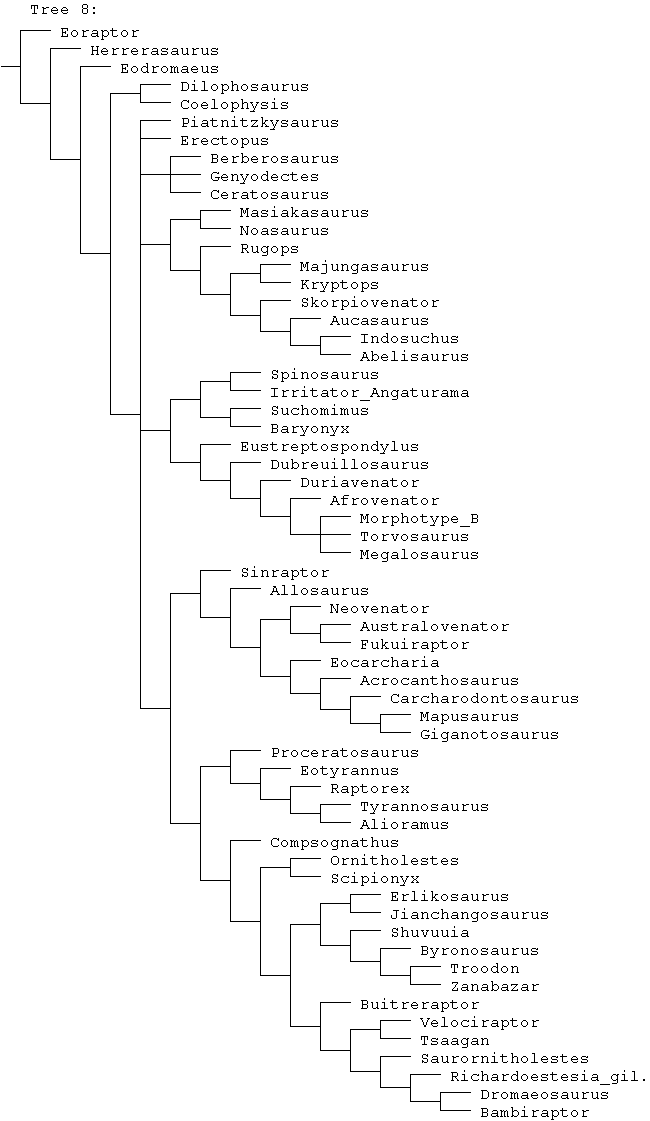
**

Results morphotype B: Strict consensus cladogram of eight parsimonious trees, tree length=3572, CI=0.547 and RI=0.605.

### Morphotype C

**
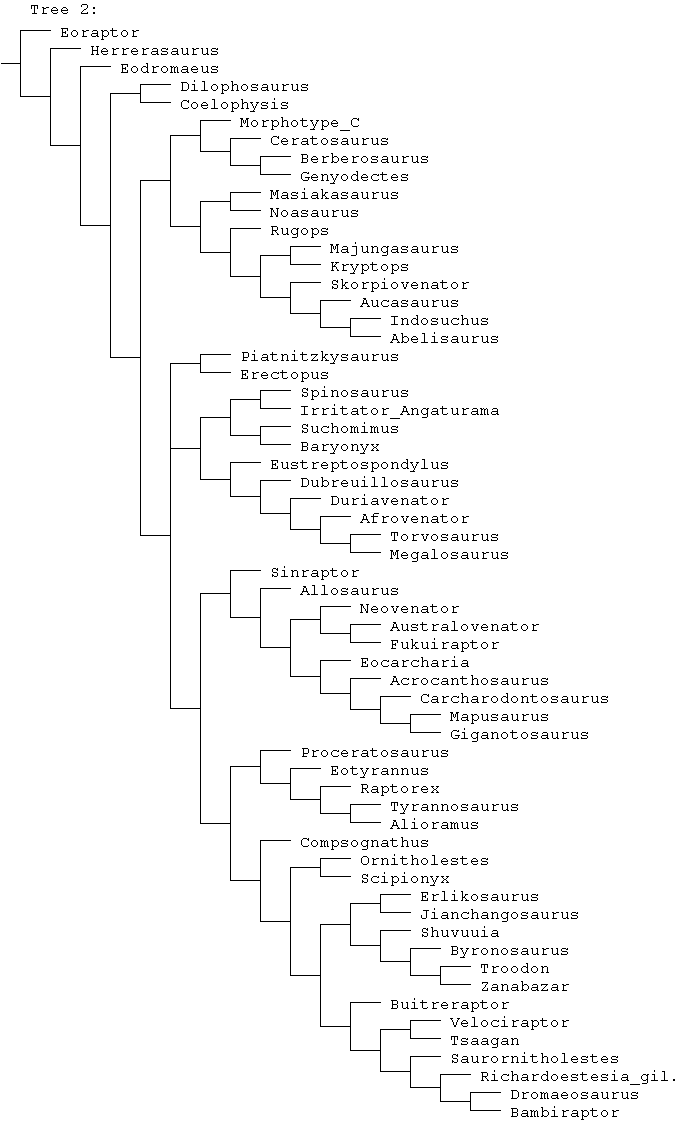
**

Results morphotype C: Strict consensus cladogram of two parsimonious trees, tree length=3577, CI=0.567 and RI=0.636.

### **Morphotype D**

Results morphotype D: Strict consensus cladogram of two parsimonious trees, tree length=3574, CI=0.567 and RI=0.636.

### **Morphotype E**

**
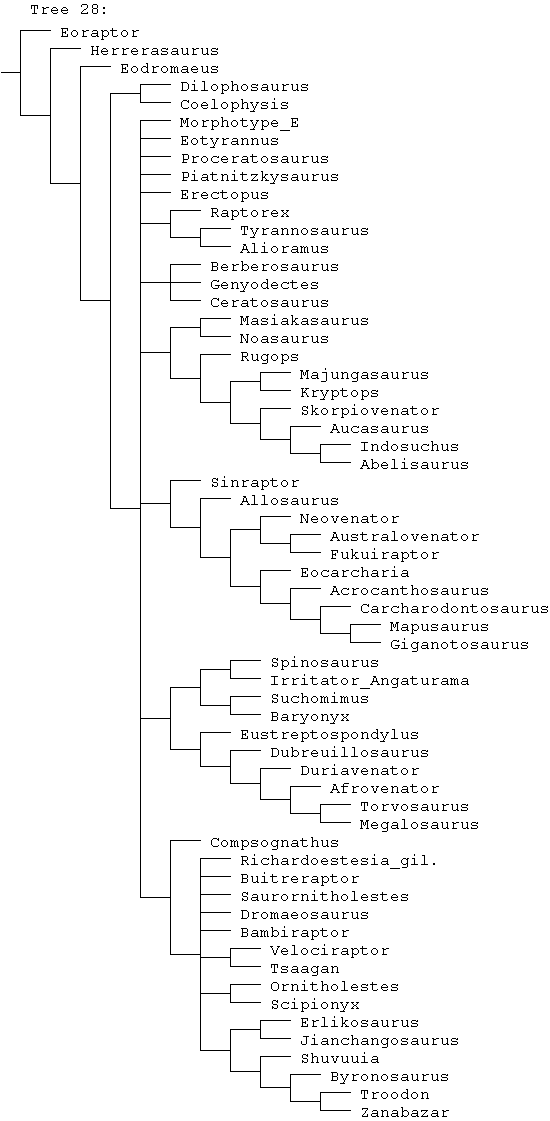

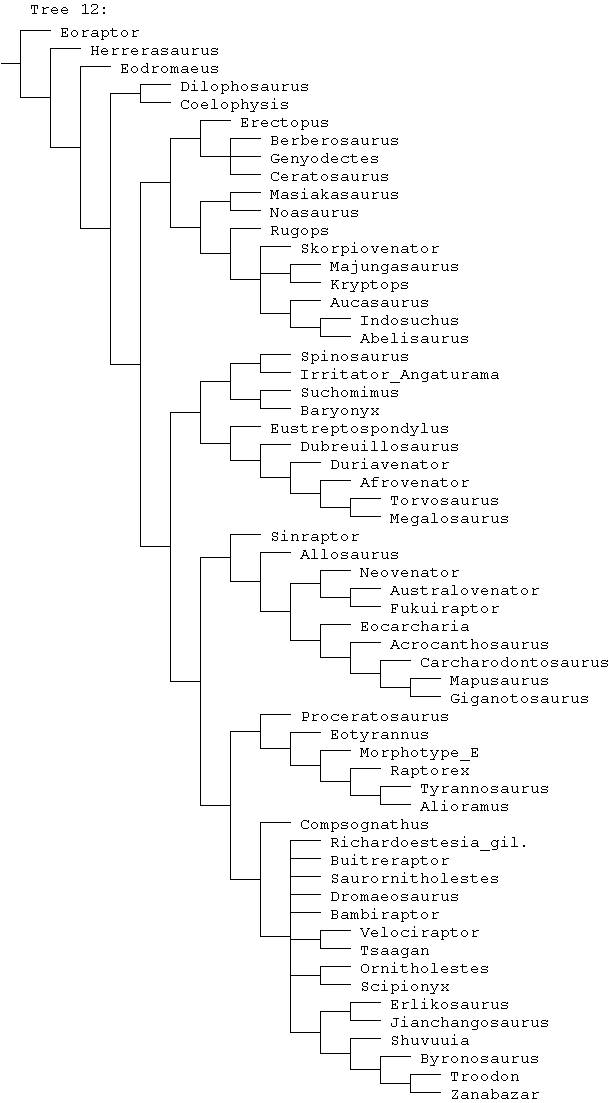
**

Results morphotype E with and without *Piatnitzkysaurus* (in parenthesis): Strict consensus cladogram of 28 (12) parsimonious trees, tree length=3573 (3523), CI=0.514 (0.556) and RI=0.549 (0.614).

### **Morphotype F**

**
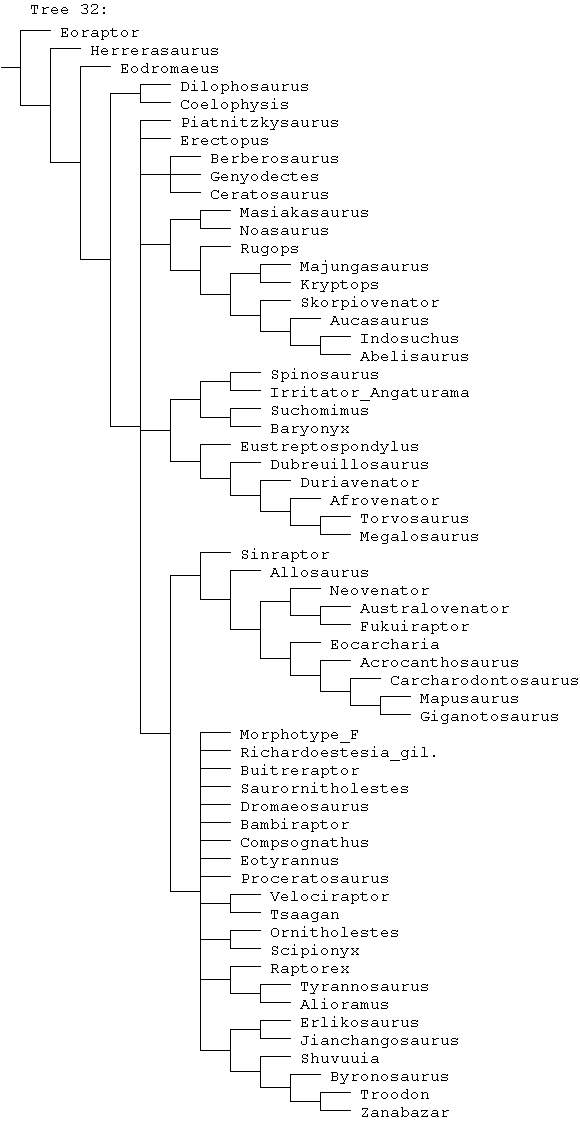
**

Results morphotype F: Strict consensus cladogram of 32 parsimonious trees, tree length=3575, CI=0.502 and RI=0.527.

### **Morphotype G**

**
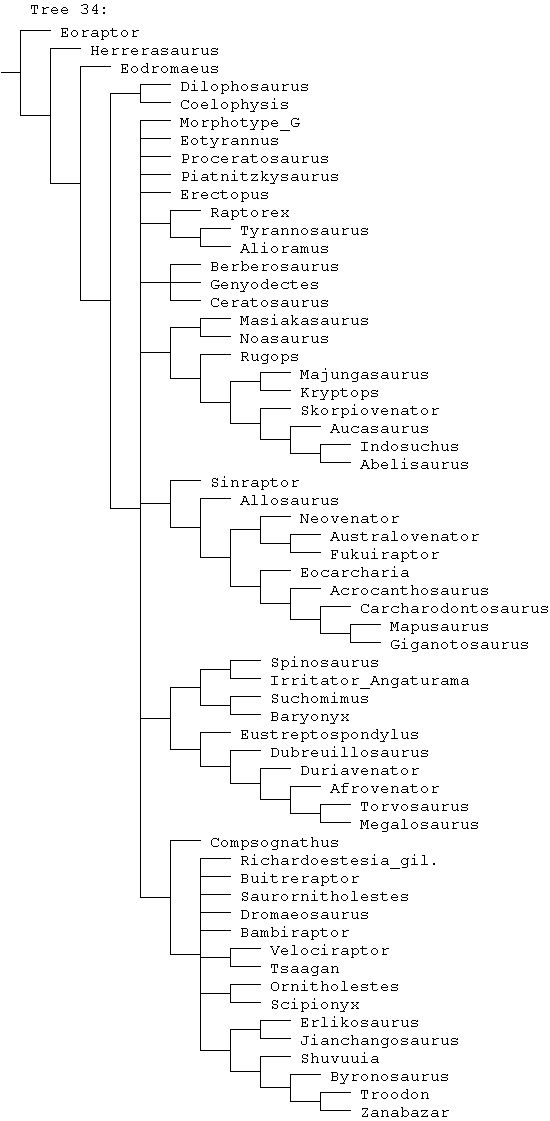

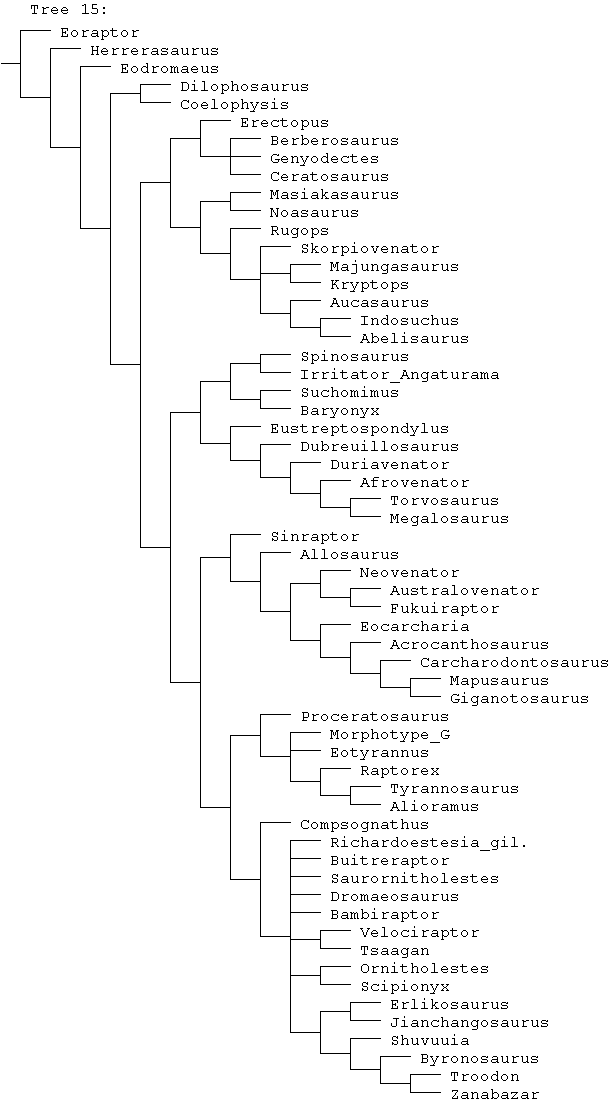
**

Results morphotype G with and without *Piatnitzkysaurus* (in parenthesis): Strict consensus cladogram of 34 (15) parsimonious trees, tree length=3574 (3524), CI=0.514 (0.556) and RI=0.548 (0.613).

### **Morphotype H**


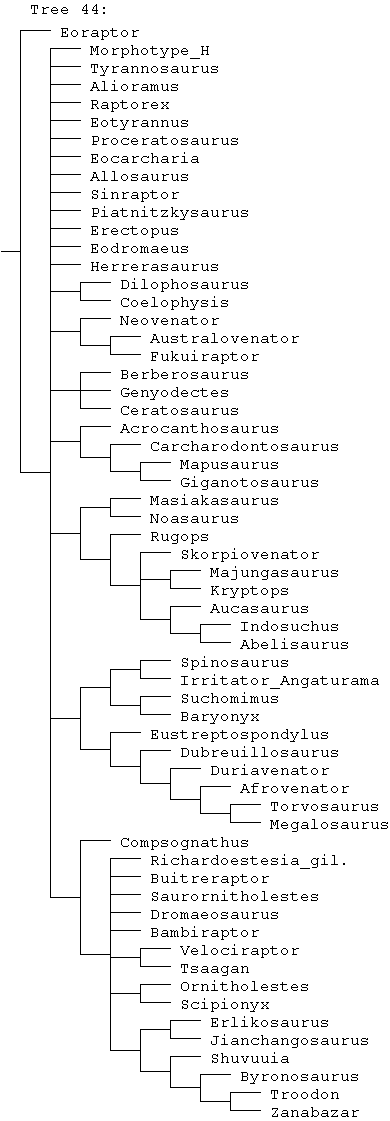

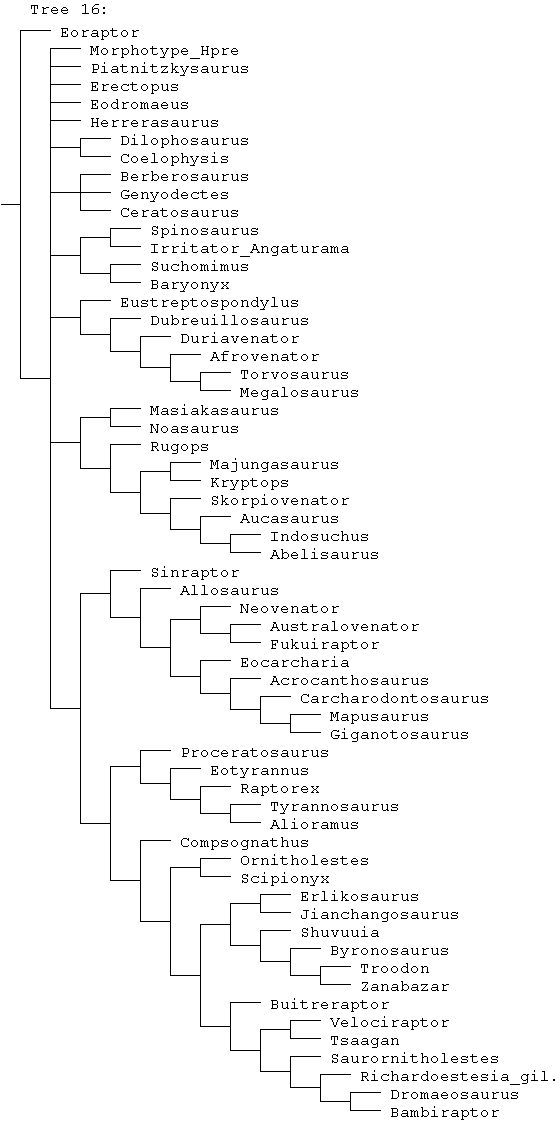


Results morphotype H coded as lateral tooth and mesialmost (in parenthesis): Strict consensus cladogram of 44 (16) parsimonious trees, tree length=3576 (3575), CI=0.45 (0.52) and RI=0.417 (0.559).

### **Morphotype E - H**


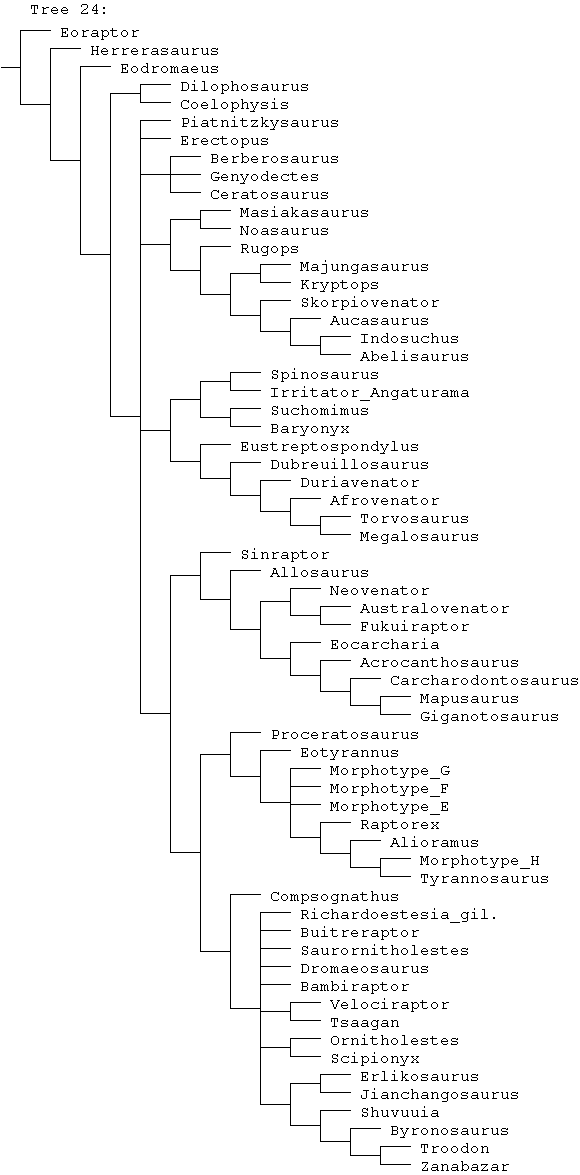


Results morphotype E - H: Strict consensus cladogram of 24 parsimonious trees, tree length=3580, CI=0.528 and RI=0.576.

### **Morphotype I**

**
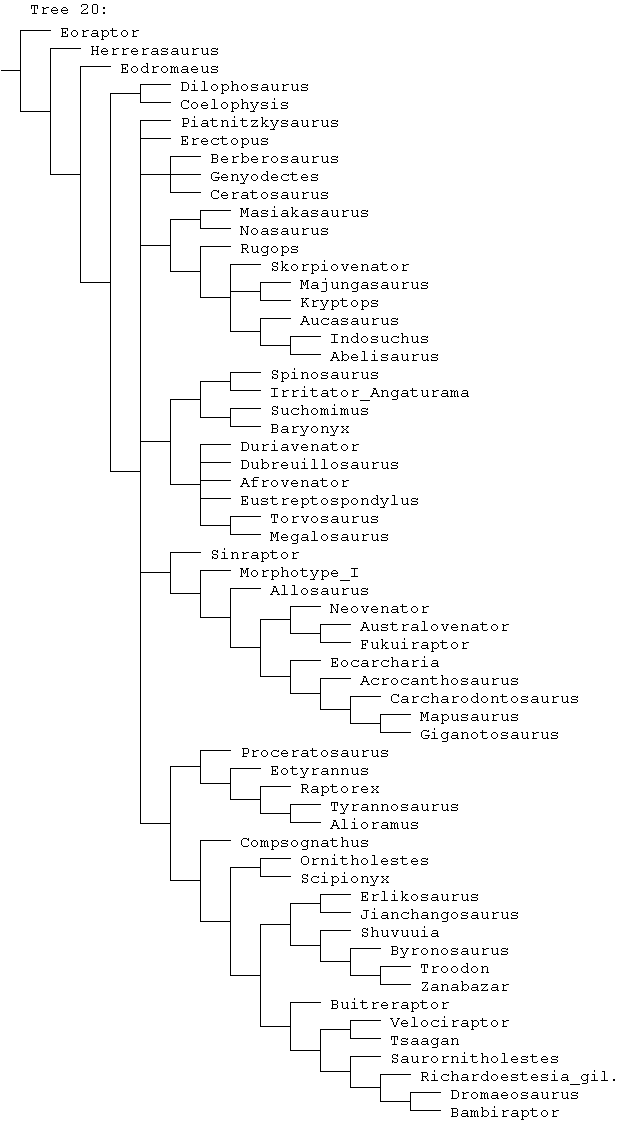
**

Results morphotype I: Strict consensus cladogram of 20 parsimonious trees, tree length=3572, CI=0.539 and RI=0.593.

### Morphotype J

**
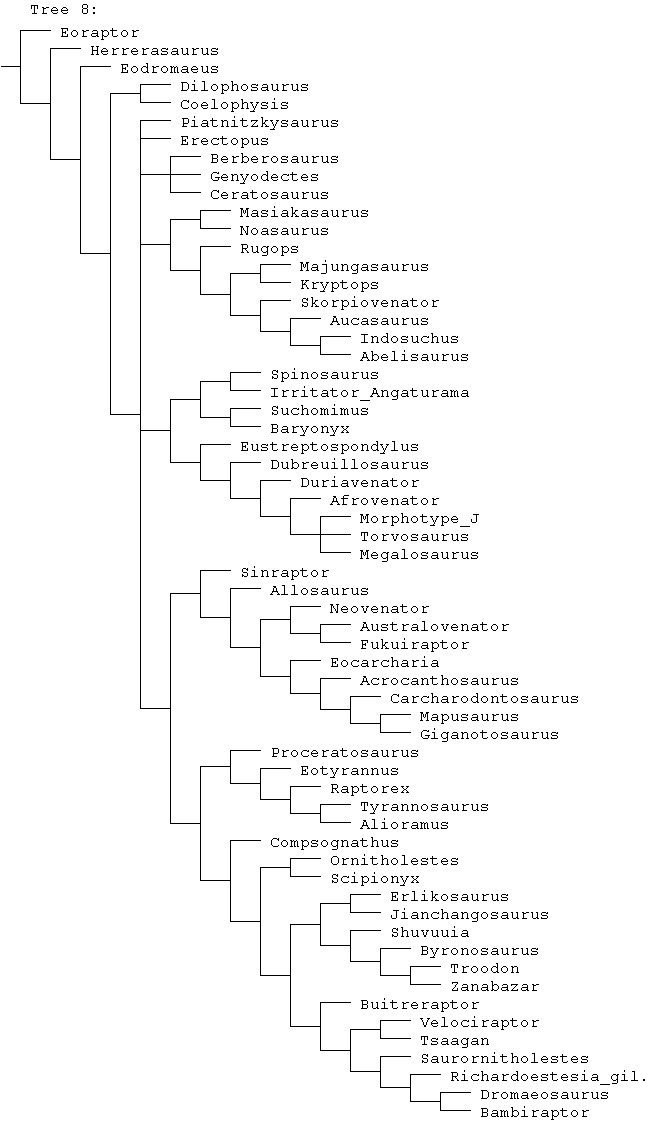
**

Results morphotype J: Strict consensus cladogram of 20 parsimonious trees, tree length=3574, CI=0.547 and RI=0.604.

### **Morphotype K**

**
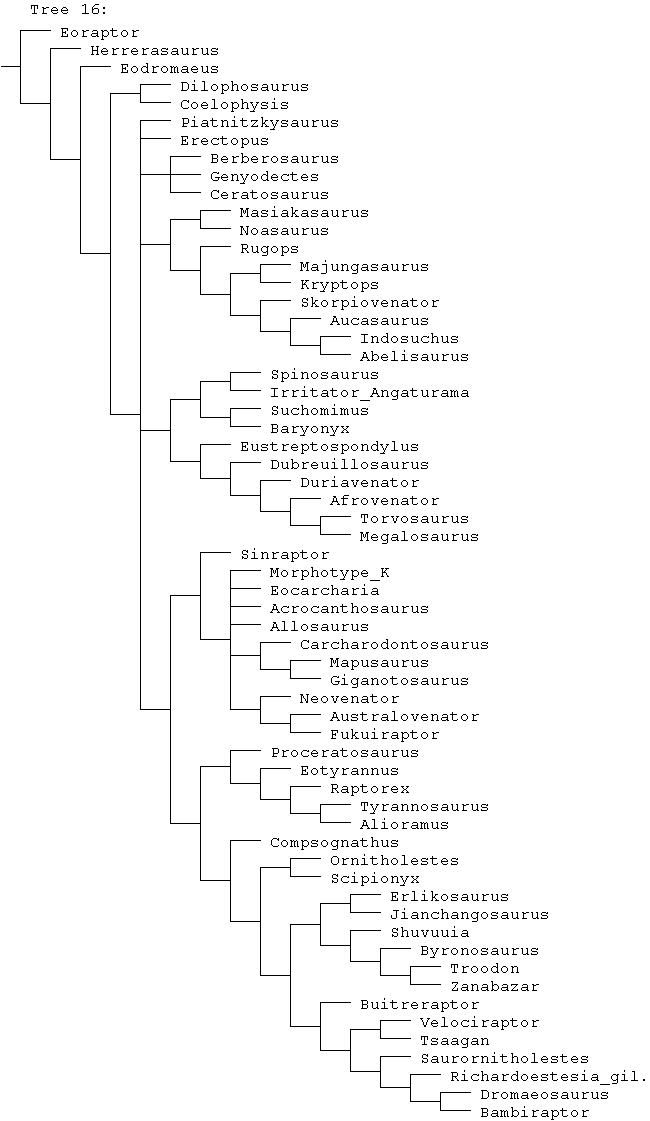

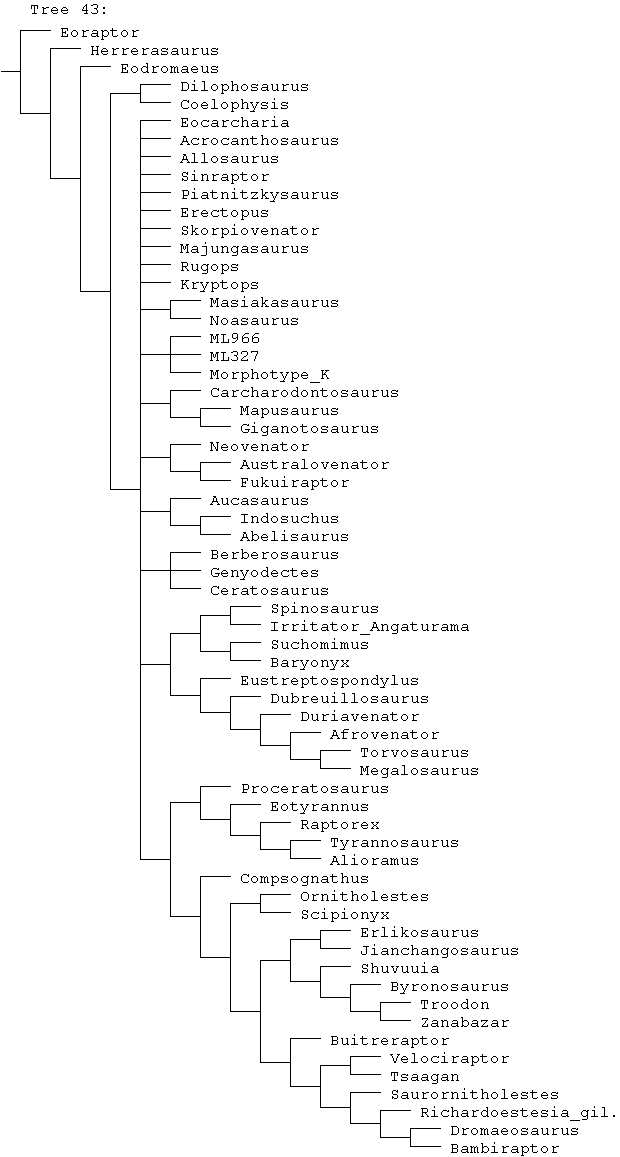
**

Results morphotype K and together with ML927 and ML966 (in parenthesis): Strict consensus cladogram of 16 (43) parsimonious trees, tree length=3578 (3590), CI=0.539 (0.482) and RI=0.592 (0.491).

### **Morphotype L**

**
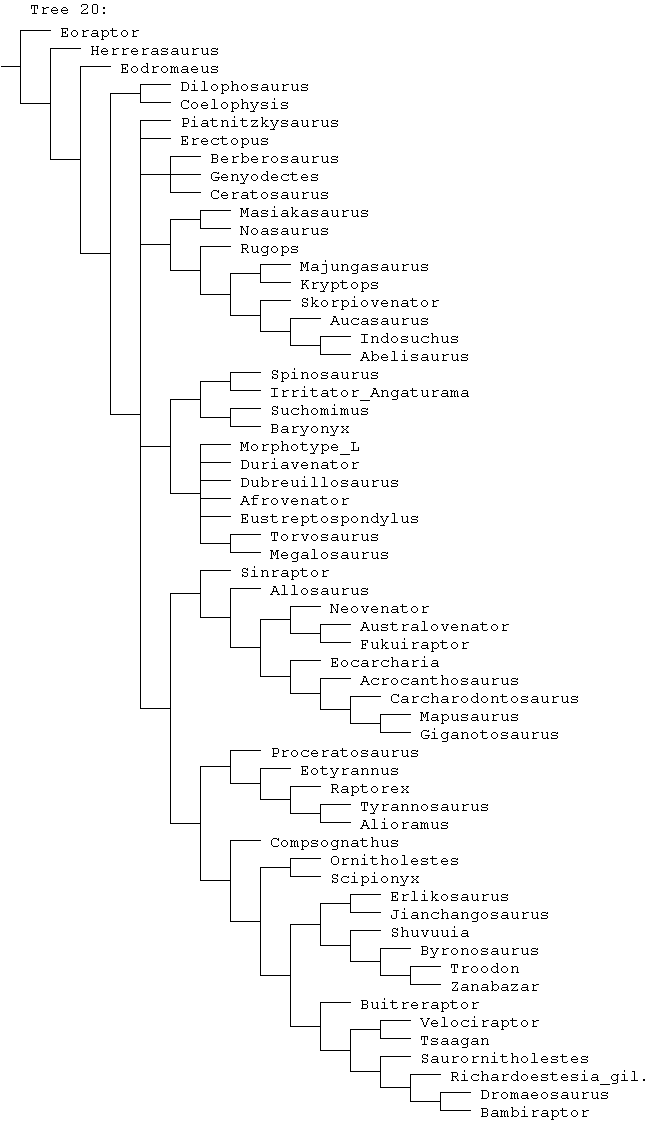
**

Results morphotype L: Strict consensus cladogram of 20 parsimonious trees, tree length=3572, CI=0.545 and RI=0.602.

### **Morphotype M**

**
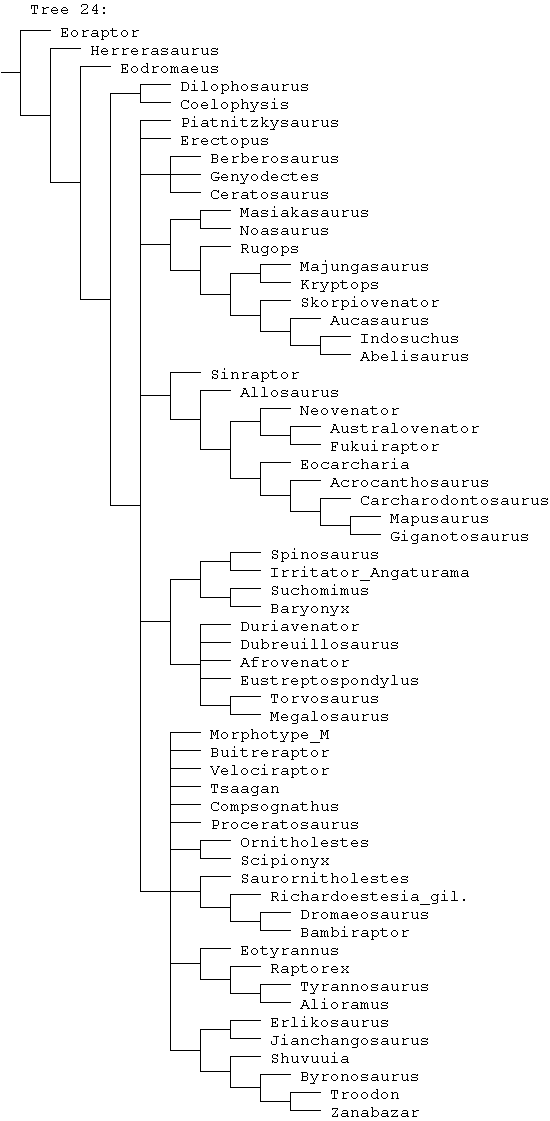
**

Results morphotype M: Strict consensus cladogram of 24 parsimonious trees, tree length=3578, CI=0.496 and RI=0.516.

### **Morphotype N**

Results morphotype N and coded as juvenile without *Piatnitzkysaurus* (in parenthesis): Strict consensus cladogram of 14(4) parsimonious trees, tree length=3575 (3524), CI=0.568 (0.575) and RI=0.638 (0.643).

### Morphotype O


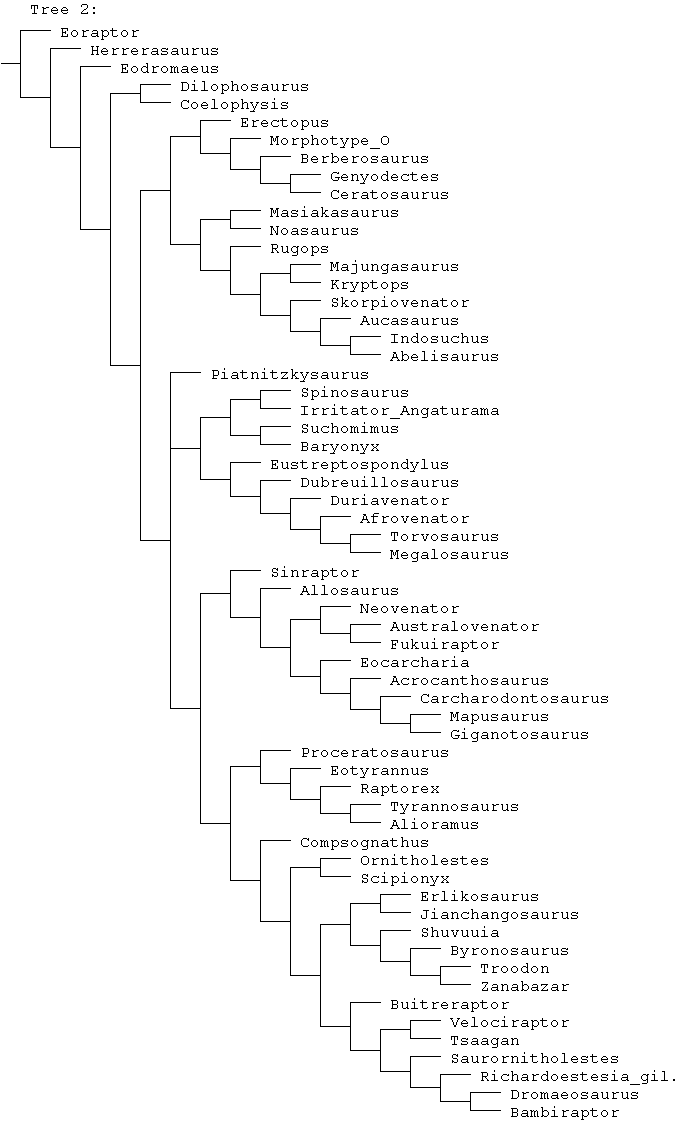


Results morphotype O: Strict consensus cladogram of two parsimonious trees, tree length=3580, CI=0.566 and RI=0.636.

### **Morphotype P**


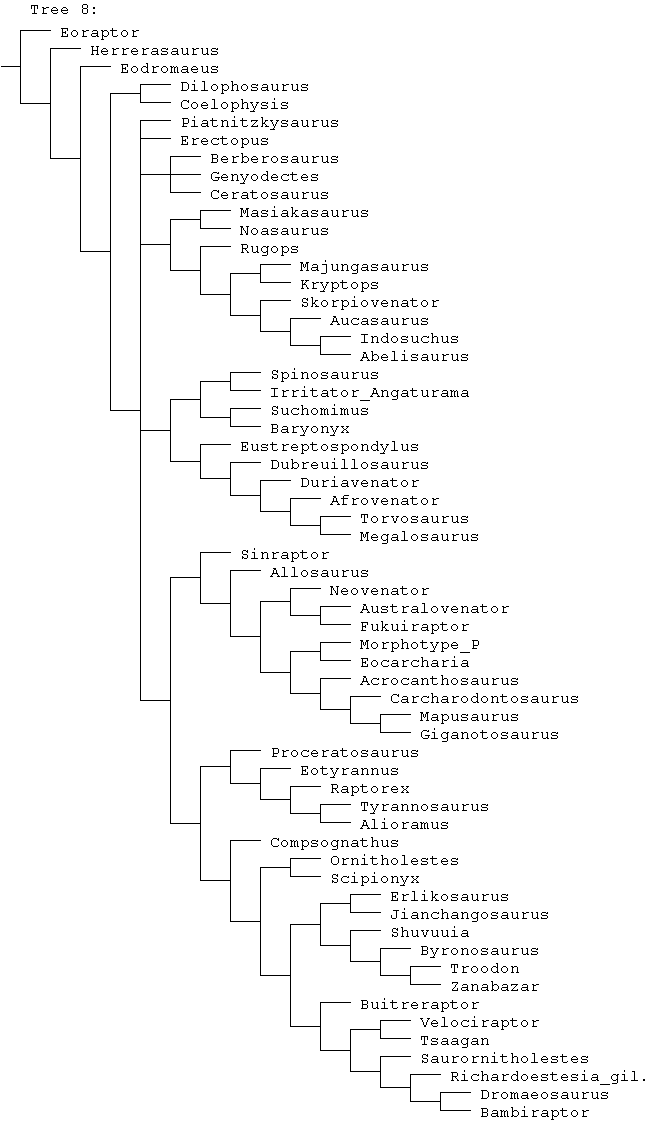


Results morphotype P: Strict consensus cladogram of eight parsimonious trees, tree length=3575, CI=0.546 and RI=0.604.

### **Morphotype Q**


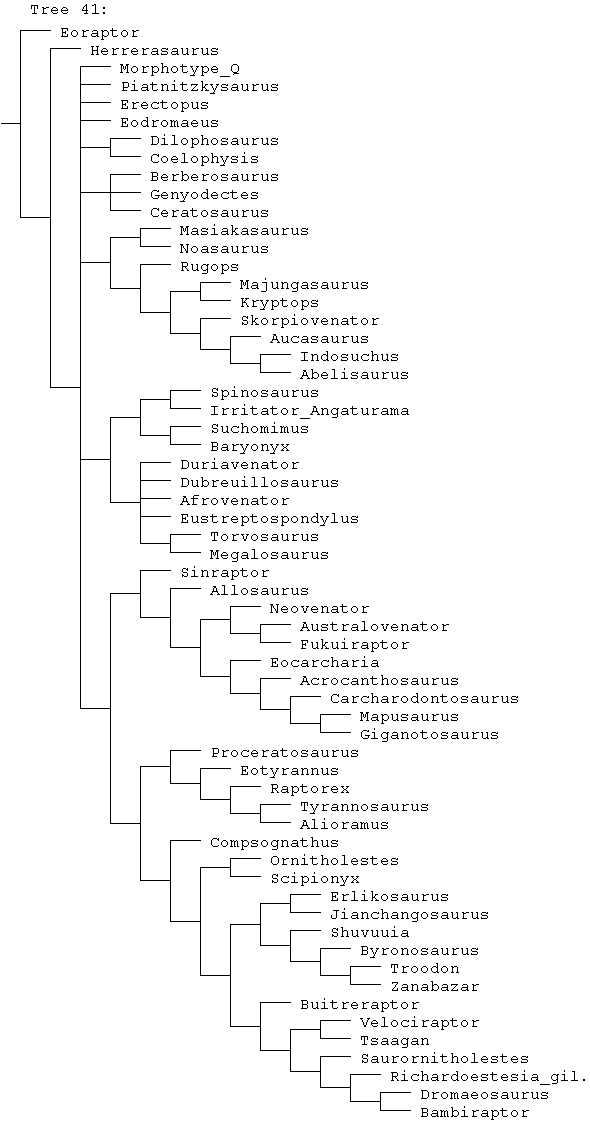


Results morphotype Q: Strict consensus cladogram of 41 parsimonious trees, tree length=3575, CI=0.535 and RI=0.585.

### **Morphotype R**


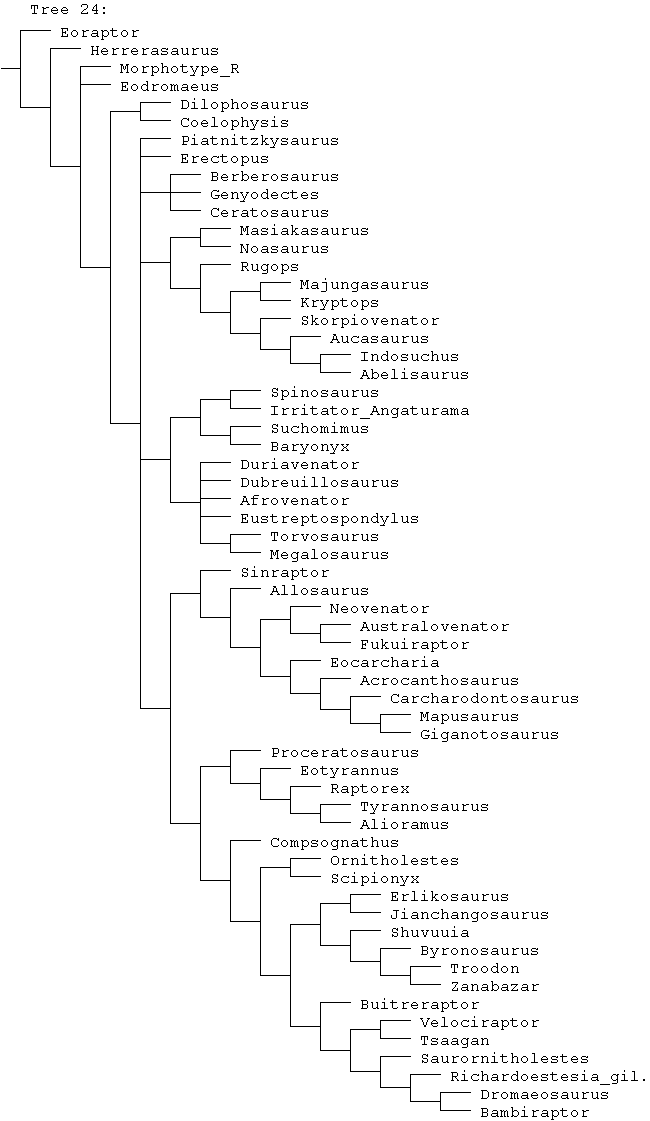


Results morphotype R: Strict consensus cladogram of 24 parsimonious trees, tree length=3577, CI=0.534 and RI=0.583.
